# Histone lysine methyltransferase structure activity relationships that allow for segregation of G9a inhibition and anti-*Plasmodium* activity†
†The authors declare no competing interests.
‡
‡Electronic supplementary information (ESI) available: Supplementary Tables ST1–ST5, experimental data for the representative diaminoquinazoline analogues, 2D NMRs of **85** and **111a** and X-ray structure of **111a** (Fig. SF1). The coordinates for **111a** have been deposited with CCDC 1503377. For ESI and crystallographic data in CIF or other electronic format see DOI: 10.1039/c7md00052a


**DOI:** 10.1039/c7md00052a

**Published:** 2017-03-15

**Authors:** Sandeep Sundriyal, Patty B. Chen, Alexandra S. Lubin, Gregor A. Lueg, Fengling Li, Andrew J. P. White, Nicholas A. Malmquist, Masoud Vedadi, Artur Scherf, Matthew J. Fuchter

**Affiliations:** a Department of Chemistry , Imperial College London , London SW7 2AZ , UK . Email: m.fuchter@imperial.ac.uk ; Fax: +44 (0)2075945805 ; Tel: +44 (0)2075945815; b Unité Biologie des Interactions Hôte-Parasite , Département de Parasites et Insectes Vecteurs , Institut Pasteur , Paris 75015 , France; c CNRS ERL 9195 , Paris 75015 , France; d INSERM Unit U1201 , Paris 75015 , France; e Structural Genomics Consortium , University of Toronto , Toronto , ON M5G 1L7 , Canada; f Department of Pharmacology and Toxicology , University of Toronto , Toronto , ON M5S 1A8 , Canada

## Abstract

*Plasmodium falciparum* HKMTs (PfHKMTs) play a key role in controlling *Plasmodium* gene expression and represent exciting new anti-malarial epigenetic targets. Using an inhibitor series derived from the diaminoquinazoline HKMT inhibitory chemotype, we have previously identified compounds with highly promising antimalarial activity, including irreversible asexual cycle blood stage-independent cytotoxic activity at nM concentrations, oral efficacy in *in vivo* models of disease, and the unprecedented ability to reactivate dormant liver stage parasites (hypnozoites). However, future development of this series will need to address host *versus* parasite selectivity, where inhibitory activity against human G9a is removed from the lead compounds, while maintaining potent anti-*Plasmodium* activity. Herein, we report an extensive study of the SAR of this series against both G9a and *P. falciparum*. We have identified key SAR features which demonstrate that high parasite *vs.* G9a selectivity can be achieved by selecting appropriate substituents at position 2, 4 and 7 of the quinazoline ring. We have also, in turn, discovered that potent G9a inhibitors can be identified by employing a 6-carbon ‘Nle mimic’ at position 7. Together, this data suggests that while broadly similar, the G9a and potential PfHKMT target(s) binding pockets and/or binding modes of the diaminoquinazoline analogues exhibit clear and exploitable differences. Based on this, we believe this scaffold to have clear potential for development into a novel anti-malarial therapeutic.

## Abstract 2

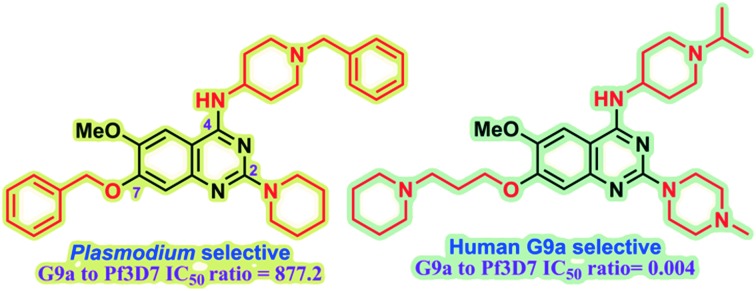
We identify key SAR features which demonstrate that high parasite *vs.* G9a selectivity can be achieved for the quinazoline inhibitor chemotype.

## Introduction

Despite a global reduction in malaria incidence and mortality rate over the past 15 years, this disease remains a major health burden, with over 200 million new cases reported in 2015.^1^ The majority of the malaria related deaths occur in sub-Saharan Africa, particularly amongst children under five and pregnant women. The emergence of multi-drug resistant strains of *Plasmodium*, the malaria causing parasite, have rendered many of the conventional antimalarial drugs ineffective.^2^,^3^ Artemisinin-based combination therapies (ACTs) have, in part, addressed this issue and are widely used as an effective treatment.^4^ However, recent reports of artemisinin resistant *Plasmodium* strains along the Thai–Cambodian border^5^,^6^ is of significant concern. Thus, there is an increasing demand for the discovery of novel classes of antimalarial drugs, with distinct modes of action, in order to tackle multi-drug resistant *Plasmodium* parasites.^7^

Epigenetic regulation has been shown to affect gene expression throughout the life cycle of *Plasmodium*^8^,^9^ and thus, modulation of epigenetic targets in the malarial parasite presents a novel approach for antimalarial drug discovery. Indeed, inhibitors of malarial histone deacetylases (PfHDACs) have been shown to possess parasite killing activity and are currently being explored as a new class of antimalarial drugs.^10^–^14^ However, the poor selectivity, unfavourable pharmacokinetics and toxicity issues associated with the hydroxamic acid-based HDAC inhibitors poses a major challenge to their clinical development.^15^ Thus other potential epigenetic targets in *Plasmodium* are of significant interest.

Histone lysine methyltransferases (HKMTs) act as vital components of epigenetic regulation by serving as ‘writers’ that install methyl marks on histones and other proteins. Among various human HKMTs, G9a (EHMT2) is a well-studied enzyme, which catalyses the addition of one or two methyl groups to lysine 9 of histone H3 (H3K9me1 and H3K9me2).^16^ Like most of the other HKMTs, the active site of G9a resides in the SET (suppressor of variegation 3–9, enhancer of zeste and trithorax) domain, where the substrate peptide binds and is, in turn, methylated by an *S*-adenosyl methionine (SAM) cofactor. G9a has been shown to play key role in various physiological and pathophysiological processes such as mental health,^17^ cocaine addiction,^18^,^19^ differentiation and cancer^20^–^22^ and thus together with other HKMTs, it is under investigation in context of drug discovery.^23^–^26^

*Plasmodium falciparum* HKMTs (PfHKMTs) play key role in controlling *Plasmodium* gene expression through epigenetic pathways.^9^ Computational analysis predicts the presence of ten SET domain containing PfHKMTs,^27^ six of which were found to be essential in the asexual blood-stages of the parasite and thus may represent good drug targets.^28^,^29^ Moreover, knockout of PfSET2 (now renamed PfSETvs) was found to reverse the silencing of the *var* gene family, which is centrally involved in the immune evasion mechanism by which *Plasmodium* avoids the host antibody response.^8^,^28^ Despite this potential, production of enzymatically active PfHKMTs has proved to be challenging, with only a few successful reports in the literature,^29^,^30^ thus hindering the prospect of PfHKMT inhibitor discovery.

**Fig. 1:**
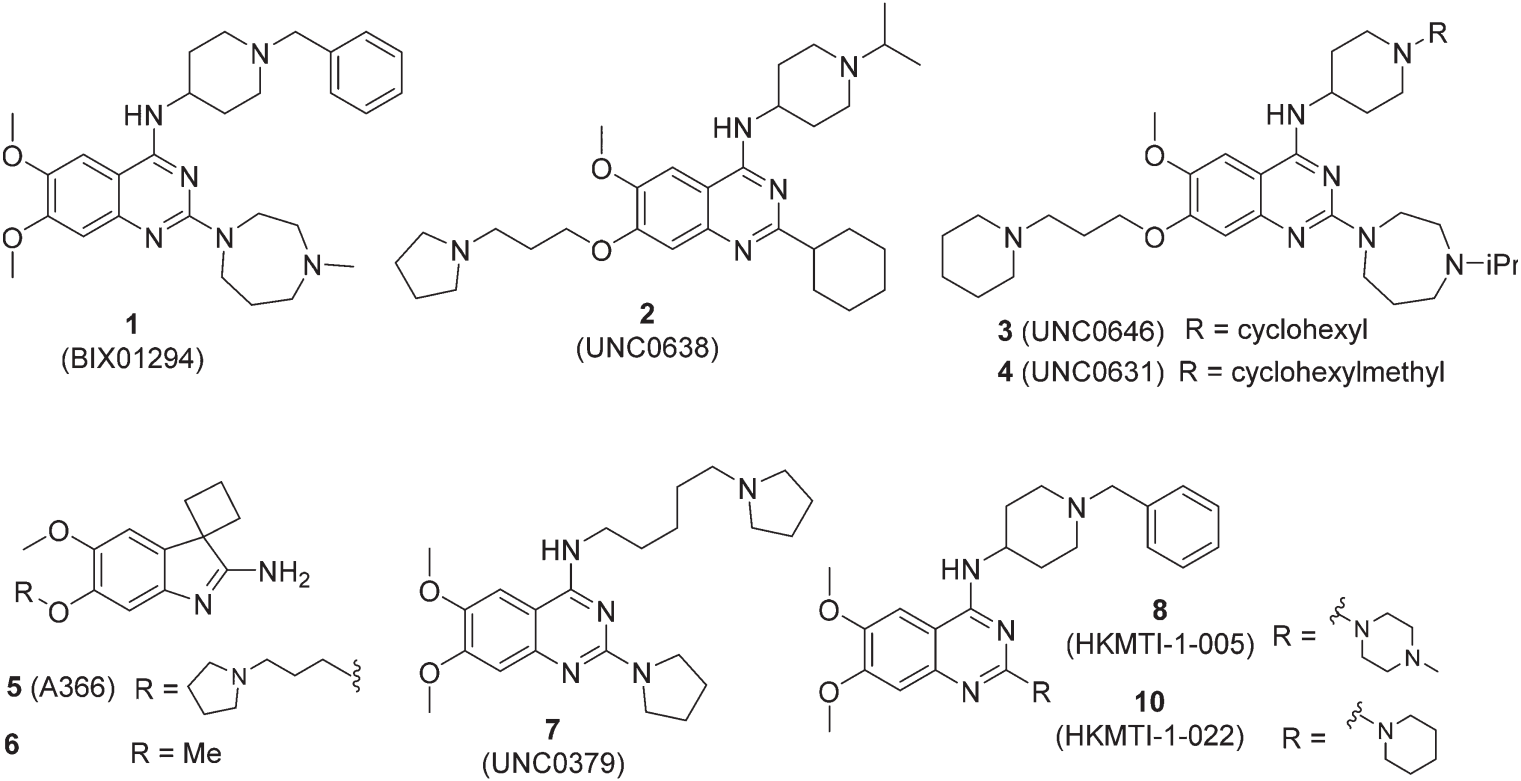
Representative examples of G9a (**1–6**), SET8 (**7**) and dual G9a/EZH2 (**8**, **10**) inhibitors.

We have recently reported our initial attempts to validate the PfHKMTs as a novel approach for antimalarial therapy.^31^–^34^ In the absence of the full complement of purified essential PfHKMTs – required for target-based hit discovery and SAR – we used a phenotypically-led approach; examining the activity of an established HKMT chemotype for antimalarial activity. Specifically, a focused library of inhibitors exemplifying the diaminoquinazoline HKMT chemotype was explored. Diaminoquinazoline HKMT activity was initially identified through a high throughput screen, with BIX01294 (**1**, Fig. 1) identified as an inhibitor of human G9a.^35^ While a number of medicinal chemistry studies – most notably those of Jin and co-workers – have been reported that improve the activity, selectivity, cell permeability and *in vivo* activity of G9a probes derived from **1**,^36^–^41^ it is becoming increasingly apparent that the HKMT activity of this chemotype is not limited to G9a. Indeed, by modifying the amino side chains of this scaffold, diaminoquinazoline inhibitors have been reported exhibiting human SETD8^42^,^43^ and EZH2^26^ activity (Fig. 1). Given this broad HKMT activity, it would seem that ‘repurposing’ the diaminoquinazoline scaffold as inhibitors of the homologous PfHKMTs (for a comparison of the homology of select *P. falciparum* SET domains to human proteins, see Table S1‡ in Malmquist *et al.*^31^) is a valid approach to progress these exciting new drug targets.

In our initial studies, **1** (Fig. 1) and a related analogue TM2-115 (**60**, Table 3) were found to exhibit rapid and irreversible asexual cycle blood stage-independent cytotoxic activity at nM concentrations, comparable potency against resistant strains (including artemisinin) and clinical isolates of *P. falciparum* and *P. vivax*, and oral efficacy in *in vivo* mouse models of *P. berghei* and *P. falciparum* infection.^31^,^32^ Highly promising effects were also observed for other life cycle stages, with mature gametocyte progression to gamete formation inhibited at submicromolar concentrations,^32^ and the unprecedented ability to reactivate dormant liver stages (hypnozoites) in a novel *in vitro* model system.^34^ A dose-dependent reduction in histone methylation (H3K4 and, to a lesser extent H3K9) was observed in parasites upon treatment (Western analysis), suggesting on-target PfHKMT activity,^31^ and that the broad ranging effects of these compounds is likely due to their target. A preliminary SAR study on our diaminoquinazoline series revealed that some pharmacophoric features might be conserved for both parasite-killing and G9a inhibition, thereby suggesting potential similarities between G9a and the yet unidentified PfHKMT target(s) responsible for the anti-parasitic activity.^33^ However, future development of this series will need to address host *versus* parasite selectivity; where inhibitory activity against human G9a is removed from the lead compounds, while maintaining potent anti-*Plasmodium* activity. Hence, we set out to identify regions around the scaffold that can be fine-tuned to improve the parasite-killing to G9a inhibition ratio. Herein, we report an extensive study of the SAR of this series against both G9a and *P. falciparum*. To provide a more complete picture of the underlying SAR, some of the analogues included (mainly in the ESI‡), and their anti-*Plasmodium* activities, were previously reported by us.^33^ However, G9a inhibition for such analogues is reported here for the first time. Such cases are clearly marked in Tables 1–4. Important and previously unidentified trends are determined for activity against both targets and, critically, we elucidate features of this scaffold that allow for high parasite *versus* host selectivity. We believe this study further cements the potential of this scaffold as a candidate for development into greatly needed novel therapies to control malaria.

**Table 1:** SAR at position 2

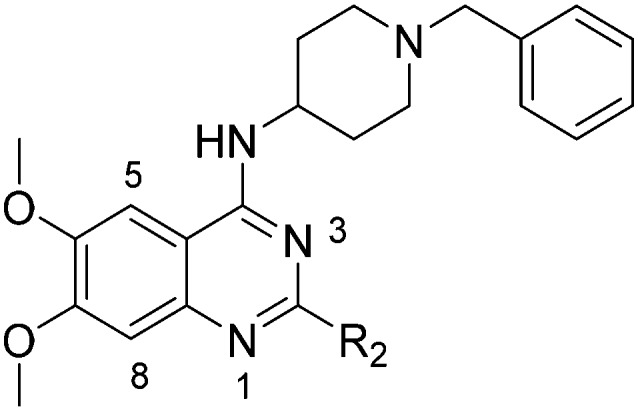								
	R_2_	Pf3D7 IC_50_ (nM)^*^	G9a IC_50_ (nM)^*^	G9a/Pf3D7	HepG2 IC_50_ (nM)	HepG2/Pf3D7	clog *P*	TPSA
**1** BIX01294	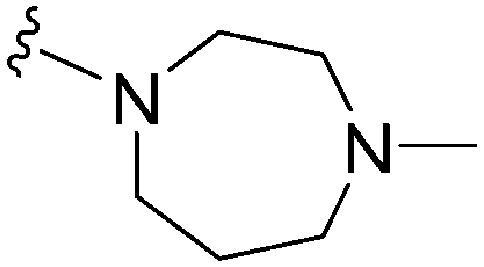	43^*a*^	67 (110^*b*^/290^*c*^)	1.6	4800	111.6	3.86	65.99
**8**	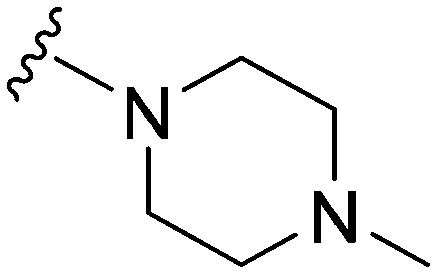	18^*a*^	101	5.6	5500	305.5	3.48	65.99
**9**	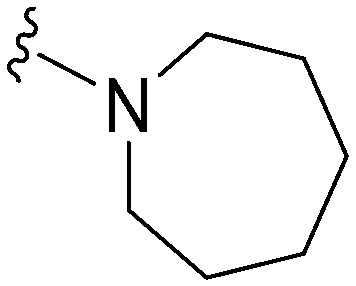	29^*a*^	332	11.4	3800	131	5.10	62.75
**10**	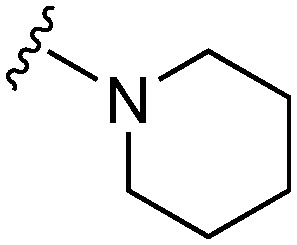	23^*a*^	472	20.5	4700	201.7	4.71	62.75
**11**	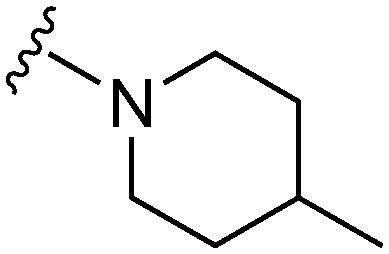	76^*a*^	1326	17.4	5400	71.0	4.96	62.75
**12**	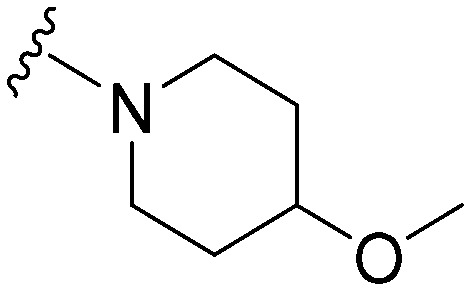	38^*a*^	576	15.2	10 100	265.8	4.34	71.98
**13**	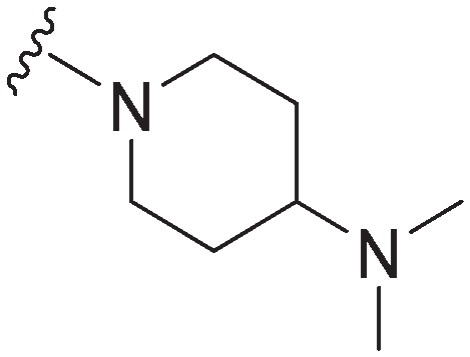	37^*a*^	506	13.7	5900	159.5	4.25	65.99
**14**	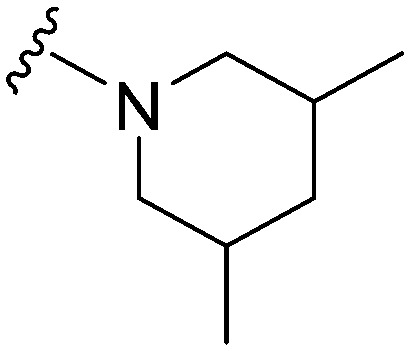	67^*a*^	∼10 000	∼149.3	3600	53.7	5.21	62.75
**15**	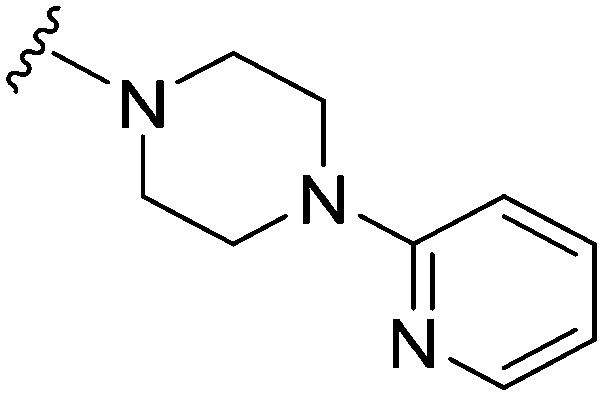	26^*a*^	3190	122.7	2600	100	4.44	78.88
**16**	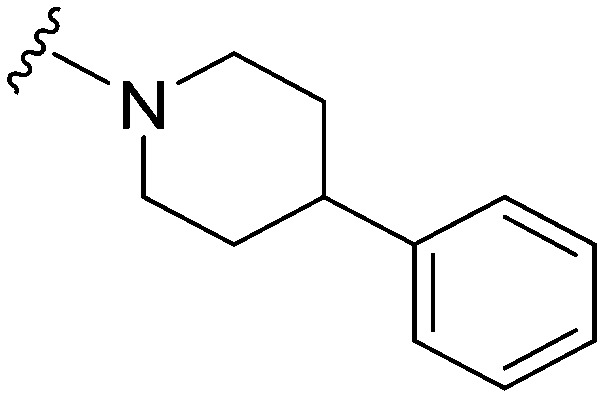	72^*a*^	>10 000	>138.9	6100	84.7	6.11	62.75
**17**	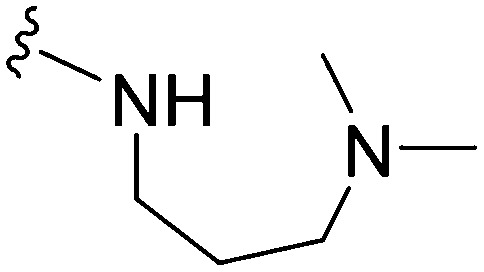	>1000	123	<0.1	ND	ND	4.09	74.78
**Pyrimethamine**		33	ND	ND	ND	ND		
**Chloroquine**		8	ND	ND	ND	ND		

^*a*^Parasite-killing activity reported earlier.^33^

^*b*^IC_50_ reported using enzyme-coupled SAH detection (ECSD) assay.^36^,^37^

^*c*^IC_50_ reported using chemiluminescence-based oxygen tunnelling (CLOT) assay.^36^,^37^

## Chemistry

**Scheme 1:**
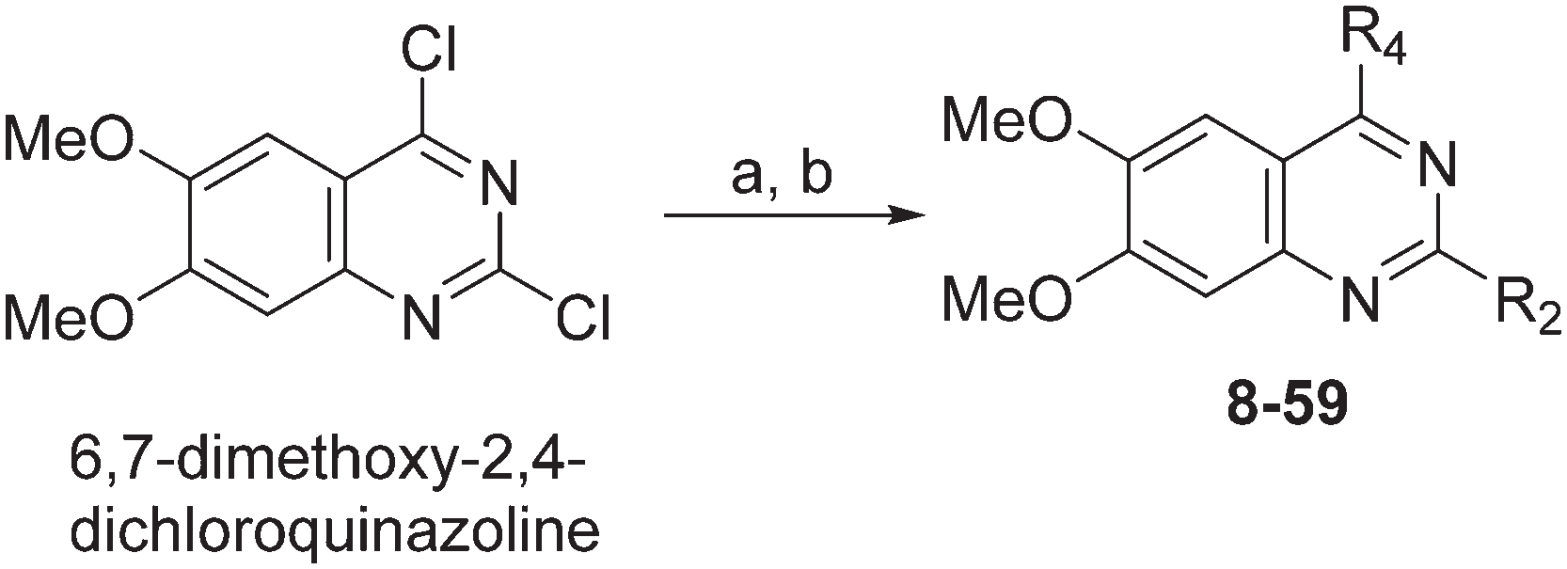
Synthesis of diaminoquinazolines in Tables 1 and 2. Reagents and conditions: (a) various amines, Et_3_N (or DIEA), THF (or DMF), RT, 18–24 h; (b) various amines (5–10 equiv.), microwave, toluene (or neat), 130–185 °C, 30–50 min or i-PrOH, 4 M HCl/dioxane, microwave, 160 °C, 15 min.

Molecules **8–59** (Tables 1 and 2) were synthesized following a well-established two-step synthetic procedure (Scheme 1).^36^–^38^,^41^ Hence, 6,7-dimethoxy-2,4-dichloroquinazoline was treated with various commercially available or synthesized (see ESI‡) *N*-substituted-4-piperidylamines, in order to obtain 4-substituted quinazoline derivatives. These intermediates were subsequently heated with a second amine nucleophile to access the desired diaminoquinazolines analogues.

**Table 2:** SAR at position 4

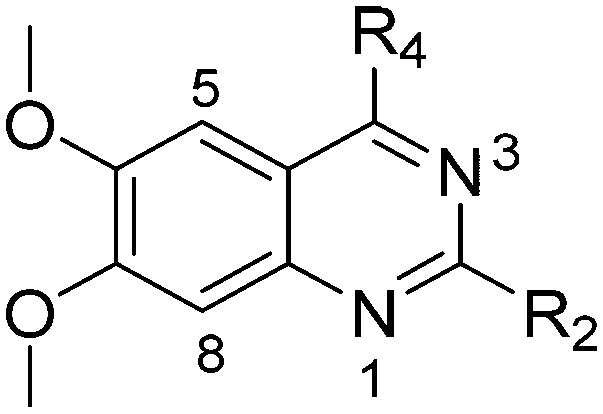									
ID	R_4_	R_2_	Pf3D7 IC_50_ (nM)^*^	G9a IC_50_ (nM)^*^	G9a/Pf3D7	HepG2 IC_50_ (nM)	HepG2/Pf3D7	clog *P*	TPSA
**18**	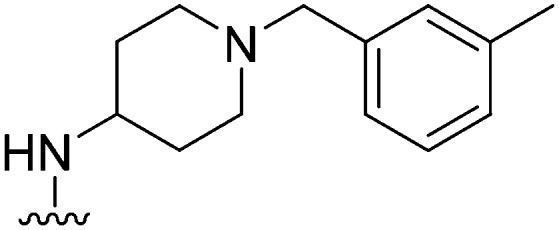	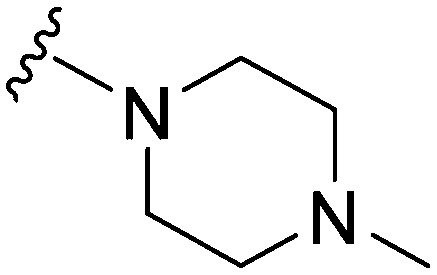	74	116	1.6	3100	41.9	3.78	65.99
**19**	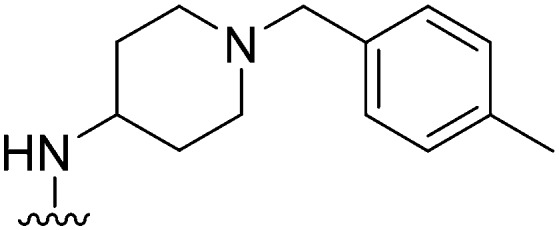	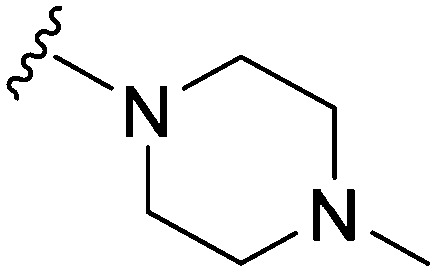	69	78	1.1	3300	47.8	3.78	65.99
**20**	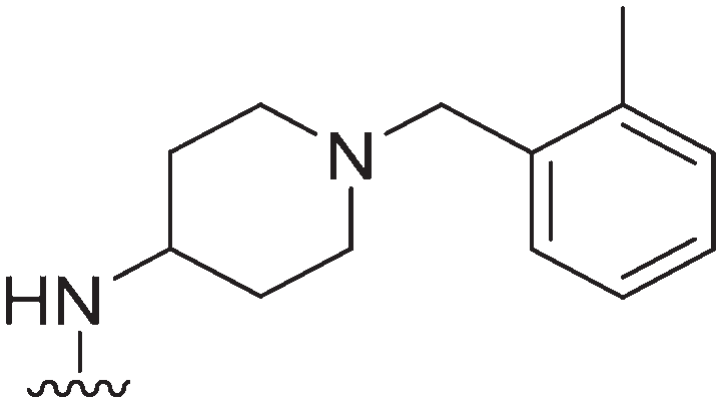	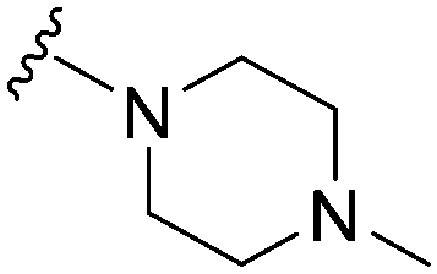	80	169	2.1	4100	51.2	3.78	65.99
**21**	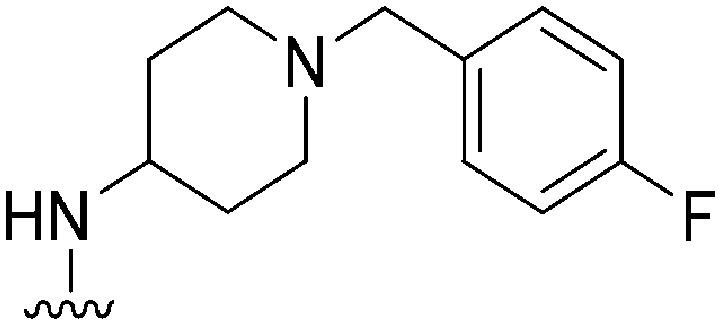	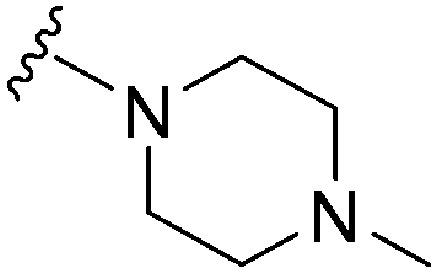	NT	112	—	ND	ND	3.61	65.99
**22**	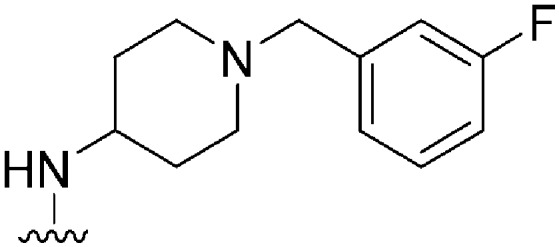	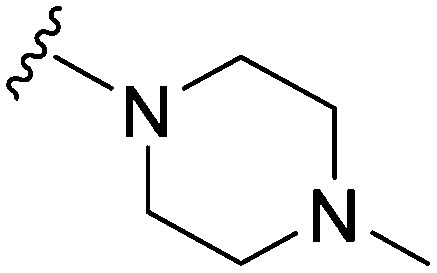	148	132	0.9	ND	ND	3.61	65.99
**23**	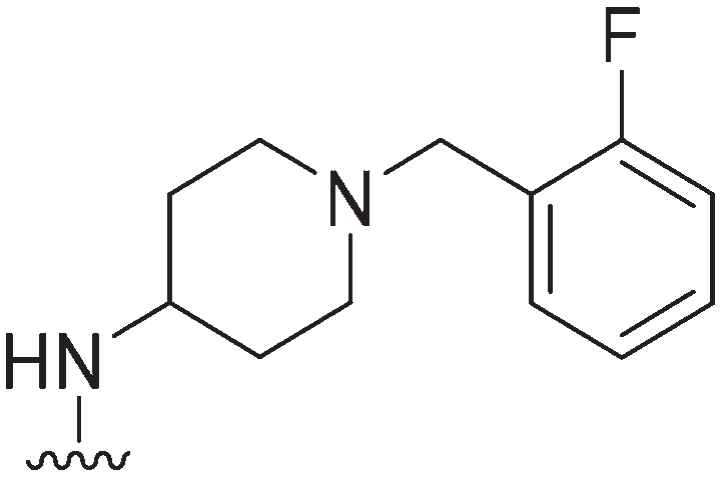	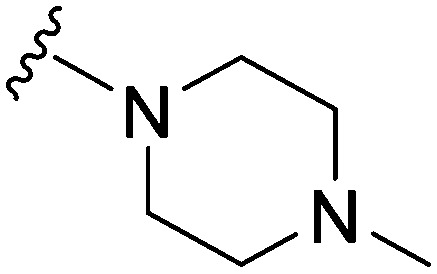	137	116	0.8	ND	ND	3.61	65.99
**24**	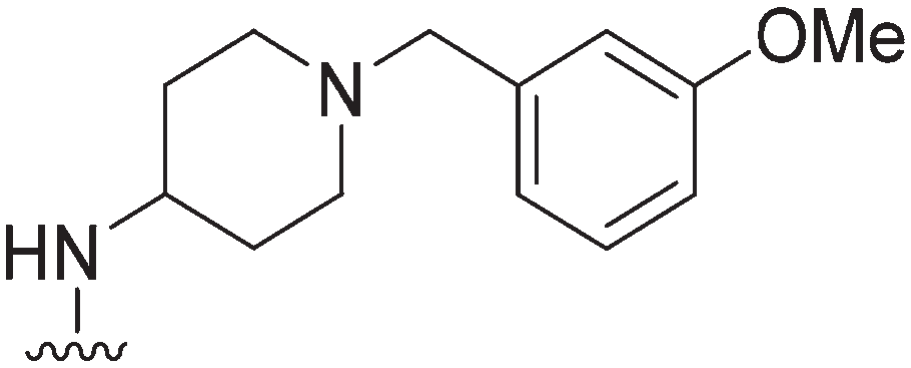	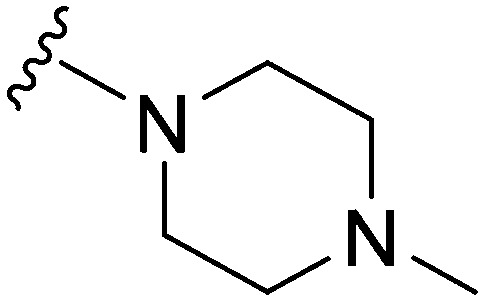	237	128	0.5	ND	ND	3.48	75.22
**25**	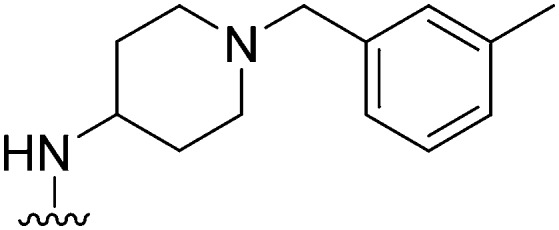	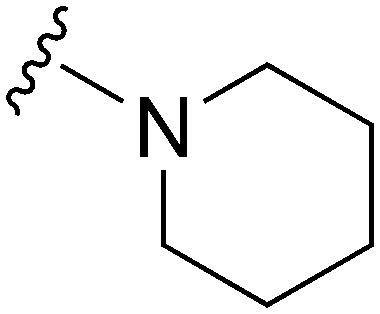	77	869	11.3	2800	36.4	5.02	62.75
**26**	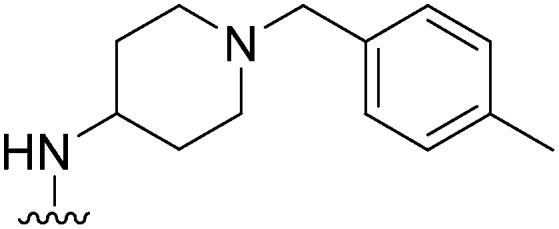	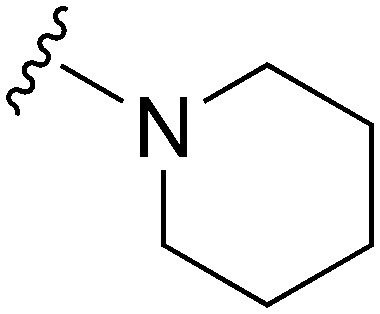	77	690	9.0	2700	35.1	5.02	62.75
**27**	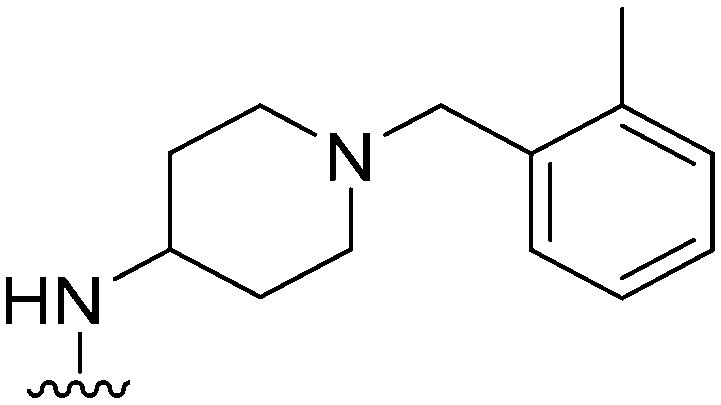	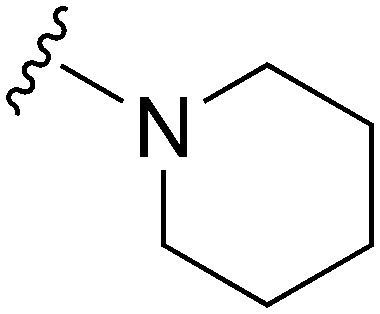	78	∼1000	∼12.8	2900	37.2	5.02	62.75
**28**	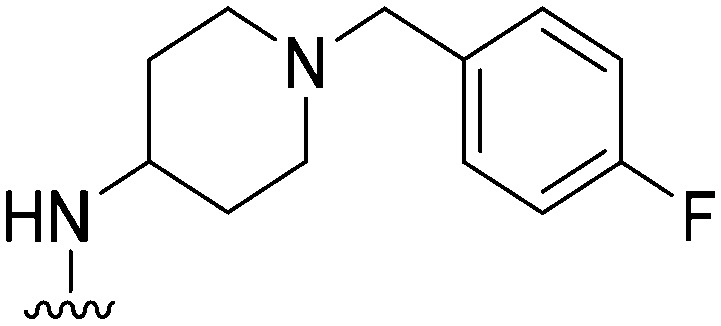	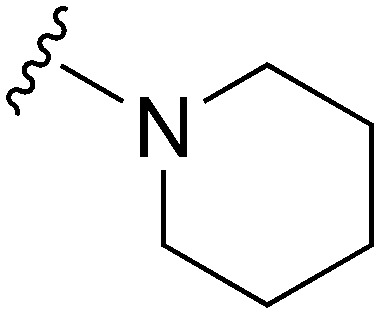	191	1214	6.4	ND	ND	4.85	62.75
**29**	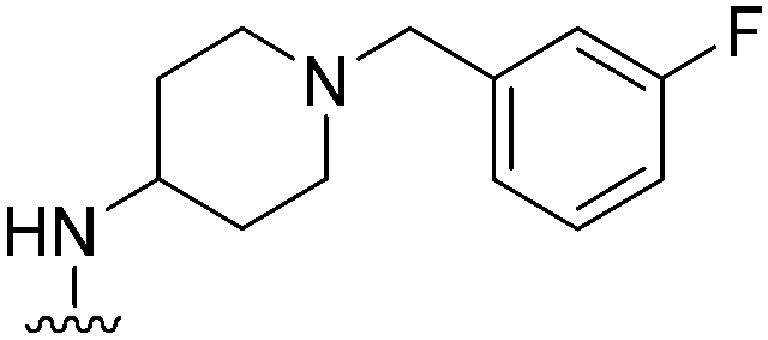	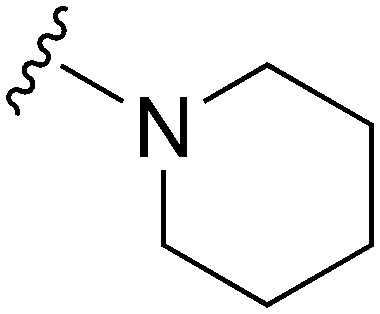	NT	∼1000	—	ND	ND	4.85	62.75
**30**	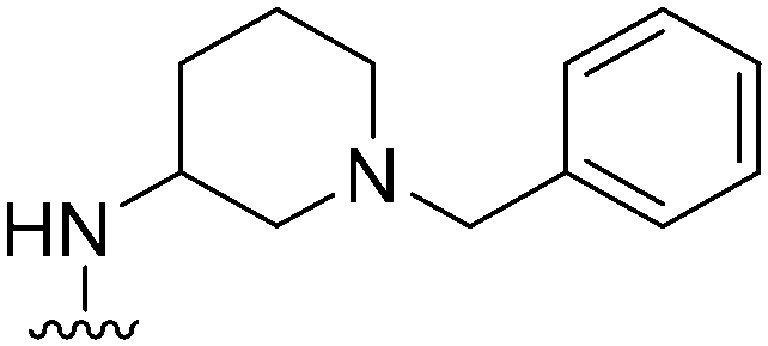	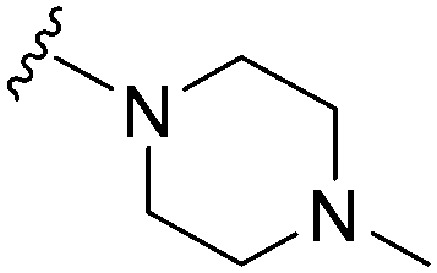	197^*a*^	∼1000	∼5.1	10 500	53.3	3.48	65.99
**31**	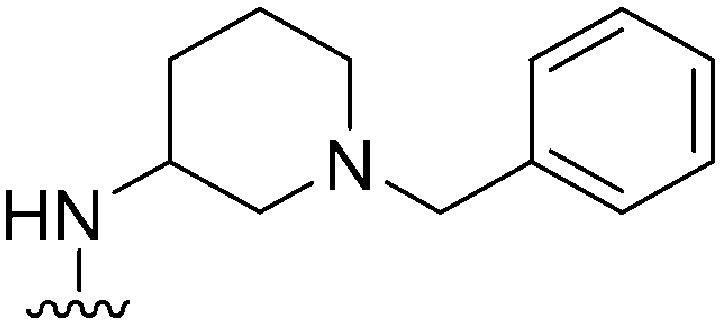	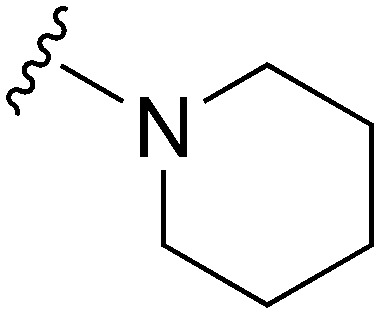	178^*a*^	∼1000	∼5.6	6300	35.4	4.71	62.75
**32**	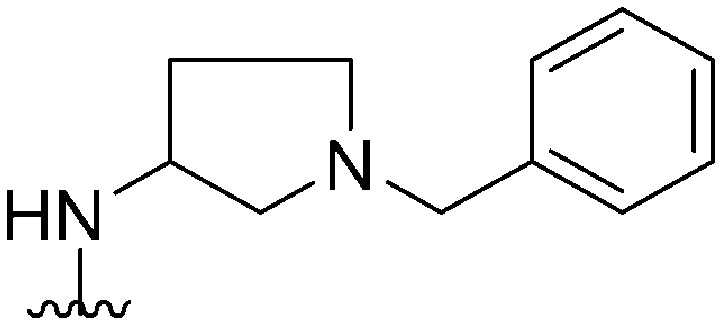	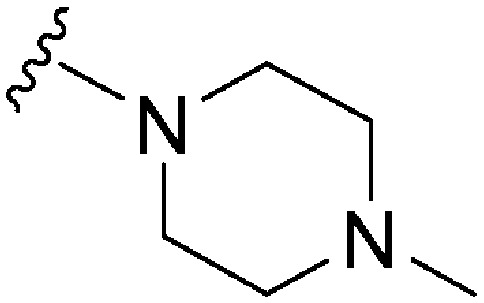	330^*a*^	344	1.0	ND	ND	3.09	65.99
**33**	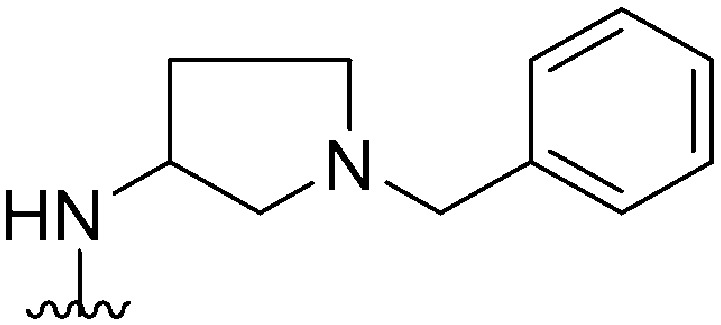	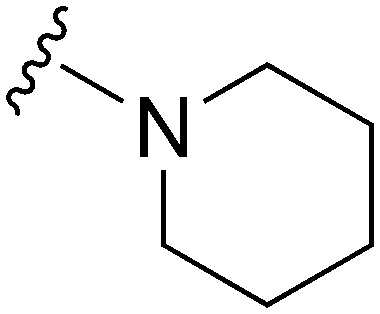	94^*a*^	830	8.8	6300	67	4.32	62.75
**34**	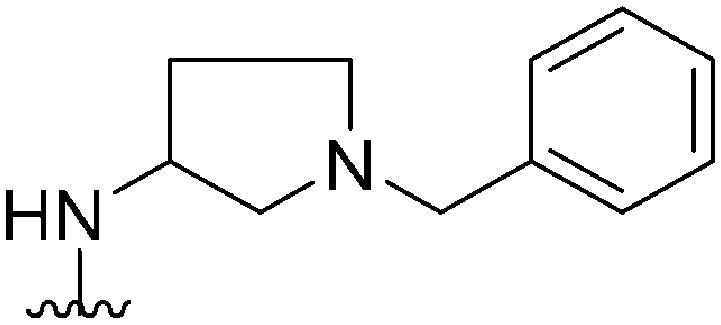	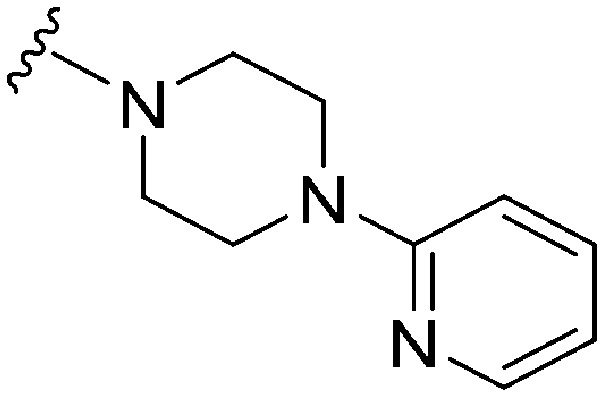	93^*a*^	∼10 000	∼107.5	5600	60.2	4.06	78.88
**35**	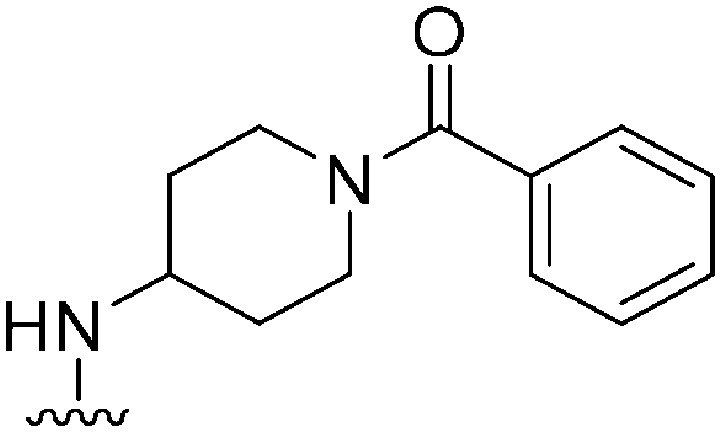	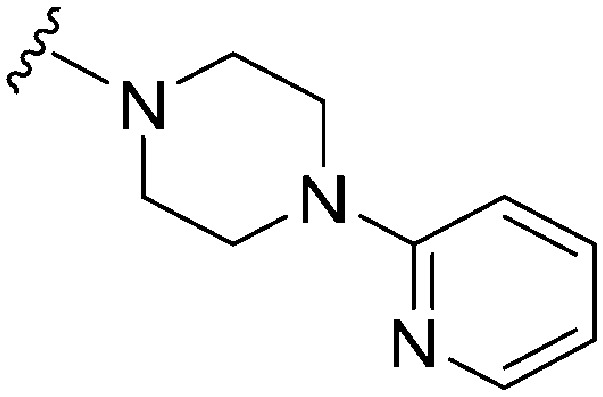	247^*a*^	>10 000	>40.5	ND	ND	4.08	95.95
**36**	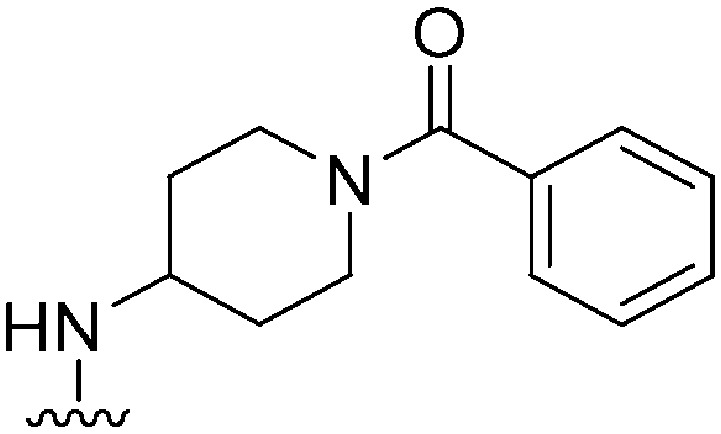	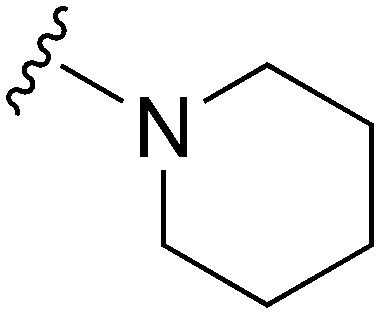	369^*a*^	>10 000	>27.1	ND	ND	4.35	79.82
**37**	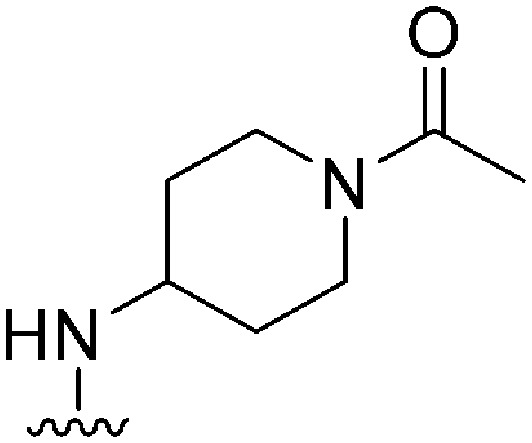	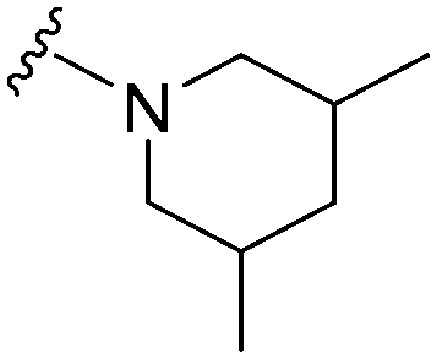	107^*a*^	>1000	>9.3	17 800	166.3	3.55	79.82
**38**	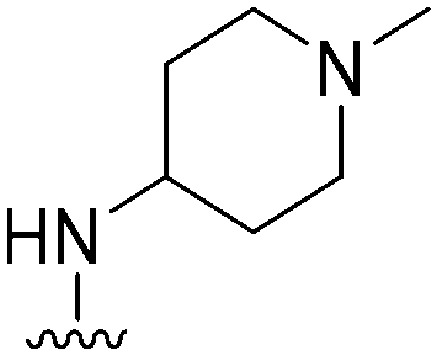	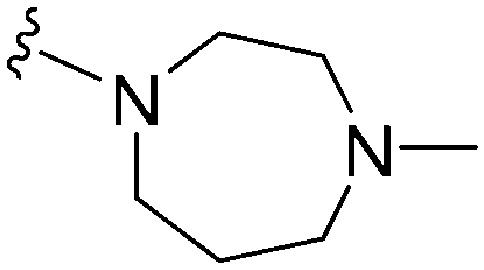	>300^*a*^	330^*b*^/230^*c*^	—	ND	ND	2.30	65.99
**39**	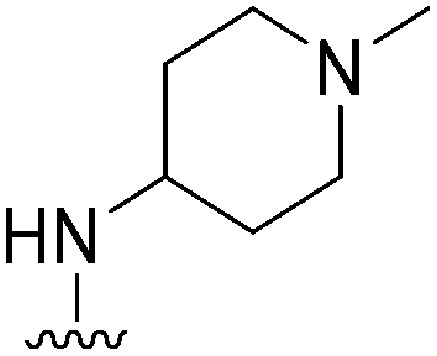	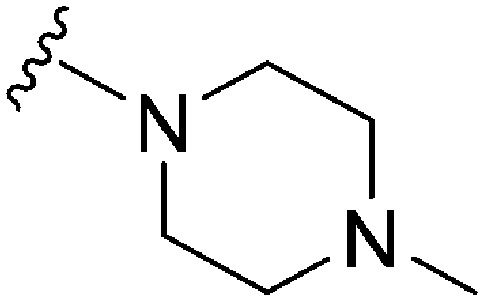	>300^*a*^	680^*b*^/200^*c*^	—	ND	ND	1.90	65.99
**40**	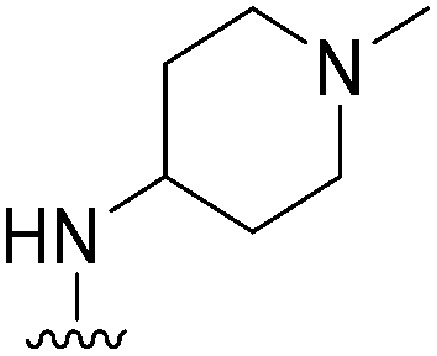	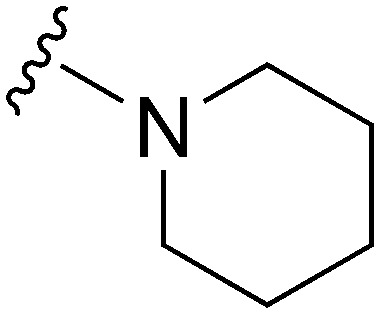	37^*a*^	591	16.0	14 200	383.8	3.14	62.75
**41**	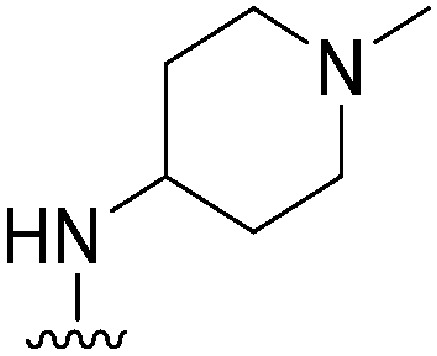	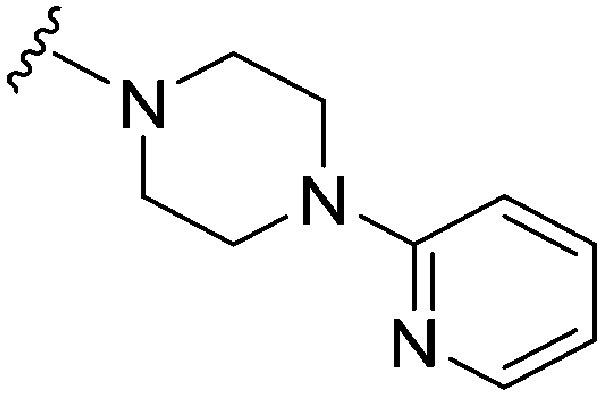	56^*a*^	>1000	>17.9	5500	98.2	2.88	78.88
**42**	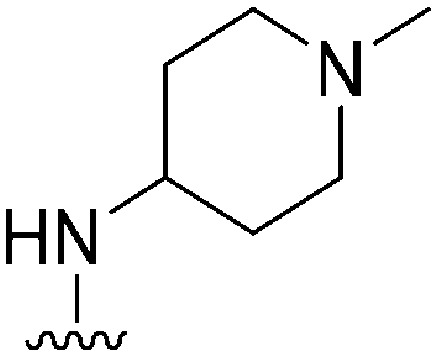	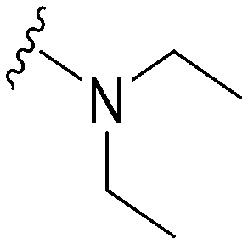	28^*a*^	910^*b*^/6500^*c*^	—	>10 000	>357	3.00	62.75
**43**	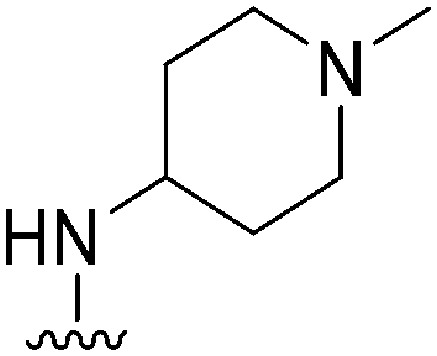	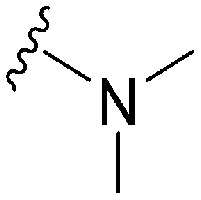	34^*a*^	1100^*b*^/900^*c*^	—	>10 000	>294	2.22	62.75
**44**	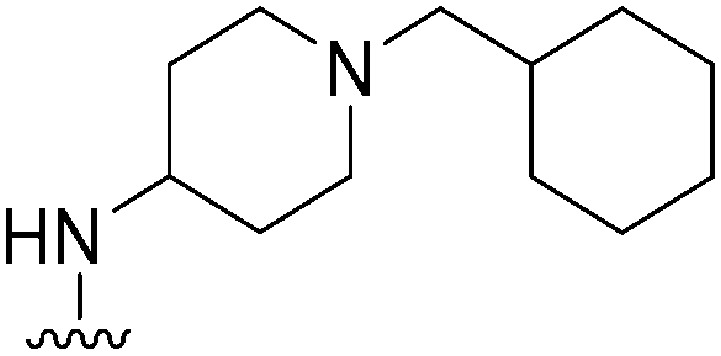	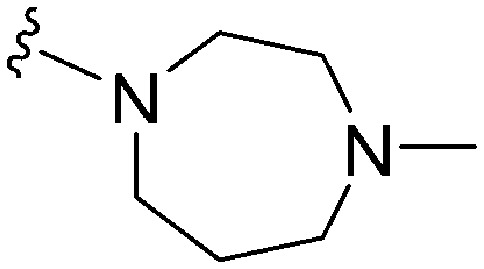	174	10	0.06	ND	ND	4.25	65.99
**45**	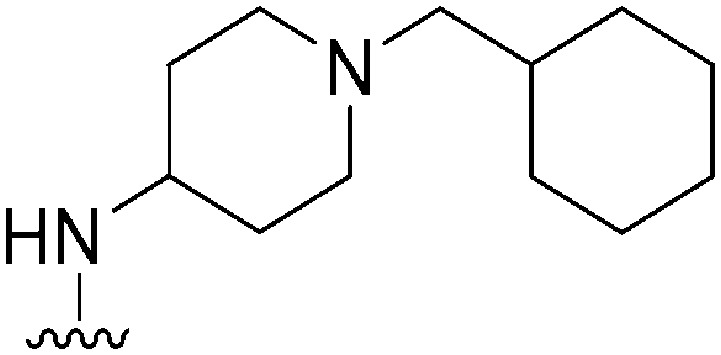	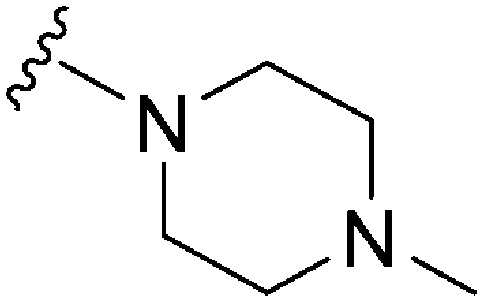	130	25	0.19	ND	ND	3.86	65.99
**46**	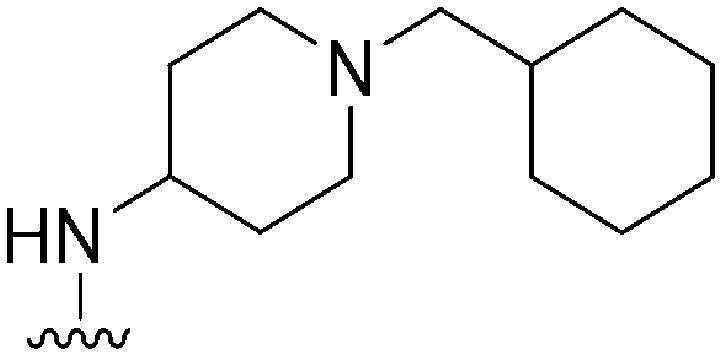	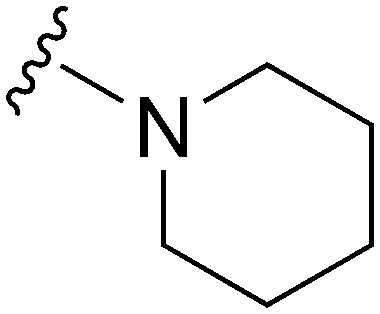	144	295	2.0	ND	ND	5.09	62.75
**47**	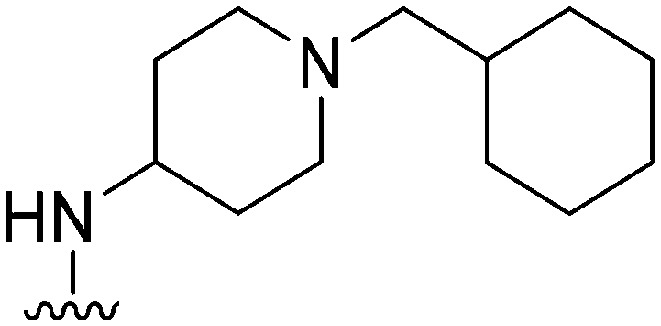	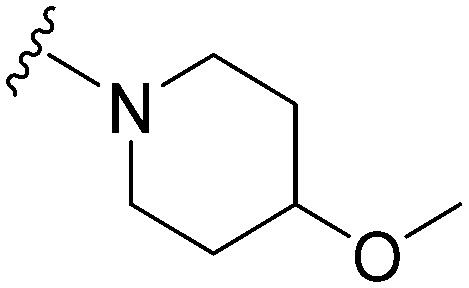	47	185	3.9	ND	ND	4.72	71.98
**48**	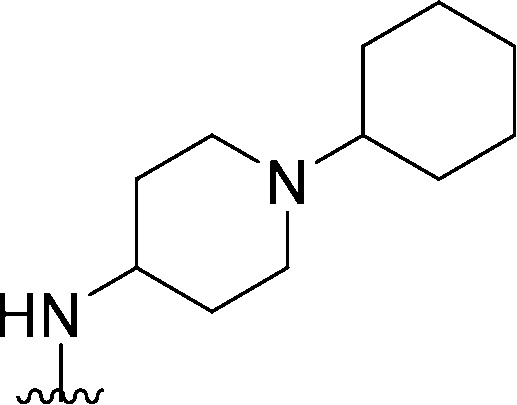	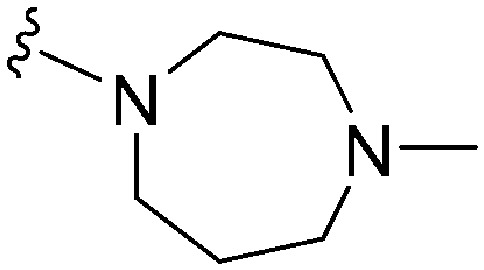	205	55	0.27	ND	ND	4.00	65.99
**49**	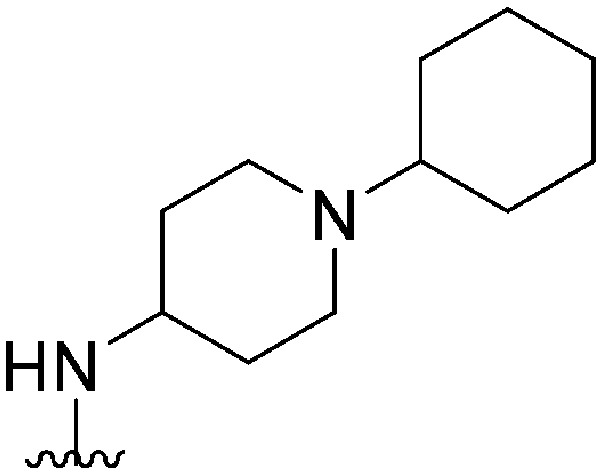	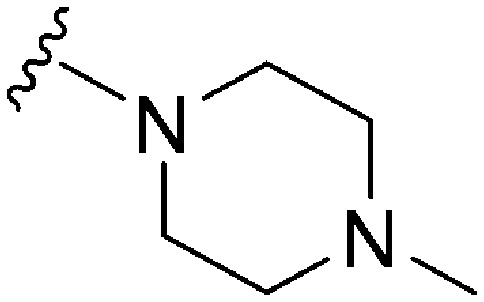	222	49	0.22	ND	ND	3.61	65.99
**50**	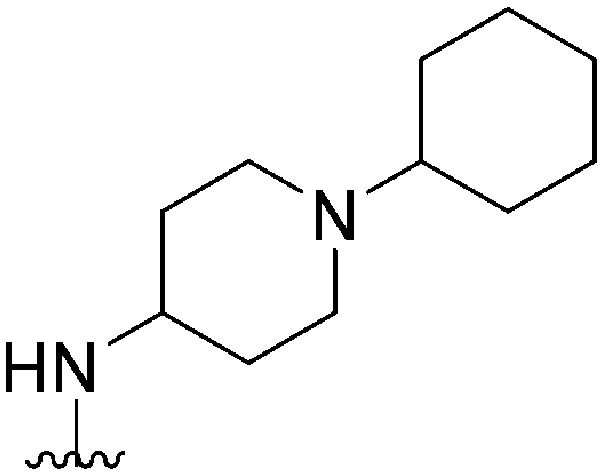	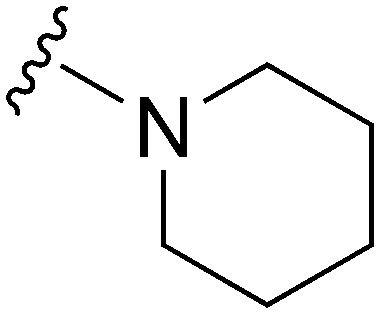	108	NT	—	ND	ND	4.85	62.75
**51**	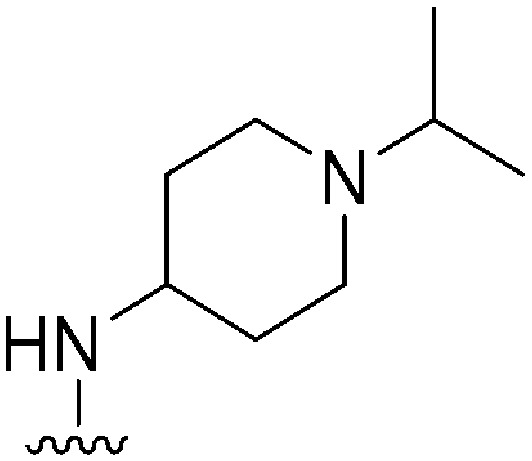	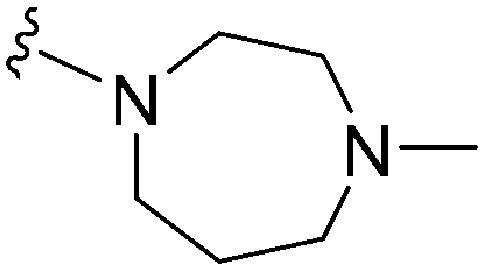	470	55	0.12	ND	ND	3.07	65.99
**52**	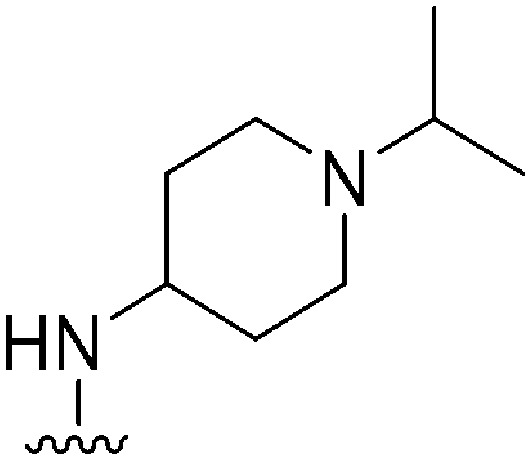	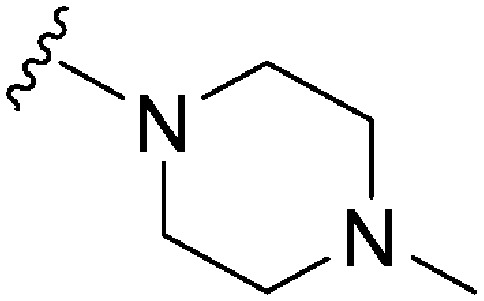	280	123	0.44	ND	ND	2.68	65.99
**53**	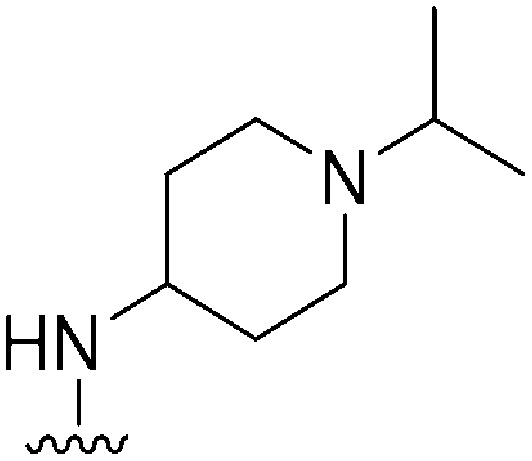	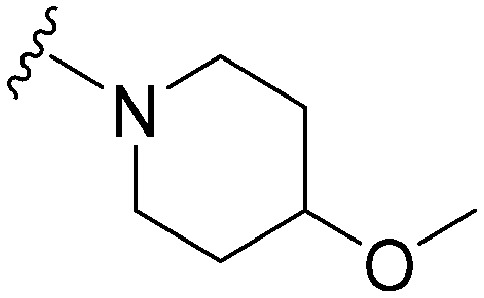	126	NT	—	ND	ND	3.55	71.98
**54**	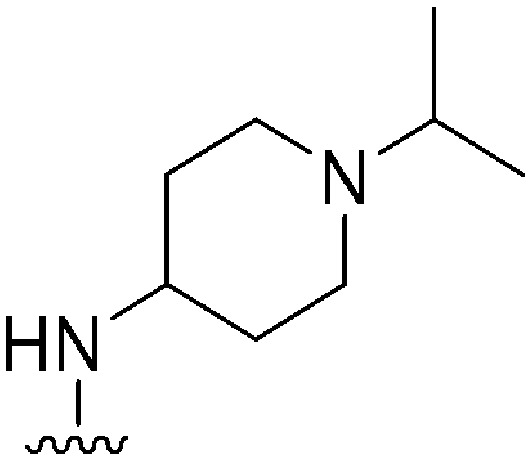	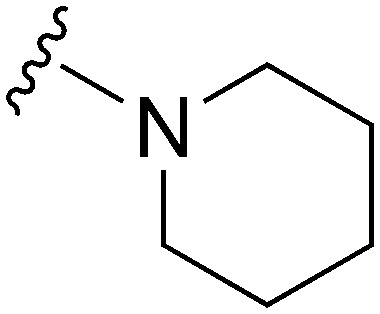	71	319	4.5	ND	ND	3.92	62.75
**55**	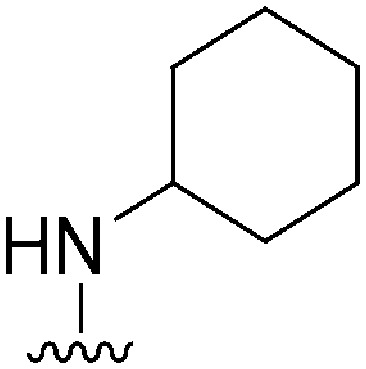	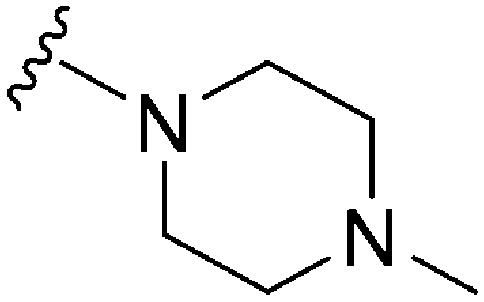	215	6438	29.9	ND	ND	3.14	62.75
**56**	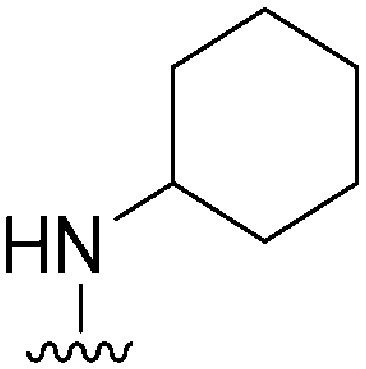	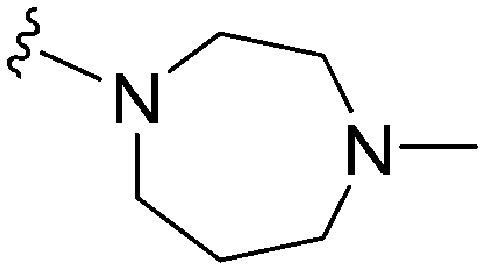	107	3877	36.2	ND	ND	3.53	62.75
**57**	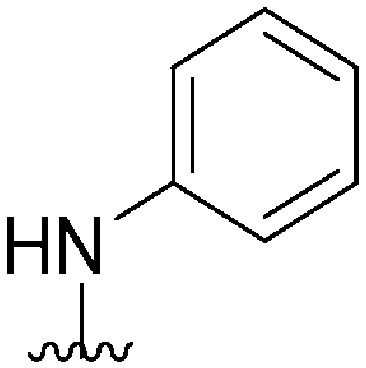	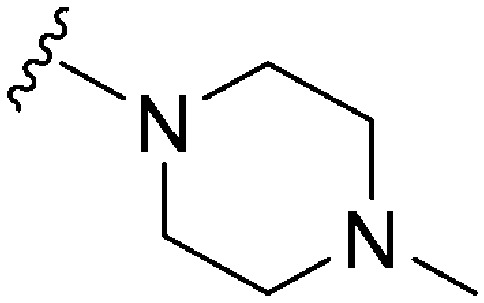	570^*a*^	>10 000	>17.5	ND	ND	3.14	62.75
**58**	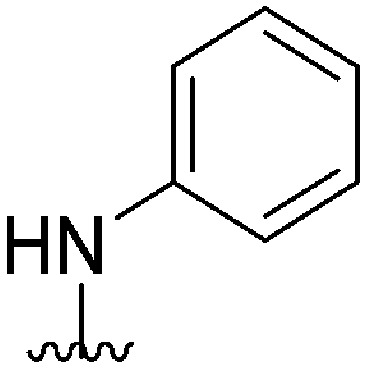	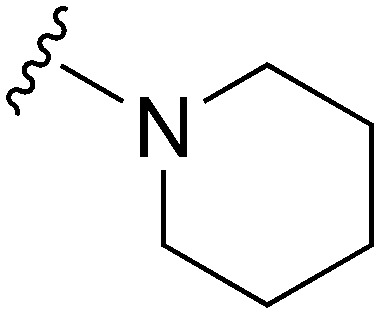	161^*a*^	>10 000	>62.1	ND	ND	4.38	59.51
**59**	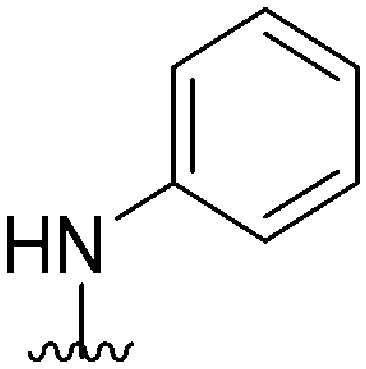	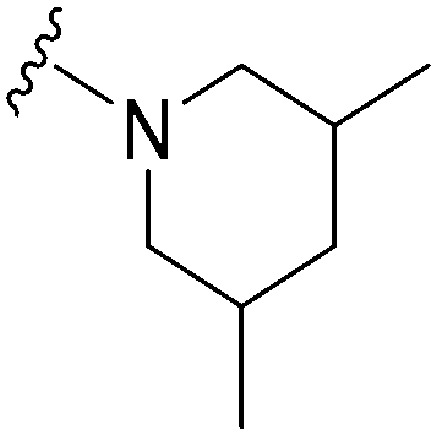	77^*a*^	>10 000	>129.9	12 200	158.4	4.87	59.51

^*a*^Parasite-killing activity reported earlier.^33^

^*b*^IC_50_ reported using enzyme-coupled SAH detection (ECSD) assay.^36^,^37^

^*c*^IC_50_ reported using chemiluminescence-based oxygen tunnelling (CLOT) assay.^36^,^37^

**Scheme 2:**
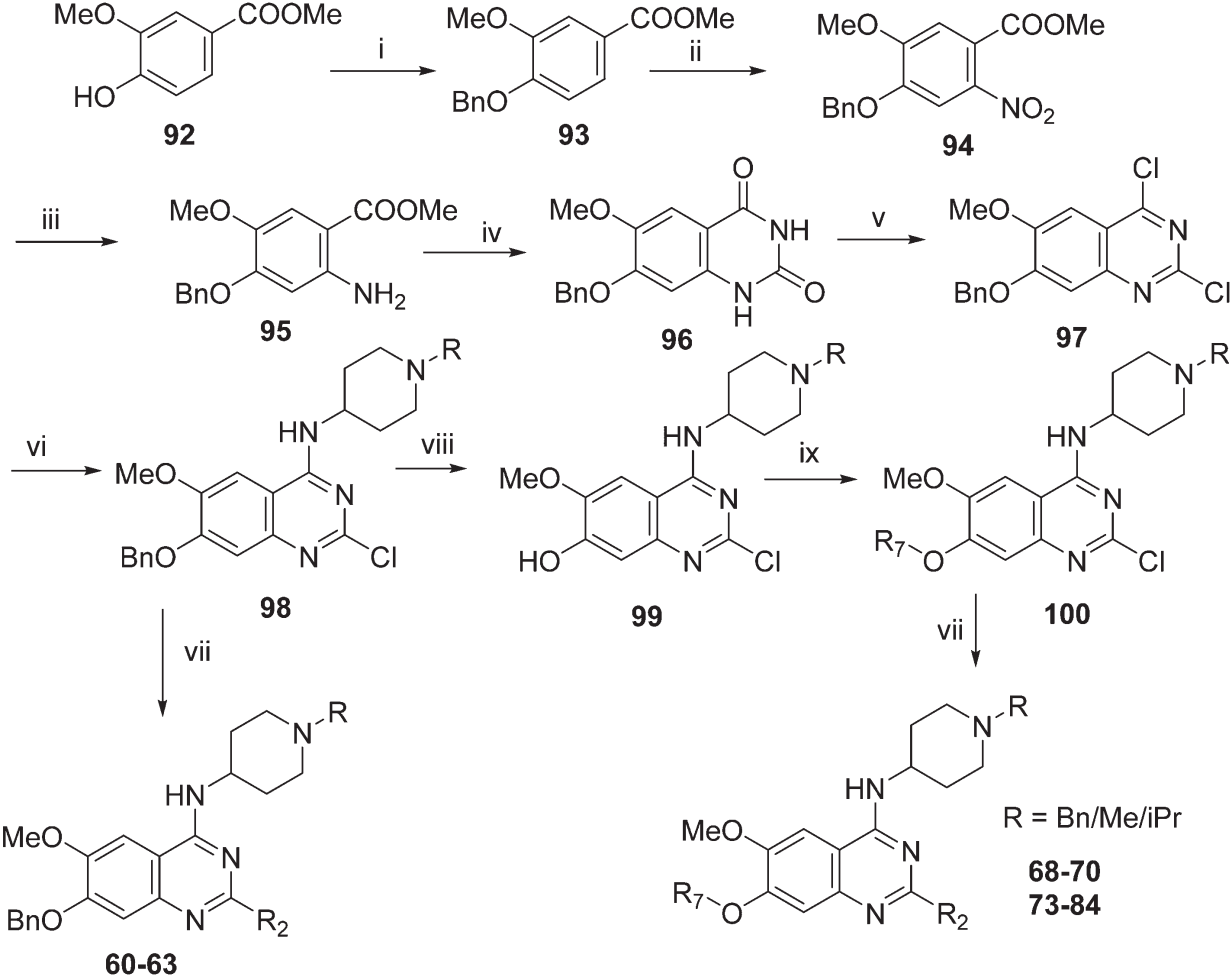
Synthesis of diaminoquinazolines in Table 3. Reagents and conditions: (i) benzyl bromide, K_2_CO_3_, DMF, 0 °C – RT, 18 h; (ii) HNO_3_, Ac_2_O, RT, 18 h; (iii) Fe, NH_4_Cl, i-PrOH/H_2_O, reflux, 18 h; (iv) a) NaOCN, H_2_O/AcOH, RT, 18 h; b) NaOH, MeOH, reflux, 6 h; (v) POCl_3_, DIEA, acetonitrile, reflux, 6 h; (vi) various amines, Et_3_N (or DIEA), THF (or DMF), RT, 18–24 h; (vii) various amines (5–10 equiv.), microwave, toluene (or neat), 130–185 °C, 30–50 min or i-PrOH, 4 M HCl/dioxane, microwave, 160 °C, 15 min; (viii) TFA, reflux, 3 h; (ix) K_2_CO_3_ (or Cs_2_CO_3_), DMF, alkyl halides, 3–18 h 80 °C or PPh_3_, DIAD, THF, 20 h, RT.

Analogues with benzyl or alkoxy substituents at position 7 (Table 3) were synthesized following a previously described methodology,^36^–^38^ with slight modifications, as shown in Scheme 2. The phenolic oxygen of the commercially available 4-hydroxy-3-methoxybenzoate (**92**) was first benzylated to give **93**. Nitration of **93** gave nitro compound **94** which was further reduced to obtain aniline **95**. Conversion of **95** into a urea intermediate, followed by base-mediated ring closure yielded quinazolinedione **96** that was subsequently heated with phosphorous oxychloride to obtain the 2,4-dichloroquinazoline derivative **97**. Subsequent displacement of the chloride atoms from position 4 and 2, analogously to that described in Scheme 1, yielded final compounds **60–63** possessing 7-OBn substituents. Alternatively, **98** was debenzylated under acidic conditions to yield intermediates **99** possessing a free phenol group at position 7. The phenol oxygen was either alkylated using a selection of alkyl halides or else treated with primary alcohols under Mitsunobu conditions to give substituted 2-chloroquinazolines **100**. Finally, substitution of the chloride at position 2 of **100** with a second amine yielded the desired analogues **68–70** and **73–84**. The synthesis of analogues **64–67**, **71**, **72** (Table 3) has been reported earlier by Jin *et al.* using similar synthetic scheme.^36^–^38^

**Table 3:** SAR at position 7

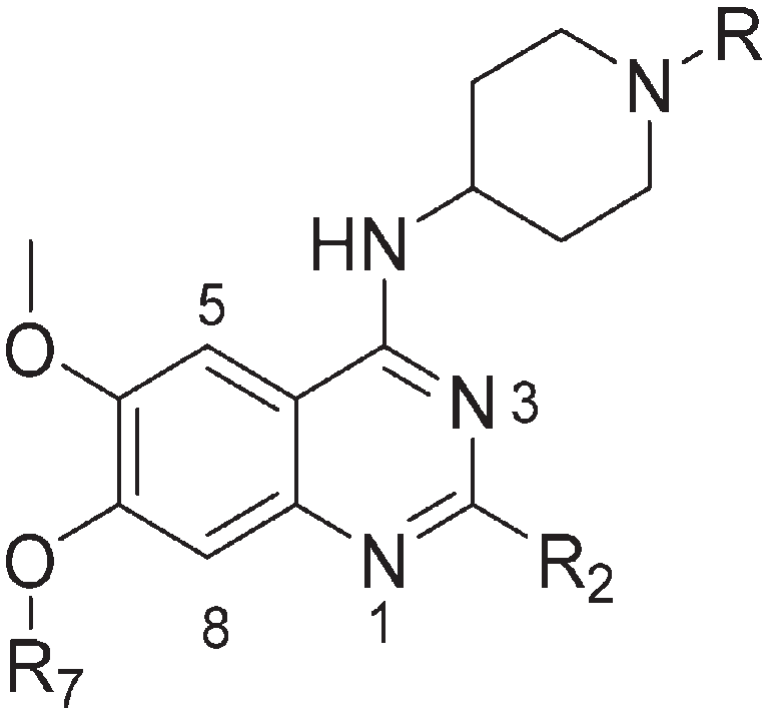										
	R	R_7_	R_2_	Pf3D7 IC_50_ (nM)^*^	G9a IC_50_ (nM)^*^	G9a/Pf3D7	HepG2 IC_50_ (nM)	HepG2/Pf3D7	clog *P*	TPSA
**60** TM2-115	–Me	–Bn	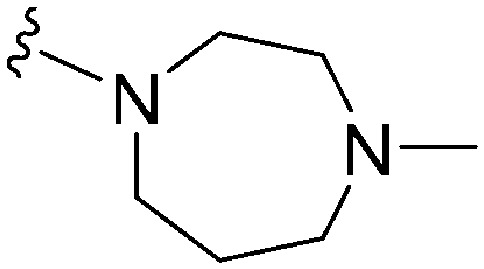	43^*a*^	>1000	23.2	4700	110.1	3.86	65.99
**61**	–Bn	–Bn	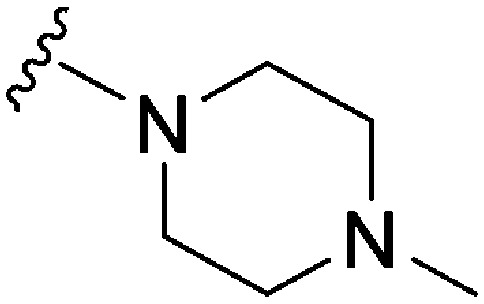	82	∼10 000	122	2900	35.4	5.05	65.99
**62**	–Bn	–Bn	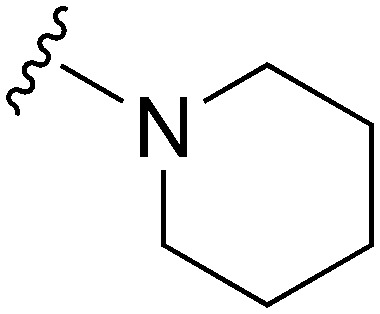	57	>50 000	877.2	2900	50.9	6.28	62.75
**63**	–Me	–Bn	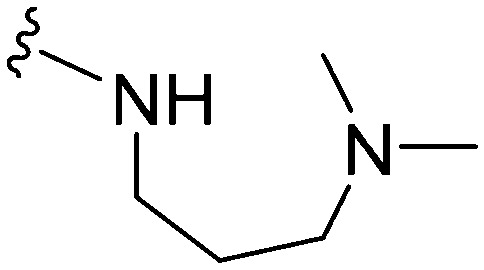	>1000	>1000	—	ND	ND	4.09	74.78
**64**	–Me	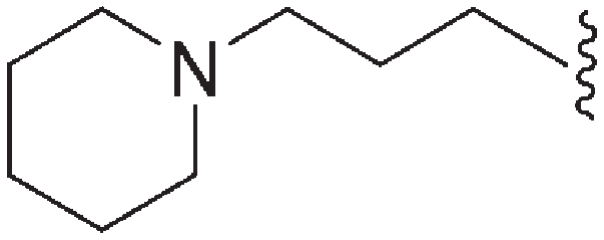	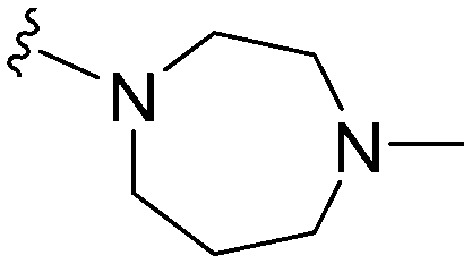	>2000^*a*^	25^*b*^/20^*c*^	—	ND	ND	3.54	69.23
**65**	–Me	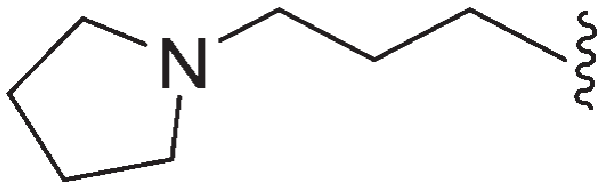	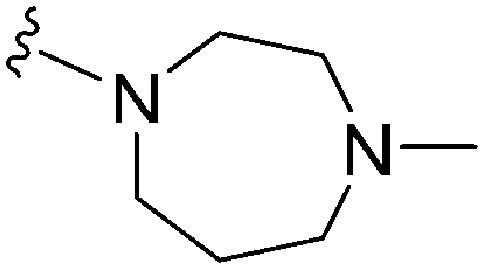	>2000^*a*^	8^*b*^	—	ND	ND	3.15	69.23
**66** (UNC0224)	–Me	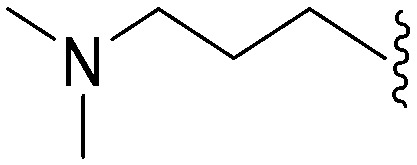	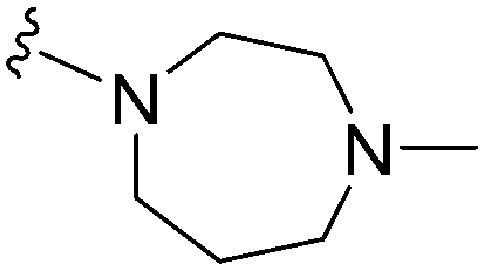	>2000^*a*^	43^*b*^/57^*c*^	—	ND	ND	2.62	69.23
**67**	–Me	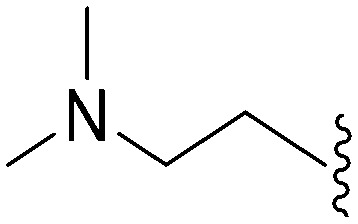	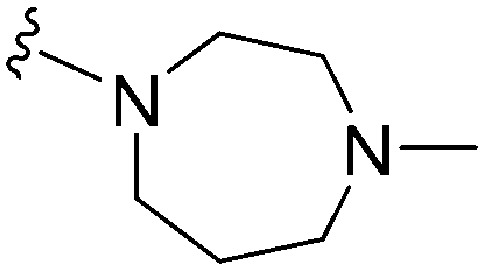	>2000^*a*^	110^*b*^/120^*c*^	—	ND	ND	2.23	69.23
**68**	–iPr	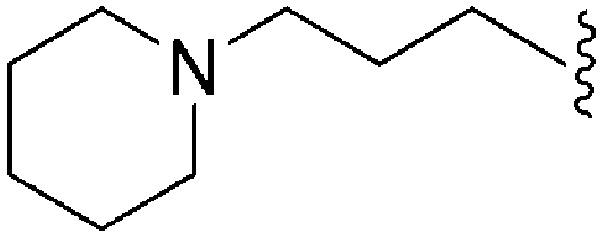	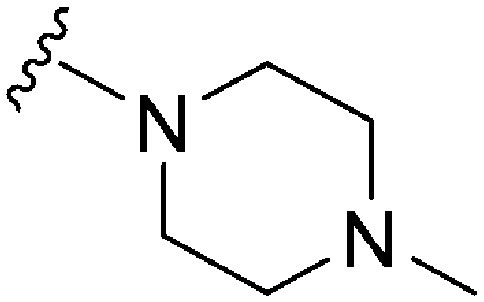	1120	4	0.004	ND	ND	3.93	69.23
**69**	–Bn	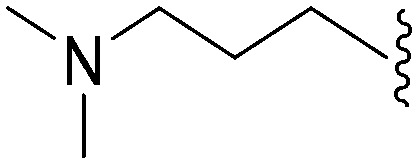	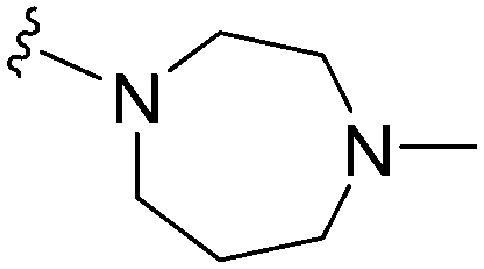	632	<3	>0.005	ND	ND	4.19	69.23
**70**	–Bn	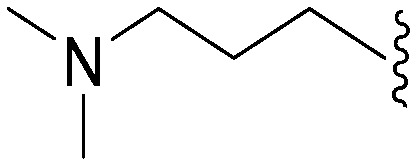	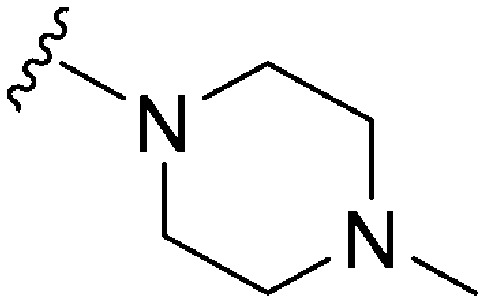	449	8	0.018	ND	ND	3.80	69.23
**71**	–Me	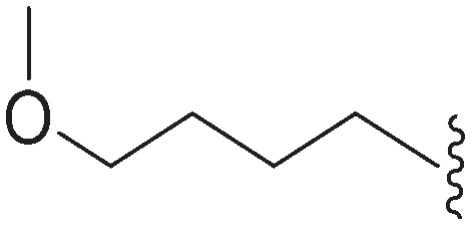	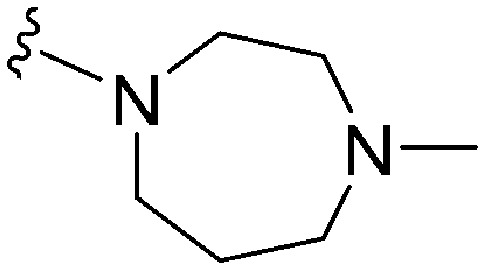	319^*a*^	>10 000^*c*^	—	ND	ND	3.09	75.22
**72**	–Me	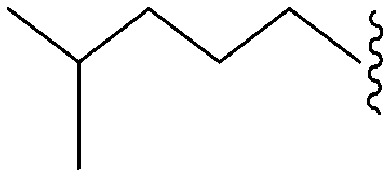	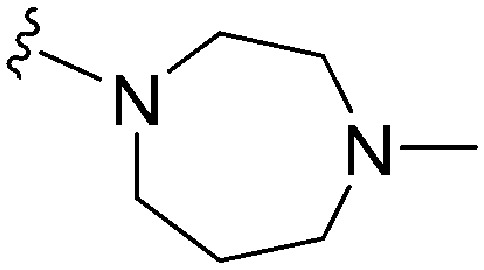	150^*a*^	3400^*b*^/5200^*c*^	—	ND	ND	4.10	65.99
**73**	–Bn	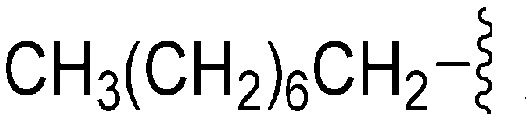	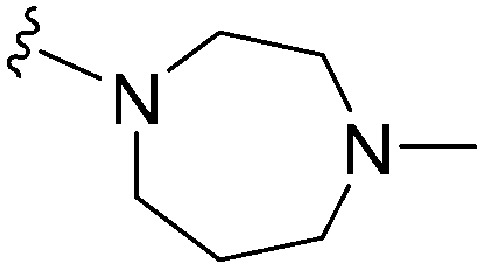	268	211	0.79	ND	ND	6.60	65.99
**74**	–Bn	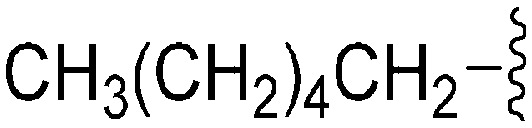	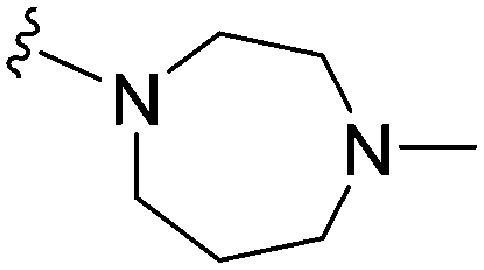	235	39	0.17	ND	ND	5.82	65.99
**75**	–Bn	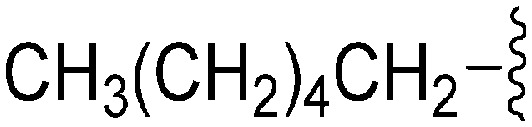	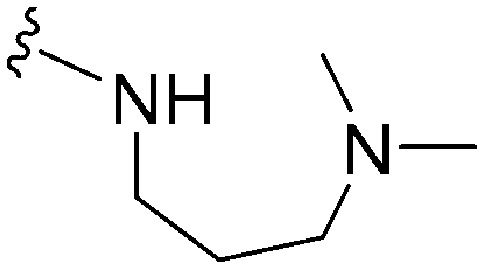	248	39	0.16	ND	ND	6.04	74.78
**76**	–Bn	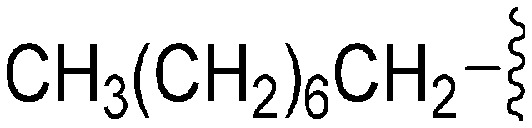	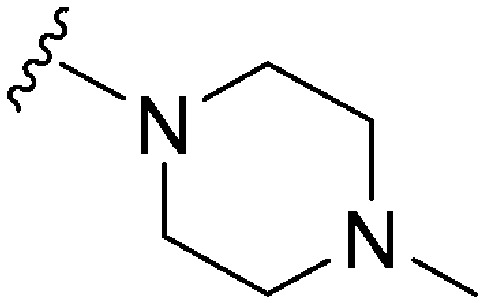	242	1018	4.2	ND	ND	6.21	65.99
**77**	–Bn	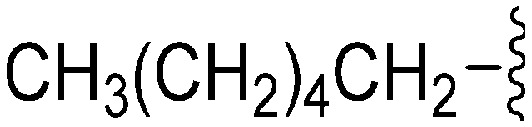	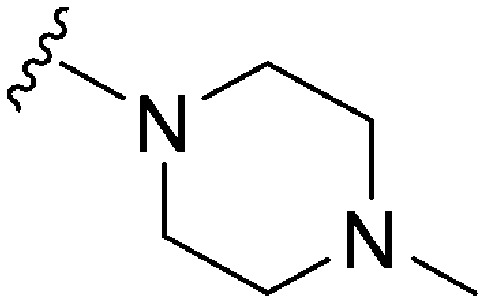	231	114	0.5	ND	ND	5.43	65.99
**78**	–Bn	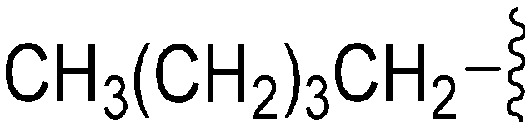	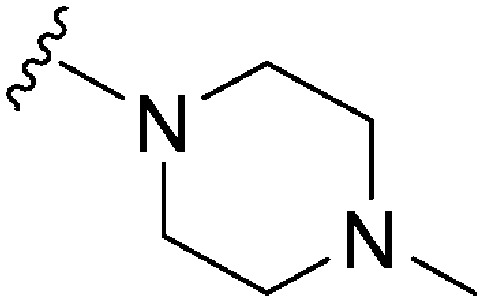	173	∼1000	∼5.8	ND	ND	5.04	65.99
**79**	–Bn	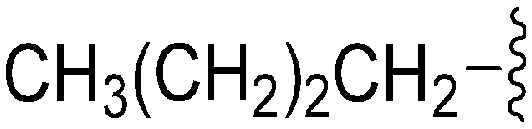	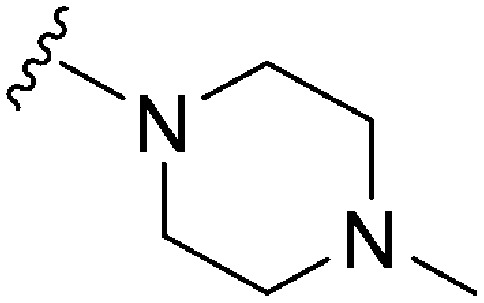	62	683	11.0	2900	46.8	4.65	65.99
**80**	–iPr	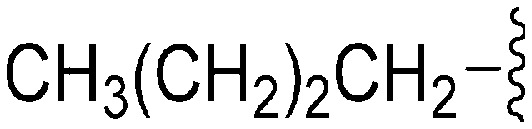	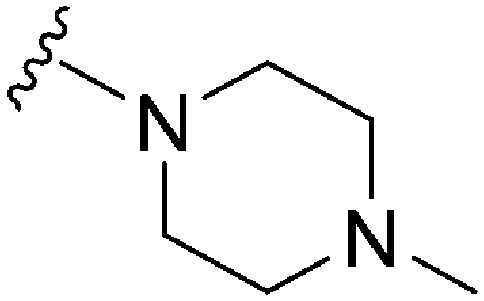	56	843	15.0	4000	71.4	3.85	65.99
**81**	–Bn	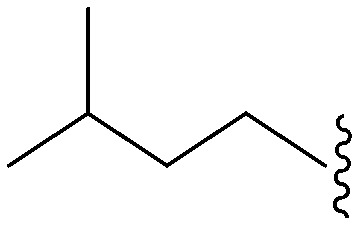	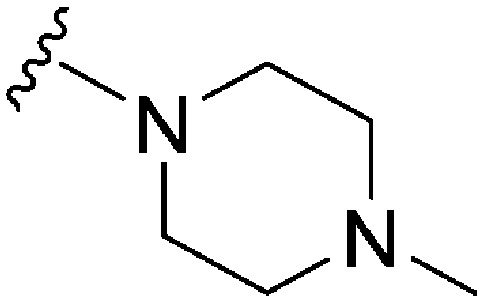	61	5344	87.6	1800	29.5	4.89	65.99
**82**	–iPr	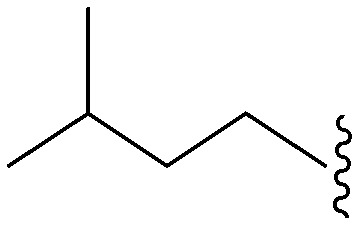	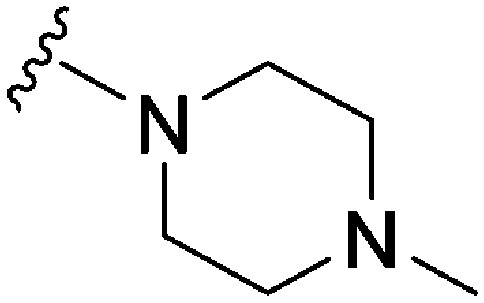	37	2034	55.0	2400	64.9	4.10	65.99
**83**	–Bn	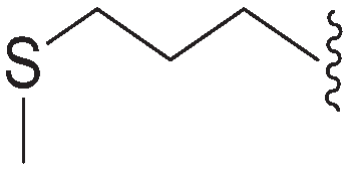	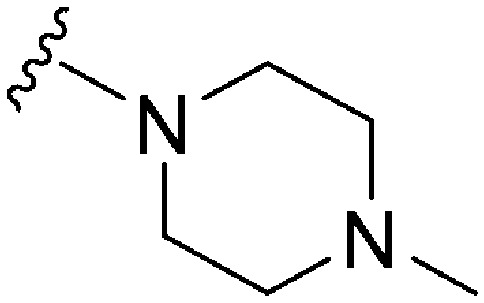	162	223	1.4	ND	ND	4.60	65.99
**84**	–Bn	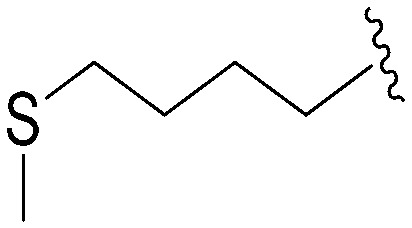	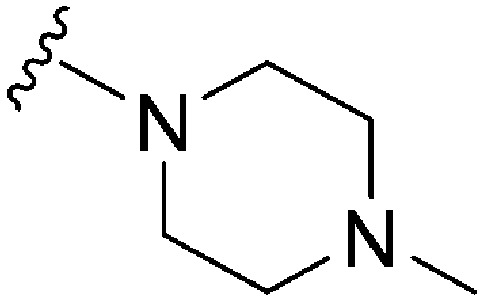	241	575	2.4	ND	ND	4.99	65.99

^*a*^Parasite-killing activity reported earlier.^33^

^*b*^IC_50_ reported using enzyme-coupled SAH detection (ECSD) assay.^36^,^37^

^*c*^IC_50_ reported using chemiluminescence-based oxygen tunnelling (CLOT) assay.^36^,^37^

**Scheme 3:**
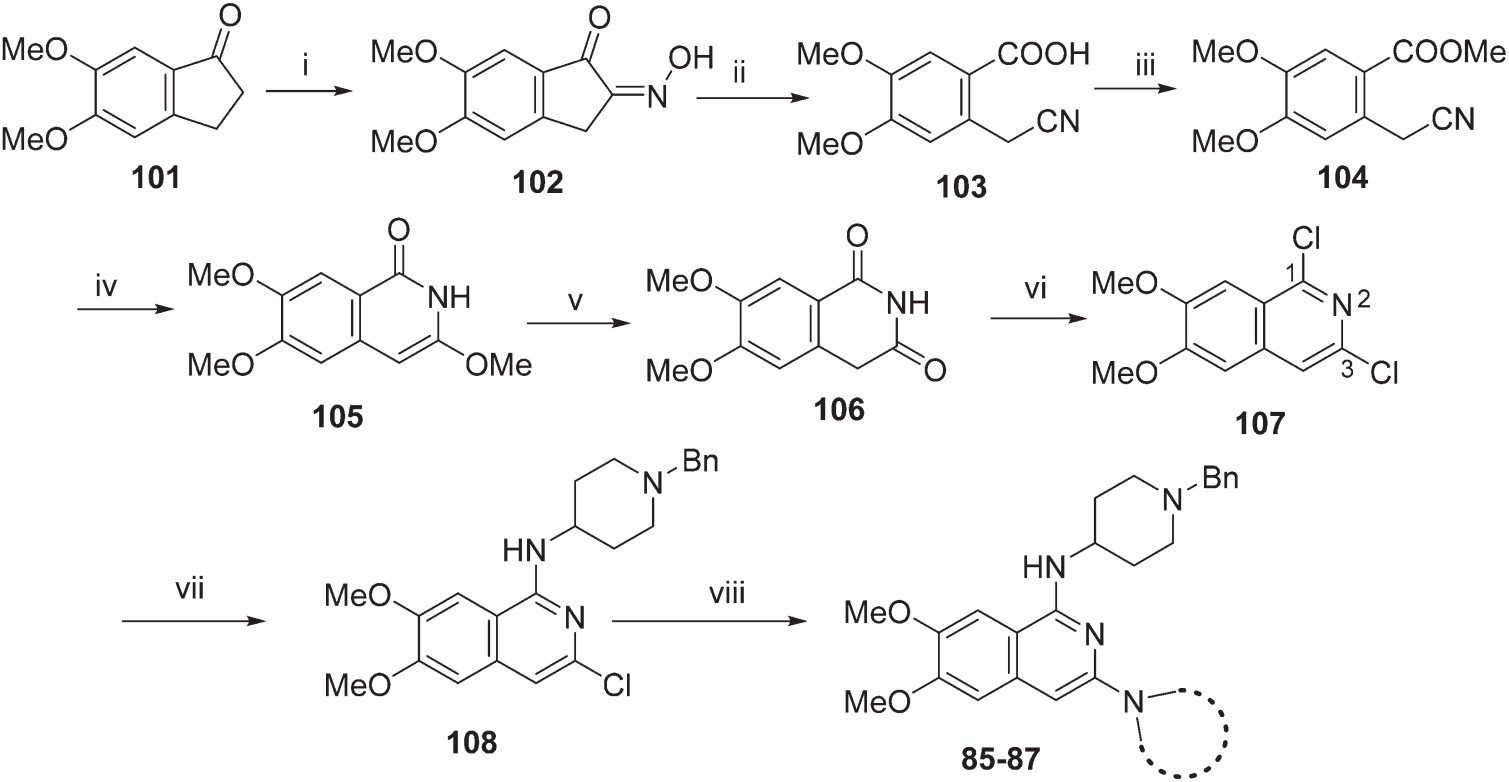
Synthesis of isoquinoline analouges in Table 4. Reagents and conditions: (i) butyl nitrite, HCl (cat.), MeOH, 40 °C, 15 min; (ii) a) NaOH, 50 °C, TosCl b) 80 °C, 15 min; (iii) K_2_CO_3_, MeI, DMF, 2 h; (iv) NaH, MeOH (anhyd), 80 °C, 3 h; (v) 3 M HCl, MeOH, 100 °C, 1 h; (vi) PhP(O)Cl_2_, 160 °C, 3 h, sealed tube; (vii) 180 °C, 1,2-dichlorobenzene, 1-benzyl-4-piperidylamine, microwave, 2 h; (viii) (SPhos) palladium(ii) phenethylamine chloride, K-*t*OBu, various cyclic amines THF, 90 °C, 5–8 h.

**Scheme 4:**
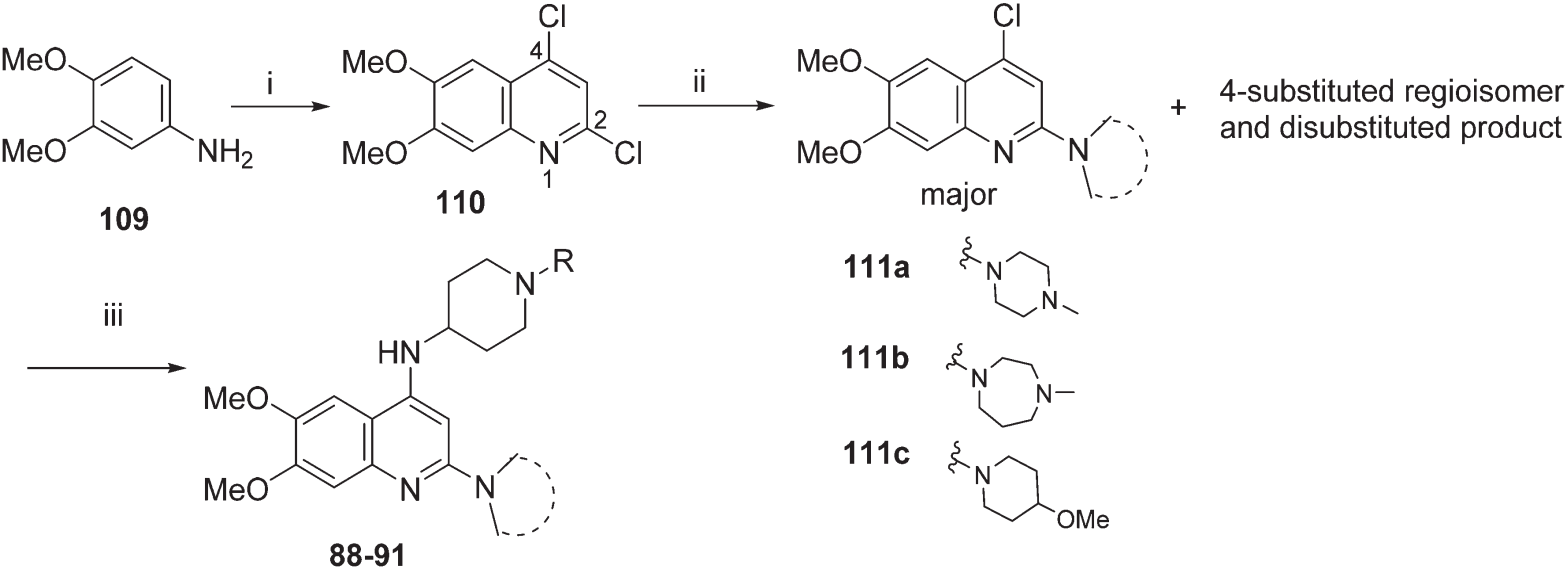
Synthesis of quinoline analogues in Table 4. Reagents and conditions: (i) malonic acid, POCl_3_, reflux, 3 h; (ii) THF, DIEA, various amines, 120 °C, microwave, 48 h; (iii) Pd-PEPPSI-iPr, Li-*t*OBu (1 M in THF), 1-benzyl-4-piperidylamine or 1-methyl-4-piperidylamine, THF, 100 °C, 18 h.

For the synthesis of diaminoisoquinoline and diaminoquinoline analogues **85–91** (Table 4) a route analogous to the synthesis of diaminoquinazolines was envisaged, where the desired amines could be installed late stage. This strategy required the synthesis of the corresponding 1,3-dichloro-6,7-dimethoxyisoquinoline (**107**) and 2,4-dichloro-6,7-dimethoxyquinoline (**110**) intermediates, as shown in Schemes 3 and 4, respectively.

**Table 4:** SAR of pyrimidine ring of quinazoline core

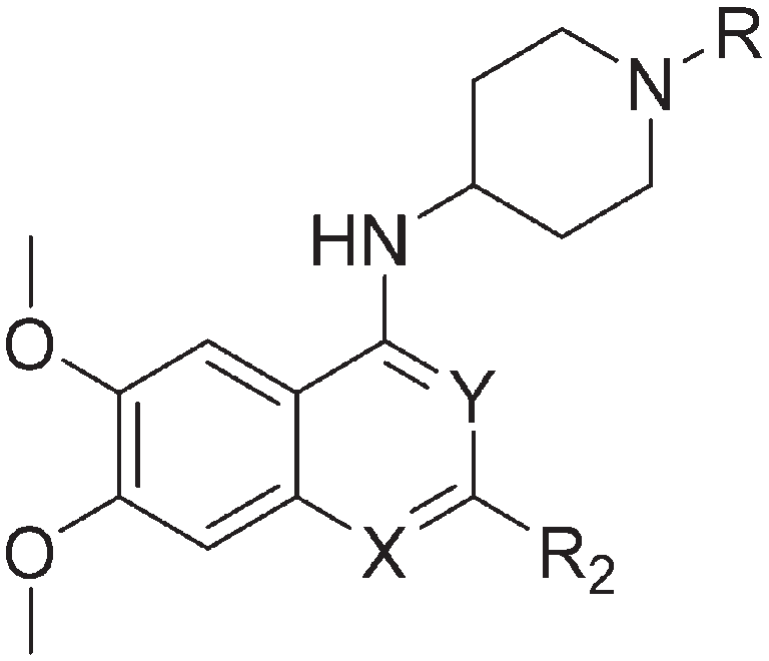											
ID	R	X	Y	R_2_	Pf3D7 IC_50_ (nM)^*^	G9a IC_50_ (nM)^*^	G9a/Pf3D7	HepG2 IC_50_ (nM)	HepG2/Pf3D7	Slog *P*	TPSA
**85**	–Bn	C	N	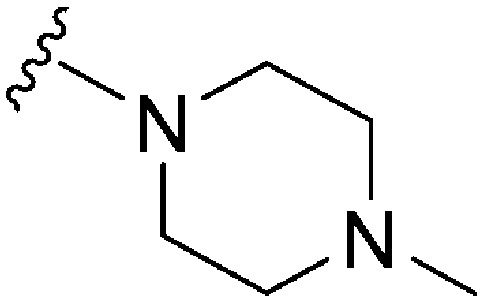	955	NT	—	ND	ND	4.08	53.1
**86**	–Bn	C	N	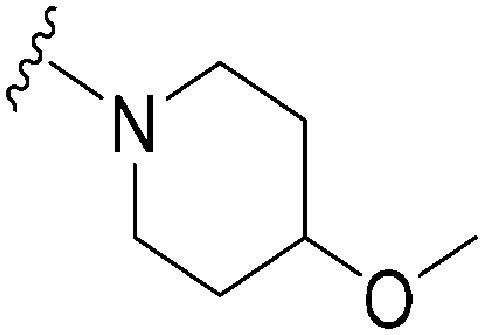	1087	>50 000	>46.00	ND	ND	4.94	59.09
**87**	–Bn	C	N	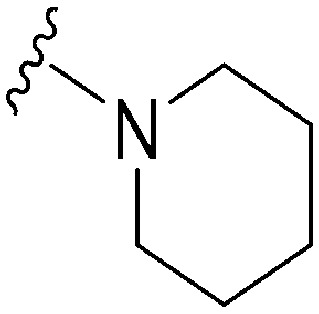	1367	>25 000	>18.3	ND	ND	5.32	49.86
**88**	–Bn	N	C	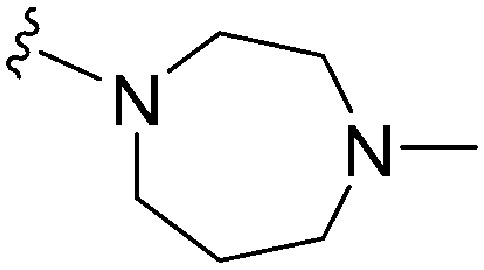	97	13	0.13	5600	57.7	4.47	53.1
**89**	–Bn	N	C	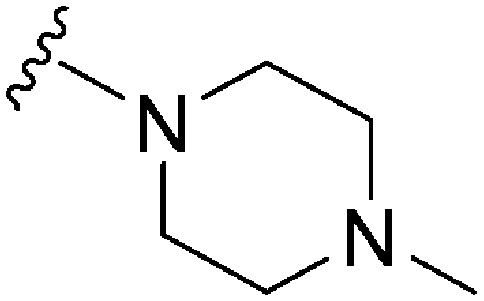	46	21	0.46	3400	73.9	4.08	53.1
**90**	–Bn	N	C	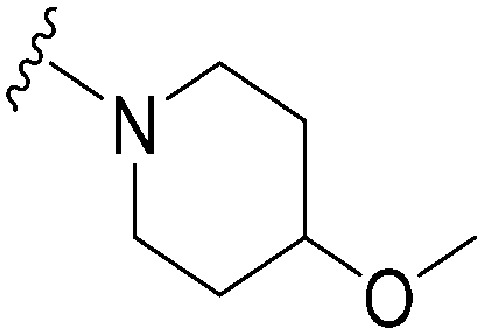	49	119	2.4	3600	73.5	4.94	59.09
**91**	–Me	N	C	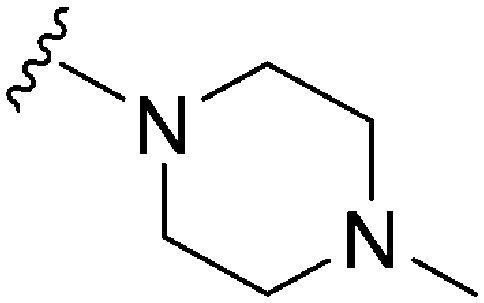	203	30	0.15	13 500	66.5	2.51	53.1

Following a reported procedure, 5,6-dimethoxy-1-indanone (**101**) was treated with butylnitrite under acidic conditions to yield keto-oxime **102** (Scheme 3).^44^,^45^ Compound **102** was tosylated and ring-opened in a one pot^46^ procedure to give the benzoic acid derivative **103** that was esterified to obtain **104**. Subsequent, addition of methoxide to the nitrile moiety of **104**, followed by cyclisation resulted in the isoquinolin-1-one derivative **105** that was further hydrolysed under acidic conditions to obtain dihydroisoquinoline-1,3-dione (**106**). Attempts to convert **106** to the key intermediate 1,3-dichloro-6,7-dimethoxyisoquinoline (**107**) using widely-used chlorinating reagents, such as phosphorous oxychloride, phosphorous pentachloride or thionyl chloride, all failed with little/no conversion of the starting material. Finally, the use of dichlorophenylphosphine oxide^47^ at high temperature was identified as a suitable method to obtain **107** in good yield.

It has been previously reported that conversion of 1,3-dichloroisoquinoline (with no methoxy groups in position 6 and 7) to diaminoisoquinolines is possible under thermal conditions, albeit in low yield.^48^ Notably, the first substitution reaction (with ammonia or a primary amine) was reported to occur at the more reactive position 1 at 100 °C, while temperatures up to 220 °C were used for the second substitution reaction (using a secondary amine) at position 3. However, in case of **107**, which contains methoxy groups in positions 6 and 7, a higher temperature was required for the first substitution reaction. Hence, treatment of **107** with 1-benzyl-4-piperidylamine at 180 °C resulted in the substituted isoquinoline analogue **108**. Prolonged reaction time or the use of higher temperatures resulted in significant degradation of the starting material. Attempts to substitute position 3 of **108** with either ammonia or a secondary amine at temperatures of up to 220 °C proved fruitless, resulting in either no conversion, or degradation of **108** into a complex intractable mixture. Given this, a Buchwald–Hartwig C–N coupling reaction was investigated as an alternative approach. While a comparable coupling reaction to mediate C–N bond formation at position 3 was not found in the literature, one example was identified which described the coupling between an aromatic amine and position 1 of a 1-chloro-3-aminoisoquinoline using palladium acetate and BINAP under microwave irradiation.^46^ Based on the precedent of successful coupling between heteroaromatic halides and cyclic secondary amines using Pd-PEPPSI-iPr,^49^–^51^ we initially surveyed this catalyst for coupling of **108** and cyclic amines. However, low conversion and an intractable mixture of components was obtained under the standard coupling conditions. Hence, dialkylbiaryl phosphane based palladium catalysts were employed which are known for their high stability^52^ and broad applicability^53^ especially for arylchloride substrates.^54^ The use of a SPhos based palladium catalyst in particular resulted in excellent reaction between **108** and a variety of cyclic amines (Scheme 3). Through this method, sufficient quantities of analogues **85–87** were obtained for the current study. 2D NMR (NOESY) of the final compounds was employed to confirm the regiochemistry of amine substitution (see ESI‡).

For the synthesis of 6,7-dimethoxy-2,4-diaminoquinoline analogues (**88–91**) we modified our earlier adopted synthetic methodology,^41^ which contained a number of non-optimal synthetic steps.^55^,^56^ Thus, the key intermediate, dimethoxy-2,4-dichloroquinoline (**110**), was synthesized by heating 3,4-dimethoxyaniline (**109**) and malonic acid with phosphoryl chloride, following a reported procedure (Scheme 4).^57^ Compound **110** was subsequently heated with a variety of secondary amines to give the desired 2-substituted isomers **111a–c**, together with minor amounts of the 4-substituted regioisomer and 2,4-disubstituted products. These minor side-products were readily removed by column chromatography. The conditions reported for this reaction in Scheme 4 were found to be optimum in order to obtain the maximum yield of the desired isomers, often together with recovery of the unreacted starting material **110**. The final structures of the regioisomers were confirmed by either 2D NMR (NOESY) and/or single X-ray crystallography (for **111a**, see Fig. SF1 ESI‡). Finally, as was developed for the isoquinolines, the 2-substituted quinolines (**111a–c**) were subjected to a Buchwald–Hartwig amination reaction, using either 1-benzyl-4-piperidylamine or 1-methyl-4-piperidylamine under palladium catalysis, in order to obtain the desired analogues **88–91**. While the Buchwald–Hartwig coupling steps in both Schemes 3 and 4 proved suitable to provide sufficient material for SAR analysis, we note that the methods are not optimized to maximize product yield. Hence there is scope to screen other catalysts, bases and solvents to further improve this synthetic methodology towards diaminoquinolines and diaminoisoquinolines – scaffolds poorly represented in the literature.

## Results and discussion

For the library of compounds synthesised, we measured and compared parasite killing effects with G9a inhibition. For select compounds, we also used a cell-viability counter screen to evaluate potential non-specific host toxicity using a HepG2 hepatoma cell line. We divide our discussion into key regions of SAR for this series.

### Quinazoline SAR: position 2

**Fig. 2:**
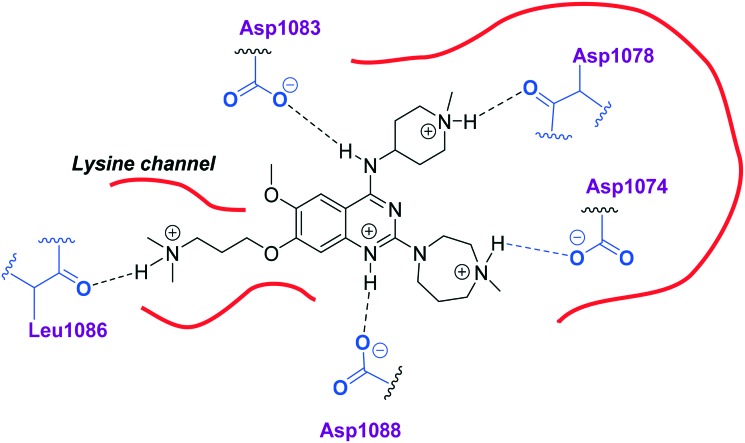
Two-dimensional diagram depicting key interactions of UNC0224 (**66**) in G9a substrate binding pocket (PDB ID ; 3K5K).

Initially, we explored the effect that the amine at position 2 had on parasite-killing and G9a inhibition, when 1-benzyl-4-piperidylamine was present at position 4 (Table 1). Previously, we have shown that decreasing the ring size at position 2, as in the case of compound **8**, or removal of the basic nitrogen from *N*-methyl homopiperazine (**9** and **10**) does not affect the parasite-killing activity.^33^ However, analogues **9** and **10**, lacking a basic nitrogen group in the ring, were found to be 4–5 fold less potent against G9a compared to their counterparts **1** and **2**, which is in accordance with an earlier report.^36^ The protonated *N*-methylhomopiperazine is known to foster a hydrogen bond (H-bond) with the Asp1074 residue in the substrate binding pocket of the G9a (Fig. 2) thereby contributing to overall binding strength. However, this loss of binding contribution can be compensated by a ‘lysine mimic’ group at position 7, as evidenced by the high reported potencies of analogues such as **2**, which contains a cyclohexyl ring at position 2.^39^ A variety of substituted rings that lacked such a basic nitrogen (*e.g.***9–16** and Table ST1 ESI,‡) exhibited poorer G9a inhibition than **1**, whereas parasite-killing activity was less effected. In particular, bulky cyclic amines at position 2, such as in **14–16**, were found to be suitable for maintaining high parasite-killing *vs.* G9a inhibition, especially in the absence of a ‘lysine mimic’.^38^ Interestingly, substitution with a primary acyclic amine instead of a cyclic amine at position 2 (**17**) resulted in poor potency against *Plasmodium* while maintaining good G9a inhibition. Together, these results suggest that the substituent at position 2 can have a significant effect on both parasite-killing and G9a inhibition. Importantly, removal of the basic centre from position 2, together with the use of bulky substituents, can be used to improve the parasite to G9a inhibition ratio.

### Quinazoline SAR: position 4

Next we examined the effect of the substituent at position 4 of the diaminoquinazoline scaffold in conjunction with a variety of substituents at position 2. According to the crystal structure of **1** bound to GLP,^58^ a close homologue of G9a, the benzyl moiety of the 1-benzyl-4-piperidylamine is solvent exposed and thus, its removal or modification should not have considerable effect on G9a inhibitory activity. This fact is clearly apparent in previously described SAR studies of quinazoline G9a inhibitors.^36^,^37^ Indeed, analogues with methyl (**18–20**), fluoro (**21–23**) or methoxy (**24**) substitution on the phenyl ring of the 1-benzyl-4-piperidylamine displayed good G9a IC_50_ values, ranging from 78–169 nM (Table 2). However, once again the effect of a basic nitrogen within the substituent at position 2 was found to be key to potent G9a inhibition, where analogues **25–29** – possessing a piperidine in place of *N*-methylpiperazine – consistently exhibited lower potency against G9a (IC_50_ = 690 to 1214 nM). In contrast, all these analogues displayed good to moderate (IC_50_ < 150 nM) parasite-killing activity, (except **24** and **28**) irrespective of the position 2 substituent. Together these results suggest that the benzyl group of 1-benzyl-4-piperidylamine can be modified without losing significant activity against either G9a or *Plasmodium*, thus providing a handle for tuning the physicochemical parameters of compounds for lead optimization.

Interestingly, analogues with 1-benzyl-3-piperidylamine (**30**, **31**) or 1-benzyl-3-pyrrolidinylamine (**32–34**) at position 4, were less potent against G9a compared to compounds bearing a 1-benzyl-4-piperidylamine in this position, for example **8***vs.***30** and **32**, **10***vs.***31** and **33**, and **15***vs.***34** (see also Table ST1, ESI‡). The protonated basic ring nitrogen of the piperidylamine moiety is expected to form an H-bond with the backbone carbonyl of Asp1078 in G9a (Fig. 2).^37^ However, this interaction is dependent on the conformation of the six-membered piperidylamine, which might present the protonated nitrogen N–H either towards (PDB ; 3K5K)^37^ or away (PDB ; 3RJW)^39^ from Asp1078. In analogues **30–34**, the corresponding basic nitrogen is positioned differently within the rings and thus likely disrupts the H-bond with Asp1078. Such analogues have not been previously reported in the G9a inhibitor literature and therefore present a new aspect of SAR for this series. However, it should be noted that indolylamine based analogues such as **5** (A-366, Fig. 1) possess potent G9a activity^59^ despite the absence of a basic centre in the corresponding position; suggesting that maintenance of an interaction with Asp1078 is not critical for potent G9a inhibition *per se*, but can be compensated by other interactions; particularly those provided by a ‘lysine mimic’. In general, **30–34** retained good (93 nM) to moderate (330 nM) activity against Pf3D7, depending on the substituent at position 2. This suggests that an equivalent H-bond interaction is either maintained despite the different orientation in the PfHKMT target(s) or is not required for activity. The importance of this basic centre was further explored by testing analogues **35–37** with an acylated piperidylamine nitrogen. All such analogues were found to be completely inactive against G9a, while a few (**35–37**, see also, Table ST1 ESI‡) retained moderate parasite-killing activity, depending on the nature of the substituent at position 2.

Replacing 1-benzyl-4-piperidylamine with 1-methyl-4-piperidylamine at position 4 in **1** or **8**, thus yielding **38** and **39** respectively, is known to retain G9a activity due to the solvent exposed nature of the terminal substituent group (*vide supra*).^36^,^37^ However, these compounds were both found to have low Pf3D7 activity. Both **38** and **39** have a calculated topological surface area (TPSA) identical to **1**, but have a significantly lower clog *P*. Previously, diaminoquinazoline analogues possessing lower clog *P* values were found to have poor G9a activity in the cell based assays, suggesting poor permeability across cell membranes.^38^ In accordance with this, the poor parasite-killing activity exhibited by **38** and **39** may be due to their limited cellular uptake, rather than associated to a target-based effect. Indeed, analogues **40** and **41**, with increased clog *P* were found to recover the parasite-killing activity and displayed Pf3D7 IC_50_ values comparable to **1**. Conversely however, analogues **40** and **41** were weak inhibitors of G9a, either due to the absence of a basic centre (as in **40**) or the presence of a bulky substituent (as in **41**) at position 2 (*vide supra*). Remarkably, **42** and **43**, previously reported to be weak inhibitors of G9a,^36^,^37^ displayed excellent parasite-killing activity. To further investigate the effect of increased clog *P* on parasite-killing activity we synthesized analogues having cyclohexylmethyl (**44–47**) or cyclohexyl (**48–50**) *N*-capping groups on the 4-substituent. These lipophilic rings have previously been reported to improve the cellular permeability of G9a inhibitors bearing a ‘lysine mimic’ at position 7.^38^ However, the parasite-killing activity of most of these analogues was less potent than **1**, suggesting no direct correlation between clog *P* and the anti-*Plasmodium* activity. Broadly, analogues lacking a basic nitrogen in the 2-substituent ring exhibited better potency against *Plasmodium* compared to the analogues containing *N*-methylpiperazine (**45** and **49**) and *N*-methylhomopiperazine (**44** and **48**) at this position. A similar trend in parasite-killing activity was observed for molecules containing a 1-isopropyl-4-piperidylamine, cyclohexylamine or aniline at position 4 (**51–59**): analogues bearing a piperidine derivative at position 2 (such as **53**, **54**, **58** and **59**) exhibited better anti-*Plasmodium* activity than comparable compounds with a piperazine or homopiperazine substituent in this position (*e.g.***51**, **52**, **55** and **57**). As observed earlier, the trend for the G9a inhibition was opposite to the anti-*Plasmodium* activity in this regard: analogues with a piperazine or homopiperazine in the position 2, such as **44**, **45**, **48**, **49**, **51** and **52**, were found to be more potent against G9a compared to those without such substituents (such as **46**, **47**, **54**). Analogues having either a cyclohexylamine (**55** and **56**) or aniline (**57–59**) at position 4 were found to be devoid of G9a inhibition, while maintaining good to moderate (77–570 nM) parasite-killing activity, the precise potency of which depended on the substituent at position 2 (see also Table ST1 in ESI‡). This SAR can once again be justified through the necessity of a hydrogen bond with Asp 1078 for G9a inhibition, but not for anti-parasitic activity. Substitution of other amines such as isopropylamine, 1-methyl-3-pyrrolidinylamine or tetrahydro-2*H*-pyran-4-ylamine at position 4 (Table ST1, ESI‡) resulted in poor anti-*Plasmodium* and G9a inhibition.

In summary, modification of the benzyl moiety of 1-benzyl-4-piperidylamine at position 4 or replacing it with a range of other groups can be tolerated to retain both G9a and Pf3D7 activity, while other amine at position 4 are detrimental to G9a activity. This is presumably due to the loss of the H-bond with Asp1078. Pf3D7 activity does not follow this trend however and many a large variety of amines at position 4 are tolerated. In particular, an aromatic amine at this position can be used to achieve high selectivity in favour of anti-*Plasmodium* activity.

We note that methylation of the 4-amino group, or its substitution with an oxygen or sulphur, was found to dramatically decrease the parasite-killing activity of this series (Table ST2, ESI‡).^33^ For G9a inhibition, such a structural alteration eliminates a hydrogen donor interaction with Asp1083 (Fig. 2), and is thereby known to abolish G9a inhibition;^37^ which was further confirmed by the G9a inhibition data obtained by us (Table ST2, ESI‡). This highlights the importance of a hydrogen bond donor at position 4 for both anti-*Plasmodium* and G9a inhibition, thus making it an indispensable SAR feature for both targets. We note however that once again, a suitable ‘lysine mimic’ can potentially compensate for this effect, in terms of G9a potency (*cf.***5**).^59^

### Quinazoline SAR: position 7

As already stated, installation of a ‘lysine mimic’ at position 7 has been found to be central to the development of potent G9a inhibitors.^36^–^40^,^59^ Interestingly, analogues devoid of the methoxy groups at position 6 and 7 were found to maintain moderate parasite-killing activity, but exhibited no G9a inhibition (Table ST3, ESI‡) as also reported earlier by us and others.^41^,^60^ This difference may indicate the inherent differences in the respective lysine binding channels of the G9a and putative PfHKMT target(s), since the methoxy groups are known to occupy regions close to the lysine binding channel in G9a (*vide infra*).^36^,^37^ Focusing specifically on position 7, our previous study reported **60** (Table 3), a positional isomer of **1**, to be equally efficacious against *Plasmodium.*^31^,^32^ Interestingly, such ‘swapping’ of the terminal benzyl and methyl groups between positions 4 and 7 (**1***vs.***60**) resulted in a complete loss of G9a activity. Analogues **61** and **62** also maintained this selectivity for the parasite, exhibiting virtually no G9a inhibition. However, analogue **63** with a primary acyclic amine at position 2 lost activity against *Plasmodium*. This is in accordance with the similar effect observed above for analogue **17** (Table 1), more broadly suggesting a primary amine at position 2 to be deleterious to parasite activity.

Analogues with linear 7-aminoalkoxy substituents (or ‘lysine mimics’) such as **64–67** (Table 3) exhibit potent activity against G9a due to the additional interactions in the lysine binding channel of this enzyme (Fig. 2).^36^–^40^,^59^ However, all these analogues were found to lack anti-*Plasmodium* activity as reported by us previously (see also Table ST4, ESI‡).^33^ We previously rationalized the poor parasite-killing activities of these analogues as attributed to their low clog *P* and/or high TPSA and hence poor cellular permeability. However, when considering such factors more broadly, it is apparent that the calculated physicochemical parameters for **64** and **65** are comparable to **1**. Indeed, both these analogues have been reported to possess moderate G9a activity in cells as measured by the reduction of H3K9me2 levels in an In-Cell Western (ICW) assay, suggesting sufficient cellular permeability of these compounds, at least under the assay concentrations employed.^38^ Hence, we synthesized analogues **68–70** with a higher clog *P* while retaining the basic ‘lysine mimic’ group. All three analogues exhibited potent G9a activity, as expected, but showed only slight improvement in parasite-killing activity. In contrast, replacement of the basic ‘lysine mimic’ with less polar ether (**71**) or neutral hydrocarbon (**72**) chain further improved the parasite-killing activity while it significantly reduced the G9a potency of these analogues. Comparison of **72** and **69** is particularly interesting as both analogues have very similar clog *P* and TPSA values, but the former is ∼4 fold more potent against *Plasmodium* while reported to be considerably less potent against G9a.^37^ Together, this data suggests that unlike G9a, the lysine binding channel in the PfHKMT target(s) is better able to accommodate the hydrophobic benzyl or hydrocarbon chains. Indeed, computational analysis of lysine binding channels of various HKMTs suggests that they have diverse hotspot profiles that can be exploited for designing selective inhibitors.^61^

A number of recent reports suggest that for cases (in cancer) where the substrate histone lysine residues are mutated to either methionine (Met) or a hydrocarbon based residue such as norleucine (Nle) (*e.g.* H3K9M or H3K27M), these mutant histones act as potent inhibitors of the respective HKMTs: such as G9a inhibition by H3K9M or polycomb repressive complex 2 (PRC2) inhibition by H3K27M.^62^–^68^ Very recently Judge *et al.* have reported peptide inhibitors of SETD8 (a H4K20 methylase) by replacing the lysine (K20) residue of the substrate peptide to Nle/Met (H4K20Nle/Met) and other hydrophobic residues.^69^ Indeed, co-crystallized structures of such mutated peptide substrates with both human PRC2/G9a/SETD8 show Met and Nle to occupy the lysine binding channel in a manner similar to lysine in the non-mutated substrates.^67^–^69^ This data clearly suggests that ‘lysine mimic’ groups which interact with the lysine binding channels of HKMTs need not be limited to polar and basic sidechains. Given this, and given the fact that hydrocarbon chains were tolerated at position 7 for anti-parasite activity, analogues containing ‘Met or Nle mimics’ at position 7 were investigated. However, poor inhibition of G9a by **72** suggested that the length and nature of the ‘Nle mimic’ in context of diaminoquinazoline inhibitory scaffolds was not directly analogous to that in H3 peptides. Analogues **73–77** with a 6–8 carbon linear ‘Nle mimic’ all displayed very similar moderate IC_50_ values (∼231–268 nM) against the parasite. On the other hand, analogues with the linear or branched ‘Nle mimic’ containing 4–5 carbons (**78–82**) showed good parasite-killing activities, with **82** exhibiting an IC_50_ value (37 nM) comparable to **1**. The G9a inhibitory SAR was found to be different however: a linear chain of 6 carbons was found to be optimum for G9a inhibition as evidenced by the low G9a IC_50_ values of **74**, **75**, and **77**. Indeed, G9a inhibition seemed to be intolerant of chain length variations, as increasing (**73**, **76**) or decreasing (**78–82**) the chain length led to a negative effect on inhibitor potency. To the best of our knowledge, analogues **74**, **75** and **77** represent the first examples of non-peptidic small molecule G9a inhibitors possessing a hydrophobic ‘Nle mimic’ at position 7. Analogues **83** and **84** representing a ‘Met mimic’ showed only moderate parasite-killing activities and poor G9a inhibition. This is in accordance with the data reported for peptide based inhibitors where Met mutation was found to be less effective than Nle mutation.^64^ However, assessment of analogues possessing varying length ‘Met mimics’ and different position 2 substituents would need to be tested in order to draw firm conclusions over the SAR surrounding ‘Met mimics’.

### SAR for the central heterocyclic ring

Finally, we examined the role of the central fused aromatic scaffold. We have previously synthesized and surveyed other fused heteroaromatic scaffolds, related to diaminoquinazolines, against G9a.^41^ Specifically, we examined cases where the fused benzenoid ring was replaced by a thiophene, furan, imidazole or a cyclopentane ring (Table ST5, ESI‡). All these analogues were found to be inactive against both G9a and *Plasmodium* suggesting both biological activities to be restricted to the fused six membered ring scaffold. Next, we tested the importance of pyrimidine ring nitrogens of the quinazoline scaffold, by comparing analogues based on diaminoquinolines and diaminoisoquinolines (Table 4). The protonated N-1 nitrogen of quinazoline interacts strongly with Asp1088 (Fig. 2) in the G9a pocket, while the N-3 nitrogen does not appear to form any specific interaction. Accordingly, isoquinoline analogues (**85–87**) lacking a basic centre at position 1 displayed poor activities against both G9a and the parasite while the quinoline compounds **88–91** were found to be potent against both targets. In particular, **88** displayed ∼5 fold increase in potency against G9a than the parental analogue **1**; a fact that we have rationalised previously.^41^ This data highlights this SAR feature to clearly be conserved between G9a and the potential PfHKMT target(s) of this compound series.

### HepG2 cytotoxicity

Earlier, we screened several diaminoquinazoline analogues in a cell-viability assay to evaluate potential host toxicity using a HepG2 hepatoma cell line.^33^ The HepG2 cell line is routinely used to measure toxicity^70^–^73^ and displays slightly more sensitivity compared to the other commonly used cell lines.^72^ Of the select analogues examined in this assay, most display high anti-HepG2 IC_50_ values. No correlation is observed between the anti-*Plasmodium* and anti-HepG2 activities. For example, analogues **12**, **40**, **42** and **43** exhibit potent parasite-killing activity but are amongst the least toxic compounds studied, with anti-HepG2 IC_50_ > 10 μM. While lipophility can give rise to non-specific cellular toxicity,^74^ we did not observe any significant correlation between the cLog *P* and anti-HepG2 activity. Finally, no clear correlation is apparent between G9a inhibition and anti-HepG2 activity. For example, BIX01294 (**1**) has a G9a IC_50_ of 67 nM and a HepG2 IC_50_ of 4.8 μM, whereas compound **14** is inactive against G9a but has a HepG2 IC_50_ of 3.6 μM. The compounds that were most active (IC_50_s between 1.8–2.9 μM) against the HepG2 cell line had a (methylbenzyl)piperidin-4-yl group at position – 4 (such as **25–27**) and benzyl/alkyl group at position 7 (such as **61**, **62**, **79**, **81** and **82**). Together, this data suggest that the reported analogues generally have a very promising differential activity for parasites over HepG2 human cells.

### Further activity against *Plasmodium*

**Fig. 3:**
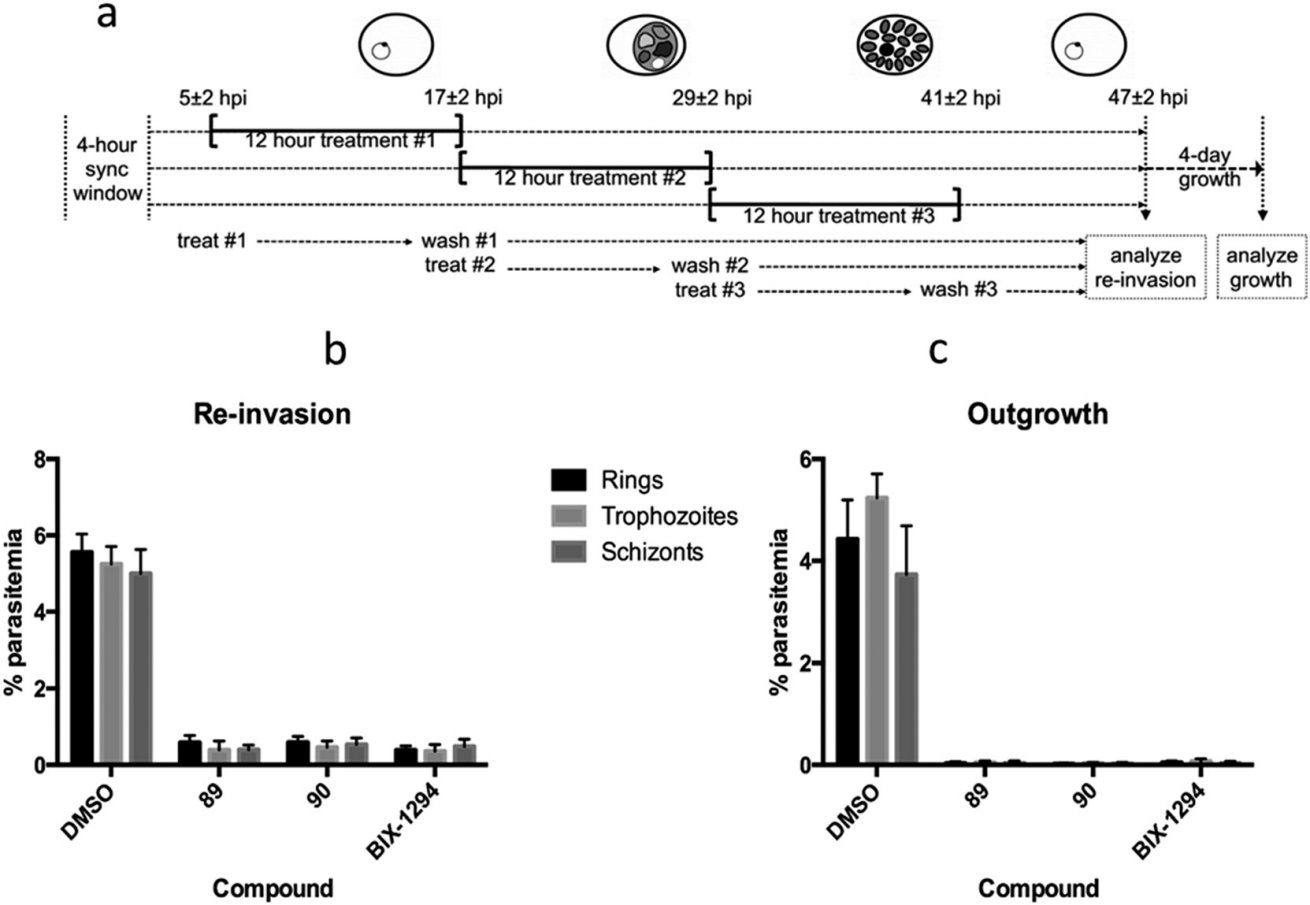
Stage-dependent antimalarial activity a) synchronised *P. falciparum* parasites were treated with DMSO or 10 × IC_50_ values of compounds **89**, **90** or BIX01294 for three distinct 12 hour periods of the intra-erythrocytic life cycle b) re-invasion of treated parasites at 47 h post-invasion was quantified by flow cytometry. c) After 12 hour treatment, parasites were washed, diluted, and allowed to grow for four days, after which parasitemia was measured by flow cytometry. Data are the mean ± SD of 30 000 RBCs from duplicate samples.

We have previously shown the diaminoquinazoline chemotype, as exemplified by analogues **1**, **12**, **40** and **60**, to exhibit a fast parasite-killing phenotype, effective throughout the intra-erythrocytic parasite life cycle. Killing phenotypes are a function of the parasite molecular target(s) of a given compound^75^ and thus, in order to study whether the diaminoquinoline chemotype has a comparable target profile to the diaminoquinazolines, compounds **89** and **90** were employed in analogous phenotypic assays. Hence, highly synchronised parasites were treated with **89** and **90** for three distinct periods of the 12 hour intra-erythrocytic stage and re-invasion into the next cycle and out-growth two cycles later was quantified (Fig. 3). The data revealed **1**, **89** and **90**, to possess a similar erythrocytic stage-independent killing phenotype, suggesting a common target profile for the anti-*Plasmodium* activity of both the diaminoquinolines and the diaminoquinazolines.

### Reduction in histone methylation levels in *Plasmodium*

**Fig. 4:**
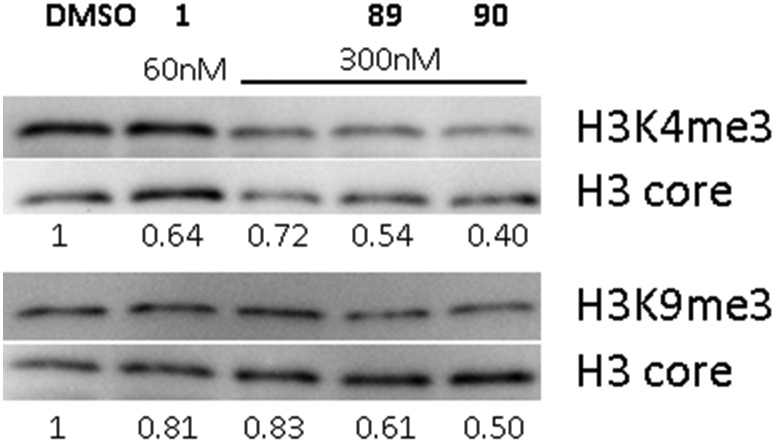
Effect of **1**, **89** and **90** on the histone methylation levels in treated parasites. *P. falciparum* 3D7 parasites were treated with the indicated compounds or DMSO control for 12 hours. Specific histone H3K4me3 and H3K9me3 levels were quantified by densitometry, normalised to histone H3 core signal, and the resulting methylation levels relative to DMSO control-treated parasites are indicated below each pair of methylation-specific and corresponding core histone H3 bands.

Earlier, we demonstrated that BIX01294 and other diaminoquinazoline analogues decrease H3K4me3 and H3K9me3 levels in parasites in comparison to DMSO vehicle.^31^,^33^ Since, the diaminoquinolines such as **88–91** retain the parasite-killing activity, potentially by acting on the same PfHKMTs targets, we evaluated **89** and **90** in the same assay. Thus, malaria parasites (Pf3D7) were treated with **1**, **89** and **90** and the histone H3K4me3 and H3K9me3 levels were analysed by western blot (Fig. 4) in the treated parasites, relative to control parasites treated with DMSO. As expected, the data demonstrates a decrease in H3K4me3 and H3K9me3 levels upon treatment of parasites with **1** and diaminoquinoline analogues **89** and **90** suggesting diaminoquinolines share the same target/mechanism as the diaminoquinazoline analogue BIX01294. Together, this supports our SAR studies demonstrating that both diaminoquinazoline and diaminoquinoline analogues retain the essential features for binding G9a and the related PfHKMT target in parasites.

## Conclusion

**Fig. 5:**
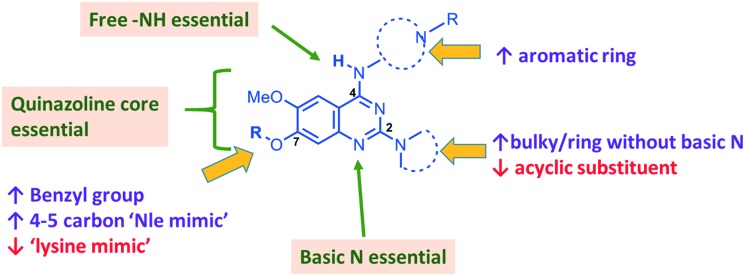
Summary of key SAR features of diaminoquinazoline analogues against G9a and *Plasmodium* and suggestions for designing parasite-selective analogues. Features highlighted in boxes are essential for both G9a and anti-*Plasmodium* activity. Acyclic substituent at position 2 and a ‘lysine mimic’ at position 7 were found to be unfavourable (↓) for parasite-killing activity. Conversely, bulky or a ring without basic centre at position 2, aromatic ring at position 4 and a benzyl/4-5 carbon ‘Nle-mimic’ at position 7 were found to impart selectivity in favour (↑) of the anti-*Plasmodium* activity.

In summary, we have synthesized and tested a large number of diaminoquinazoline and related analogues against G9a and *P. falciparum*, in order to elucidate more complete SAR features for both targets (as summarized in Fig. 5). In addition to reconfirming some of the previously reported independent SAR features for these targets, our work provides new insight. For example, previous G9a SAR for this series^36^–^40^ has mostly focused on analogues possessing a ‘lysine mimic’ at position 7 and hence the effect of substituents at position 2 and 4 was not known in the absence of a position 7 lysine mimic. Our results suggest that diaminoquinazoline analogues display different SAR trends against G9a depending on whether a ‘lysine mimic’ is present or not. For instance, we found that in the absence of a ‘lysine mimic’ at position 7, basic centres at both position 2 and 4 and a hydrogen bond donor at position 4 are required for potent G9a inhibition. Additionally, the analogues lacking a ‘lysine mimic’ group do not tolerate bulky cyclic amine at position 2 and loose significant potency against G9a. Perhaps more importantly, the polar and basic ‘lysine mimic’ at position 7 – now well established for the widely employed G9a inhibitors UNC0638 and UNC0642^37^,^38^ – can be replaced by hydrophobic ‘Nle mimics’, while retaining good G9a inhibition, as has been reported for the peptide-based inhibitors.^62^–^69^ Consequently, our SAR studies resulted in the discovery of first diaminoquinazoline G9a inhibitors bearing a 6-carbon ‘Nle mimic’ at position 7.

While conserved features of G9a and anti-parasitic SAR could be used to rationalise the likely PfHKMT target(s) of these inhibitors, clearly future optimization of this scaffold will need to diverge from activity on human (host) targets, such as G9a. We have identified key SAR features which demonstrate that high parasite *vs.* G9a selectivity can be achieved by selecting appropriate substituents at position 2, 4 and 7 of the quinazoline ring. For instance, a bulky substituent or a ring lacking a basic nitrogen at position 2 or an aromatic ring at position 7 can be used to design analogues with a high parasite-killing to G9a inhibition ratio. Similarly, we have shown that while a ‘lysine mimic’ substituent position 7 imparts high potency against G9a, it is deleterious to the anti-*Plasmodium* activity. Together, this data suggests that while broadly similar, the G9a and potential PfHKMT target(s) binding pockets and/or binding modes of the diaminoquinazoline analogues exhibit clear and exploitable differences. Thus, there remains significant potential in this series to further develop parasite selective analogues. Based on this, we believe this scaffold to have clear potential for development into a novel and much needed, new medicine for malaria.

Of course, a key question remains for this series: specifically which of the essential PfHKMTs are targeted by these compounds? To date, our study on PfSET7 represents the only successful report^30^ of recombinant expression of an active PfHKMT, suitable for biochemical characterization and study. Initial activity assays using PfSET7 have suggested PfSET7 not to be the target of **1** (data not shown), and therefore the other candidate targets are the remaining essential PfHKMTs: PfSET1, PfSET3, PfSET6, PfSET9, or PfSET10. Future studies aimed at robust biochemical characterisation of our lead inhibitors against all the essential PfHKMTs will be reported in due course, once protocols for successful protein generation have been optimized.

## Experimental

### Chemistry general procedures

All reactions were performed under an atmosphere of dry nitrogen unless otherwise stated. Flash column chromatography was carried out using Merck Kiesegel 60 silica gel (230–400 mesh, 0.040 0.063 mm). Thin layer chromatography (TLC) was performed on aluminium plates using Merck Kiesegel 60 F254 (230–400 mesh) fluorescent treated silica which were visualised under ultraviolet light (254 nm), or by staining with potassium permanganate or ninhydrin solution as appropriate. All ^1^H and ^13^C NMR spectra were recorded using Bruker 400 MHz spectrometers or Bruker AV 500. Chemical shifts (*δ*) are quoted in units of parts per million (ppm) downfield from tetramethylsilane and are referenced to a residual solvent peak. Coupling constants (*J*) are given in Hertz (Hz). The ^1^H NMR spectra are reported as follows: ppm (multiplicity, coupling constants *J*/Hz, number of protons). High and low resolution mass spectrometry (EI, ES, and CI) were recorded on Micromass Platform II and Micromass AutoSpec-Q spectrometers. All compounds tested in biological assays were >95% pure by LCMS. LCMS gradient: from 95 : 5, A : B to 5 : 95, A : B over 18 minutes, where A is water (0.1% formic acid), and B is methanol. Column: XBridge C18 columns with dimensions 4.6 mm × 100 mm.

#### 2-(Hydroxyimino)-5,6-dimethoxy-2,3-dihydro-1*H*-inden-1-one (**102**)

To a solution of 5,6-dimethoxy-1-indanone (**101**, 0.769 g, 4 mmol) in methanol (15 mL) at 40 °C, *n*-butyl nitrite (0.51 mL, 4.4 mmol) was added followed by two drops of concentrated HCl. The reaction mixture was stirred for 15 minutes after which the precipitated solid was filtered, washed with cold methanol and dried to obtain the pure **102** as off white solid (0.791 g, 89%). ^1^H NMR (400 MHz, DMSO-*d*_6_) 12.41 (br s, 1H, D_2_O exchangable), 7.18 (s, 2H), 3.90 (s, 3H), 3.82 (s, 3H), 3.65 (s, 2H); HRMS (+ESI) *m*/*z* calcd for C_11_H_12_NO_4_ 222.0766, found, 222.0762.

#### 2-(Cyanomethyl)-4,5-dimethoxybenzoic acid (**103**)

To a solution of **102** (1.076 g, 4.87 mmol) in aqueous sodium hydroxide (12 mL, 8% w/v) at 50 °C, tosyl chloride (1.206 g, 6.33 mmol) was added. The temperature was raised to 80 °C and the reaction mixture was further heated for 15 minutes. The mixture was allowed to cool to room temperature and extracted with DCM. The aqueous layer was acidified using 6 M HCl resulting in off white precipitates that were dissolved in DCM (∼250 mL). The organic layer was extracted with saturated sodium bicarbonate solution and the aqueous layer was acidified again with 6 M HCl to obtain titled compound as a white solid (0.525 g, 49%). ^1^H NMR (400 MHz, DMSO-*d*_6_) 13.02 (br s, 1H, D_2_O exchangable), 7.49 (s, 1H), 7.13 (s, 1H), 4.18 (s, 2H), 3.84 (s, 3H), 3.80 (s, 3H); HRMS (+ESI) *m*/*z* calcd for C_11_H_12_NO_4_ 222.0766, found, 222.0775.

#### Methyl 2-(cyanomethyl)-4,5-dimethoxybenzoate (**104**)

To a suspension of **103** (100 mg, 0.452 mmol) and potassium carbonate (157 mg, 1.13 mmol) in DMF (2 mL), methyl iodide (34 μL, 0.543 mmol) was added and the reaction mixture was allowed to stir for 2 hours at room temperature. The reaction mixture was filtered and solids washed with ethyl acetate. The organic layer was washed with water, dried over magnesium sulphate and evaporated *in vacuo* to obtain the crude product which was chromatographed over silica gel (DCM : MeOH; 100 : 0 → 98 : 2) to afford titled compound as a white solid (85 mg, 80%). ^1^H NMR (400 MHz, CDCl_3_) 7.57 (s, 1H), 7.01 (s, 1H), 4.21 (s, 2H), 3.98 (s, 3H), 3.93 (s, 3H), 3.91 (s, 3H).

#### 3,6,7-Trimethoxyisoquinolin-1(2*H*)-one (**105**)

To a solution of **104** (0.246 g, 1.05 mmol) in anhydrous methanol (12 mL), sodium hydride (0.105 g, 2.62 mmol, 60% w/w) was added at room temperature. The reaction mixture was stirred until effervescence ceased after which it was heated at 80 °C for 3 hours on an oil bath. The volatiles were removed *in vacuo* and ammonium chloride saturated solution was added to the residue. The resulting solid was filtered, washed subsequently with water and ether and dried *in vacuo* to obtain **105** as a white solid (0.156 g, 63%). ^1^H NMR (400 MHz, DMSO-*d_6_*) 11.47 (s, 1H, br), 7.42 (s, 1H), 7.01 (s, 1H), 5.82 (s, 1H), 3.85 (s, 3H), 3.80 (s, 3H), 3.79 (s, 3H); ^13^C NMR (125 MHz, DMSO-*d_6_*) 161.26, 153.39, 152.20, 146.59, 134.68, 114.91, 106.80, 106.11, 79.96, 55.56, 55.36; HRMS (+ESI) *m*/*z* calcd for C_12_H_14_NO_4_ 236.0923, found, 236.0930.

#### 6,7-Dimethoxyisoquinoline-1,3(2*H*,4*H*)-dione (**106**)

A mixture of **105** (0.623 g, 2.65 mmol) in methanol (25 mL) and aqueous HCl (3 M, 8.8 mL) was heated at 100 °C for 1 hours. The resulting precipitate was filtered off, washed subsequently with water and ether and dried *in vacuo* to obtain **106** as a white solid (0.435 g, 74%). ^1^H NMR (400 MHz, DMSO-*d_6_*) 12.41 (s, 1H), 7.19 (s, 1H), 7.18 (s, 1H), 3.90 (s, 3H), 3.83 (s, 3H), 3.66 (s, 2H); ^13^C NMR (125 MHz, DMSO-*d_6_*) *δ* 171.21, 165.05, 153.38, 148.01, 130.72, 117.13, 109.98, 108.72, 55.87, 55.56, 35.75; HRMS (+ESI) *m*/*z* calcd for C_11_H_12_NO_4_ 222.0761, found, 222.0749.

#### 1,3-Dichloro-6,7-dimethoxyisoquinoline (**107**)

A mixture of **106** (68 mg, 0.308 mmol) and dichlorophenylphosphine oxide (87 μL, 0.615 mmol) was heated in a sealed tube at 160 °C for 3 hours. The reaction mixture was quenched with saturated sodium bicarbonate solution and the aqueous layer was extracted with DCM. The organic layer was dried over magnesium sulphate and concentrated to obtain the crude product which was chromatographed over silica gel (DCM : MeOH; 100 : 0 → 99 : 1) to afford **107** (65 mg, 95%) as a white powder. ^1^H NMR (400 MHz, CDCl_3_) 7.48 (s, 1H), 7.43 (s, 1H), 6.98 (s, 1H), 4.05 (s, 3H), 4.03 (s, 3H); ^13^C NMR (125 MHz, CDCl_3_) *δ* 154.41, 151.32, 148.10, 141.68, 136.28, 121.52, 118.37, 104.19, 104.15, 56.37, 56.28; HRMS (+ESI) *m*/*z* calcd for C_11_H_10_NO_2_Cl_2_ 258.0089, found, 258.0095. MS (+ESI) *m*/*z* 258 [M + H]^+^, 260 (M + 2H)^+^.

#### *N*-(1-Benzylpiperidin-4-yl)-3-chloro-6,7-dimethoxyisoquinolin-1-amine (**108**)

A mixture of **107** (70 mg, 0.271 mmol) and benzyl-4-piperidylamine (0.165 mL, 0.813 mmol) in dichlorobenzene (0.5 mL) was heated at 180 °C for 2 hours in a microwave reactor. The reaction mixture was chromatographed over silica gel (DCM : MeOH; 100 : 0 → 97 : 3) to obtain **108** as a brown solid (36 mg, 32%). ^1^H NMR (400 MHz, CDCl_3_) 7.38–7.27 (m, 5H), 6.87 (s, 1H), 6.84 (s, 1H), 6.83 (s, 1H), 4.83 (s, 1H, br), 4.27–4.20 (m, 1H), 4.00 (s, 3H), 3.98 (s, 3H), 3.61 (s, 2H), 2.96–2.93 (m, 2H), 2.30 (t, *J* = 11.9 Hz, 2H), 2.23–2.14 (m, 2H), 1.67 (ddd, *J* = 22.7, 11.4, 3.5 Hz, 2H); HRMS (+ESI) *m*/*z* calcd for C_23_H_27_N_3_O_2_Cl 412.1792, found, 412.1813.

#### *N*-(1-Benzylpiperidin-4-yl)-6,7-dimethoxy-3-(4-methylpiperazin-1-yl)isoquinolin-1-amine (**85**)

A mixture of potassium *tert*-butoxide (29 mg, 0.26 mmol) and (SPhos)palladium(ii) phenethylamine chloride (CAS no. 1028206-58-7) (4 mg, 0.005 mmol) in anhydrous THF (1 mL) was purged with nitrogen for 10 minutes after which a solution of **108** (43 mg, 0.104 mmol) and *N*-methylpiperazine (14 μL, 0.125 mmol) in 2 mL THF was added to it. The reaction mixture was purged with nitrogen for another 10 minutes and then heated in microwave at 90 °C for 8 hours. The reaction mixture was cooled to room temperature, volatiles were evaporated and the residue was chromatographed over silica gel (DCM : MeOH (7N NH_3_); 97 : 3 → 95 : 5) to obtain **85** as a dark green solid (16 mg, 32%). ^1^H NMR (400 MHz, CD_2_Cl_2_) 7.35–7.22 (m, 5H), 6.80 (s, 1H), 6.79 (s, 1H), 6.03 (s, 1H), 4.72 (s, 1H, br), 4.13–4.06 (m, 1H), 3.89 (s, 6H), 3.52 (s, 2H), 3.45–3.43 (m, 4H), 2.90–2.87 (m, 2H), 2.51–2.49 (m, 4H), 2.30 (s, 3H), 2.23–2.13 (m, 4H), 1.62–1.53 (m, 2H). ^13^C NMR (100 MHz, CD_2_Cl_2_) *δ* 155.09, 152.92, 152.67, 146.89, 136.96, 129.55, 128.53, 127.40, 110.36, 107.01, 105.29, 101.91, 88.81, 63.25, 56.42, 55.91, 55.30, 48.72, 46.26, 46.21, 32.79. HRMS (+ESI) *m*/*z* calcd for C_28_H_38_N_5_O_2_ 476.3026, found, 476.3028.

#### *N*-(1-Benzylpiperidin-4-yl)-6,7-dimethoxy-3-(4-methoxypiperidin-1-yl)isoquinolin-1-amine (**86**)

**86** was synthesized following procedure similar to the synthesis of **85**. The reaction mixture was purified over silica gel (DCM : MeOH (7N NH_3_); 100 : 0 → 98 : 2) to obtain the pure product as a yellow solid (44 mg, 92%). ^1^H NMR (400 MHz, CD_2_Cl_2_) 7.36–7.22 (m, 5H), 6.78 (s, 1H), 6.77 (s, 1H), 6.05 (s, 1H), 4.70 (s, 1H, br), 4.13–4.06 (m, 1H), 3.95 (dt, *J* = 12.7, 4.4 Hz, 2H), 3.93 (s, 3H), 3.92 (s, 3H), 3.54 (s, 2H), 3.39–3.34 (m, 4H), 3.03 (ddd, *J* = 13.0, 9.9, 3.2 Hz, 2H), 2.91–2.89 (m, 2H), 2.25–2.11 (m, 4H), 1.98–1.94 (m, 2H), 1.60–1.52 (m, 4H); ^13^C NMR (100 MHz, CDCl_3_) *δ* 154.62, 152.34, 152.27, 146.31, 136.65, 129.28, 128.26, 127.19, 106.64, 104.96, 101.30, 89.04, 63.03, 56.15, 55.73, 55.50, 52.66, 48.12, 44.17, 32.36, 30.37; HRMS (+ESI) *m*/*z* calcd for C_29_H_38_N_4_O_3_ 491.3022, found, 491.3008.

#### *N*-(1-Benzylpiperidin-4-yl)-6,7-dimethoxy-3-(piperidin-1-yl)isoquinolin-1-amine (**87**)

**87** was synthesized following a procedure similar to the synthesis of **85**. The reaction mixture was purified over silica gel (DCM : MeOH (7N NH_3_); 98 : 2 → 96 : 4) to afford pure product as a yellow solid (15 mg, 42%). ^1^H NMR (400 MHz, CD_2_Cl_2_) 7.36–7.23 (m, 5H), 6.78 (s, 1H), 6.77 (s, 1H), 6.03 (s, 1H), 4.69 (s, 1H, br), 4.13–4.06 (m, 1H), 3.89 (s, 6H), 3.54 (s, 2H), 3.45–3.42 (m, 4H), 2.92–2.89 (m, 2H), 2.24–2.12 (m, 4H), 1.65–1.57 (m, 8H); ^13^C NMR (100 MHz, CD_2_Cl_2_) *δ* 155.52, 152.84, 152.64, 146.59, 137.20, 129.49, 128.51, 127.32, 106.58, 105.18, 101.96, 88.58, 63.31, 56.42, 55.87, 53.80, 48.77, 47.47, 32.90, 25.97, 25.32; HRMS (+ESI) *m*/*z* calcd for C_28_H_37_N_4_O_2_ 461.2917, found, 461.2899.

#### 2,4-Dichloro-6,7-dimethoxyquinoline (**110**)

A mixture of 3,4-dimethoxyaniline (**109**, 10 g, 65.4 mmol) malonic acid (5.4 g, 51.9 mmol) and phosphorous oxychloride (25 mL) was refluxed for 3 hours on a heating block. The reaction mixture was cooled to room temperature and poured carefully in to the ice–water mixture. The aqueous layer was extracted with DCM (3 × 30 mL), the organic extracts were combined, washed with brine and dried over magnesium sulphate. The solvent was removed *in vacuo* and the resulting residue was chromatographed over silica gel (DCM 100%) to yield **110** as a white solid (9 g, 67%). ^1^H NMR (400 MHz, CDCl_3_) *δ* 7.38 (s, 1H), 7.36 (s, 1H), 7.35 (s, 1H), 4.05 (s, 3H), 4.02 (s, 3H); ^13^C NMR (101 MHz, CDCl_3_) *δ* 153.90, 150.83, 147.55, 145.28, 142.08, 120.68, 119.82, 107.76, 101.81, 56.38, 56.28.

#### 4-Chloro-6,7-dimethoxy-2-(4-methylpiperazin-1-yl)quinoline (**111a**)

A mixture of 2,4-dichloro-6,7-dimethoxyquinoline (**110**, 0.774 g, 3 mmol), *N*-methylpiperazine (0.221 mL, 2 mmol) and DIEA (0.36 mL, 2.1 mmol) in THF (15 mL) was heated in a microwave reactor at 120 °C (sealed tube) for 48 hours. The volatiles were removed *in vacuo* and the residue was dissolved in DCM and washed with brine. The organic layer was dried over magnesium sulphate, evaporated *in vacuo* and the residue was purified by silica gel chromatography (DCM : MeOH (7N NH_3_); 98 : 2 → 96 : 4) to yield desired isomer **111a** as a white crystalline solid (111 mg, 17%) together with the minute quantity of the regioisomer (see ESI‡) and unreacted **110** (0.364 g, 47%). ^1^H NMR (400 MHz, CDCl_3_) *δ* 7.26 (s, 1H), 7.10 (s, 1H), 6.94 (s, 1H), 4.00 (s, 3H), 3.99 (s, 3H), 3.72–3.69 (m, 4H), 2.64–2.45 (m, 4H), 2.36 (s, 3H). ^13^C NMR (101 MHz, CDCl_3_) *δ* 156.68, 153.01, 147.38, 145.13, 141.64, 115.70, 107.19, 106.64, 102.44, 56.08, 56.02, 54.94, 46.20, 45.26. HRMS (+ESI) *m*/*z* calcd for C_16_H_21_ClN_2_O_3_, 322.1322, found, 322.1333.

#### 4-Chloro-6,7-dimethoxy-2-(4-methyl-1,4-diazepan-1-yl)quinoline (**111b**)

**111b** was synthesized from **110** (1.161 g, 4.5 mmol), 1-methyl-1,4-diazepane (0.37 mL, 3 mmol) and DIEA (1.09 mL, 6.3 mmol) following a procedure similar to the synthesis of **111a**. After silica gel chromatography **111b** was obtained as a yellow solid (180 mg, 18%), while its regioisomer (ESI‡) was obtained as a white solid (75 mg, 7%). Additionally, 0.741 g (64%) of starting material was also recovered. ^1^H NMR (400 MHz, CDCl_3_) *δ* 7.24 (s, 1H), 7.06 (s, 1H), 6.80 (s, 1H), 3.99 (s, 3H), 3.98 (s, 3H), 3.94–3.92 (m, 2H), 3.73 (t, *J* = 6.4 Hz, 2H), 2.78–2.76 (m, 2H), 2.62–2.60 (m, 2H), 2.41 (s, 3H), 2.08 (dt, *J* = 11.3, 6.0 Hz, 2H). ^13^C NMR (101 MHz, CDCl_3_) *δ* 155.76, 152.95, 146.77, 145.48, 141.45, 114.92, 106.49, 106.14, 102.55, 58.50, 57.32, 56.06, 56.03, 46.74, 46.60, 46.08, 27.46. HRMS (+ESI) *m*/*z* calcd for C_17_H_23_ClN_2_O_3_, 336.1479, found, 336.1471.

#### 4-Chloro-6,7-dimethoxy-2-(4-methoxypiperidin-1-yl)quinoline (**111c**)

**111c** was synthesized from **110** (1.30 g, 5.03 mmol), 4-methoxypiperidine (0.386 mL, 3.36 mmol) and DIEA (1.23 mL, 7.06 mmol) following a procedure similar to the synthesis of **111a**. After silica gel chromatography **111c** was obtained as a yellow solid (310 mg, 27%) ^1^H NMR (500 MHz, CDCl_3_) *δ* 7.24 (s, 1H), 7.09 (s, 1H), 6.95 (s, 1H), 4.10–4.05 (m, 2H), 4.00 (s, 3H), 3.99 (s, 3H), 3.46 (tt, *J* = 8.1, 3.7 Hz, 1H), 3.39 (s, 3H), 3.31 (ddd, *J* = 13.0, 9.3, 3.3 Hz, 2H), 2.03–1.97 (m, 2H), 1.66 (td, *J* = 8.9, 4.1 Hz, 3H). ^13^C NMR (126 MHz, CDCl_3_) *δ* 156.51, 152.93, 147.22, 145.22, 141.58, 115.41, 107.31, 106.60, 102.45, 76.28, 56.06, 56.01, 55.64, 42.97, 30.49. HRMS (+ESI) *m*/*z* calcd for C_17_H_22_ClN_2_O_3_, 337.1319, found, 337.1312.

#### *N*-(1-Benzylpiperidin-4-yl)-6,7-dimethoxy-2-(4-methyl-1,4-diazepan-1-yl)quinolin-4-amine (**88**)

To the mixture of palladium catalyst (PEPPSI-iPr®, CAS number: 905459-27-0) (2.9 mg, 0.004 mmol) and LiOtBu (0.286 mmol, 1 M solution) in anhydrous THF (2 mL), a solution of **111b** (48 mg, 0.143 mmol) and 1-benzyl-4-piperidylamine (54 mg, 0.286 mmol) in THF (3 mL) was added. The reaction mixture was heated in a microwave reactor at 100 °C (sealed tube) for 18 hours. The volatiles were removed *in vacuo* and the residue was dissolved in DCM and washed with brine. The organic layer was dried over magnesium sulphate, evaporated *in vacuo* and the residue was purified by silica gel chromatography (DCM : MeOH (7N NH_3_); 98 : 2 → 90 : 10) to obtain **88** as a yellow solid (13.5 mg, 19%).^1^H NMR (400 MHz, CDCl_3_) *δ* 7.34–7.27 (m, 5H), 7.05 (s, 1H), 6.72 (s, 1H), 5.76 (s, 1H), 4.28 (s, 1H, br), 3.97 (s, 3H), 3.95–3.92 (m, 5 H), 3.75 (t, *J* = 6.3 Hz, 2H), 3.57 (s, 2H), 3.53–3.47 (m, 1H), 2.91 (d, *J* = 11.7 Hz, 2H), 2.75–2.73 (m, 2H), 2.60–2.57 (m, 2H), 2.38 (s, 3H), 2.24 (td, *J* = 11.5, 11.0, 2.7 Hz, 2H), 2.17–2.14 (m, 2H), 2.04 (p, *J* = 5.8 Hz, 2H), 1.70–1.62 (m, 2H). HRMS (+ESI) *m*/*z* calcd for C_29_H_40_N_5_O_2_, 490.3182, found, 490.3197.

#### *N*-(1-Benzylpiperidin-4-yl)-6,7-dimethoxy-2-(4-methylpiperazin-1-yl)quinolin-4-amine (**89**)

**89** was synthesized from **111a** (41 mg, 0.128 mmol) and 1-benzyl-4-piperidylamine (46 mg, 0.243 mmol) following a procedure similar to the synthesis of **88**. After purification **89** was obtained as a yellow solid (25 mg, 41%). ^1^H NMR (400 MHz, CDCl_3_) *δ* 7.39–7.26 (m, 5H), 7.08 (s, 1H), 6.72 (s, 1H), 5.91 (s, 1H), 4.32 (d, *J* = 7.3 Hz, 1H, br), 3.97 (s, 3H), 3.96 (s, 3H), 3.64 (t, *J* = 5.0 Hz, 4H), 3.57 (s, 2H), 3.51 (ddd, *J* = 9.5, 7.7, 4.6 Hz, 1H), 2.91 (d, *J* = 11.8 Hz, 2H), 2.55 (t, *J* = 5.1 Hz, 4H), 2.36 (s, 3H), 2.25 (td, *J* = 11.3, 2.5 Hz, 2H), 2.18–2.12 (m, 2H), 1.72–1.64 (m, 2H). ^13^C NMR (101 MHz, CDCl_3_) *δ* 158.67, 151.85, 148.65, 145.85, 144.94, 138.20, 129.11, 128.23, 127.10, 108.54, 107.71, 99.07, 85.27, 63.08, 56.23, 55.90, 55.20, 52.16, 49.49, 46.25, 45.63, 32.20. HRMS (+ESI) *m*/*z* calcd for C_28_H_38_N_5_O_2_, 476.3026, found, 476.3019.

#### *N*-(1-Benzylpiperidin-4-yl)-6,7-dimethoxy-2-(4-methoxypiperidin-1-yl)quinolin-4-amine (**90**)

**90** was synthesized from **111c** (72 mg, 0.214 mmol) and 1-benzyl-4-piperidylamine (81 mg, 0.428 mmol) following a procedure similar to the synthesis of **88**. After purification **90** was obtained as a yellow solid (26.8 mg, 26%). ^1^H NMR (400 MHz, CDCl_3_) *δ* 7.36–7.25 (m, 5H), 7.09 (s, 1H), 6.73 (s, 1H), 5.93 (s, 1H), 4.32 (s, 1H, br), 4.08 (dt, *J* = 13.4, 4.6 Hz, 2H), 3.97 (s, 3H), 3.96 (s, 3H), 3.57 (s, 2H), 3.55–3.46 (m, 1H), 3.45–3.40 (m, 4H), 3.21 (t, *J* = 11.3 Hz, 2H), 2.93–2.89 (m, 2H), 2.24 (td, *J* = 11.2, 2.5 Hz, 2H), 2.17–2.12 (m, 2H), 2.10–1.99 (m, 2H), 1.70–1.61 (m, 4H). ^13^C NMR (101 MHz, CDCl_3_) *δ* 158.56, 151.82, 148.65, 145.77, 144.95, 138.19, 129.10, 128.22, 127.08, 108.36, 107.60, 99.12, 85.57, 77.31, 77.00, 76.68, 63.07, 56.24, 55.89, 55.56, 52.15, 49.47, 43.61, 32.17, 30.73. HRMS (+ESI) *m*/*z* calcd for C_29_H_38_N_4_O_3_, 491.3022, found, 491.3010.

#### 6,7-Dimethoxy-2-(4-methylpiperazin-1-yl)-*N*-(1-methylpiperidin-4-yl)quinolin-4-amine (**91**)

**91** was synthesized from **111a** (30 mg, 0.093 mmol) and 1-methyl-l-4-piperidylamine (20 mg, 0.175 mmol) following a procedure similar to the synthesis of **88**. After purification **91** was obtained as an off white solid (16.3 mg, 44%). ^1^H NMR (400 MHz, CDCl_3_) *δ* 7.19 (s, 1H), 6.76 (s, 1H), 5.88 (s, 1H), 4.44 (s, 1H, br), 3.98 (s, 3H), 3.97 (s, 3H), 3.66 (t, *J* = 5.1 Hz, 4H), 3.50 (dd, *J* = 9.9, 4.5 Hz, 1H), 2.89 (d, *J* = 11.1 Hz, 2H), 2.57 (t, *J* = 5.0 Hz, 4H), 2.36 (s, 3H), 2.35 (s, 3H), 2.27–2.15 (m, 4H), 1.74–1.65 (m, 2H). HRMS (+ESI) *m*/*z* calcd for C_22_H_34_N_5_O_2_, 400.2713, found, 400.2712.

### Biology procedures

#### *In vitro P. falciparum* growth and proliferation assays

Compounds were tested against drug sensitive *P. falciparum* 3D7 strain parasites using a three-day SYBR Green I based assay. Parasites were cultured at 2% hematocrit with an initial parasitemia of 0.5–0.8% in RPMI 1640 containing 0.5% Albumax. Compounds were initially screened at 2 μM in duplicate wells in a 96-well format. Subsequent IC_50_ values for active compounds were determined with 1 : 1 and 2 : 1 dilutions of test compounds.

#### *In vitro* cytotoxicity assays

Host cell cytotoxicity was determined in a 96-well format with a starting HepG2 cell density of 10 000 cells per well grown in DMEM. Cells were incubated with 1 : 1 dilutions of test compounds for three days and resulting cell viability was quantified using Promega CellTiterBlue.

#### *In vitro* stage-dependent antimalarial activity

Stage-specific compound treatment effects were elucidated using highly synchronized parasites in 48-well plates with a starting parasitemia of 0.75% and a hematocrit of 2%. Parasites were treated for 12 hours with 10× IC_50_ concentrations of compounds **1**, **89** and **90** during three distinct periods of the intraerythrocytic life cycle. After treatment, parasites were washed with warm RPMI 1640 medium and placed back into culture without test compound for analysis of re-invasion after completion of the cell cycle in which treatment occurred. Washed parasites after treatment were also diluted 1 : 16 and allowed to grow without compound for an additional two cell cycles (4 days). Parasitemia after re-invasion or after 4 day growth was quantified on infected cells fixed in 0.025% glutaraldehyde and stained with 2× SYBR Green I (Lonza) in PBS.

#### Parasite histone methylation analysis

Parasites were treated with drugs for 12 h. After treatment, infected red blood cells were collected and lysed with PBS containing 0.15% saponin. Free parasites were lysed by bath sonication in 1% SDS-PBS and analysed by Western Blot. Blots were probed with primary antibodies specific for H3K4me3 (Abcam ab1012 1 : 1000), H3 core (Abcam ab1791, 1 : 3000) and H3K9me3 (Abcam ab8898, 1 : 1000) diluted in TBS-T (50 mM Tris pH 7.5, 150 mM NaCl, 0.05% Tween-20, 5% BSA), followed by anti-mouse HRP (GE NA931V) or anti-rabbit HRP (GE NA934V) secondary antibodies. Blots were revealed using SuperSignal West Pico Chemiluminescent Substrate (Thermo Scientific) and quantified using Bio-Rad Image Lab software.

#### Methyltransferase activity assays

The assay were performed by monitoring the incorporation of tritium labelled methyl group to biotinylated peptide substrates using a scintillation proximity assay (SPA) as described earlier.^40^ Reaction buffer used was 25 mM potassium phosphate, pH 8.0, 1 mM EDTA, 2 mM MgCl_2_, and 0.01% Triton X-100. Assays were performed using 5 nM G9a in a 10 μl reaction mixture containing substrate peptide (H31–25; 0.8 μM) and ^3^H-SAM (8 μM) (Cat.# NET155V250UC; Perkin Elmer; ; www.perkinelmer.com) close to their *K*_m_ values for G9a. Incubation time was 15 minutes and the enzymatic reaction was stopped by adding 10 μL of 7.5 M guanidine hydrochloride, followed by 180 μL of buffer. The reaction was mixed and transferred to a 96-well FlashPlate (Cat.# SMP103; Perkin-Elmer; ; www.perkinelmer.com), incubated for 1 hour and the CPM were measured using Topcount plate reader (Perkin Elmer, ; www.perkinelmer.com). The CPM counts in the absence of compound for each data set was defined as 100% activity. In the absence of the enzyme, the CPM counts in each data set were defined as background (0%).

## Abbreviations

PfHKMT: *P. falciparum* histone lysine methyltransferase
ACTs: Artemisinin-based combination therapies
PfHDAC: *P. falciparum* histone deacetylases
TPSA: Total polar surface area
ICW: In-Cell Western assay
PRC2: Polycomb repressive complex 2
ECSD: Enzyme-coupled SAH detection assay
CLOT: Chemiluminescence-based oxygen tunnelling assay

## Conflict of interest declaration

We wish to confirm that there are no known conflicts of interest associated with this publication and there has been no significant financial support for this work that could have influenced its outcome.

## Author contributions

MF and AS conceptualized and designed the project. SS, ASL and GAL performed chemical synthesis, purification and characterization of compounds. PBC and NAM performed *in vitro* parasite growth/proliferation assay, HepG2 cytotoxicity assay and Western blot analysis. FL performed human G9a inhibition assays. MV and AS contributed to the assay designs and discussions throughout. AJW solved, refined and analyzed X-ray crystal structure of **111a**. SS and MF compiled/analysed all data and drafted the manuscript. All authors participated in revision of the manuscript before submission.

## Supplementary Material

Supplementary information

Crystal structure data
