# Supplementary material for: Histone lysine methyltransferase structure activity relationships that allow for segregation of G9a inhibition and anti-Plasmodium activity
Source: Medchemcomm. 2017 Mar 15;8(5):1069–92. doi: 10.1039/c7md00052a (PMC5708365; doi:10.1039/c7md00052a)
Supplement: Supplementary file 1 [file MD-008-C7MD00052A-s001.pdf]

Electronic Supplementary Material (ESI) for MedChemComm.
This journal is © The Royal Society of Chemistry 2017

# ELECTRONIC SUPPLEMENARY INFORMATION

## Identification of diaminoquinazoline histone lysine methyltransferase structure activity relationships that allow for segregation of human G9a inhibition and anti-*Plasmodium* activity

Sandeep Sundriyal[ǂ], Patty B. Chen[†], Alexandra S. Lubin[ǂ], Gregor A. Lueg[ǂ], Fengling Li[‡], Andrew J. P. White[ǂ], Nicholas A. Malmquist[†], Masoud Vedadi[‡], Artur Scherf[†], Matthew J. Fuchter[ǂ*]

**Contents:**

| | | |
|---|---|---|
| I. | Table ST1 - ST5 | 2-7 |
| II. | Synthesis of Diaminoquinazolines: Representative examples from Table 1 and 2 | 8-13 |
| III. | Synthesis of Diaminoquinazolines: Representative examples from Table 3 | 14-17 |
| IV. | NOE analysis of diaminoisoquinolines | 18 |
| V. | NOE analysis/spectral data for diaminoquinolines intermediates | 19-21 |
| VI. | X-ray crystal structure of **111a** (Figure SF1) | 22 |
| VII. | References | 23 |

# Table ST1

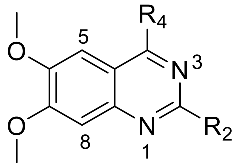

| ID | R4 | R2 | Pf3D7 $IC_{50}$ (nM)[a] | G9a $IC_{50}$ (nM) | G9a/Pf3D7 | HepG2 $IC_{50}$ (μM) | clogP | PSA |
|---|---|---|---|---|---|---|---|---|
| **S1** | 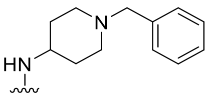 | 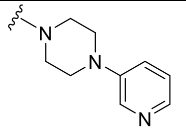 | 67.6 | >1000 | >14.8 | 4.9 | 4.44 | 78.88 |
| **S2** | 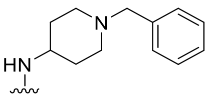 | 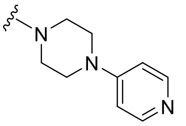 | 54.0 | 338 | 6.3 | 4.9 | 4.44 | 78.88 |
| **S3** | 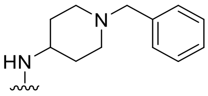 | 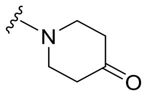 | 30.3 | >1000 | >33 | 10.7 | 3.89 | 79.82 |
| **S4** | 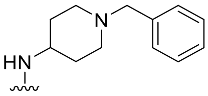 | 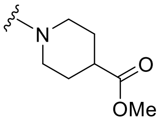 | 41.7 | >1000 | >24 | 9.6 | 4.11 | 89.05 |
| **S5** | 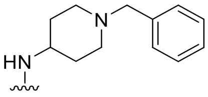 | 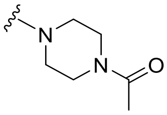 | 47.0 | >1000 | >21.3 | 11.2 | 4.53 | 79.82 |
| **S6** | 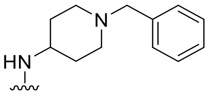 | 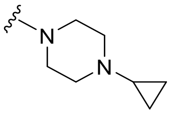 | 45.4 | >1000 | >22 | 7.9 | 5.35 | 62.75 |
| **S7** | 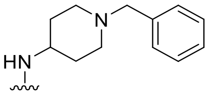 | 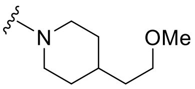 | 80.4 | NT | ND | 6.3 | 4.98 | 71.98 |
| **S8** | 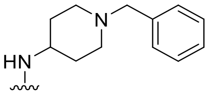 | 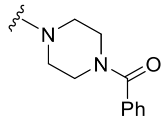 | 60.6 | >1000 | >16.5 | 6.4 | 5.82 | 79.82 |
| **S9** | 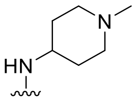 | 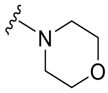 | 173.8 | >1000 | >5.8 | 26.8 | 1.99 | 71.98 |
| **S10** | 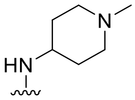 | 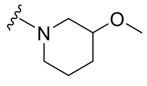 | 53.4 | >1000 | >18.7 | 10.8 | 2.77 | 71.98 |
| **S11** | 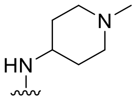 | 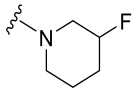 | 19.6 | >1000 | >51 | 11.7 | 3.09 | 62.75 |
| **S12** | 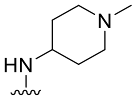 | 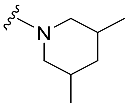 | 18.7 | 185 | 9.9 | 5.7 | 3.64 | 62.75 |
| **S13** | 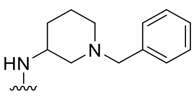 | 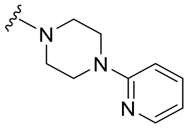 | 144.5 | NT | ND | 6.5 | 4.44 | 78.88 |
| **S14** | 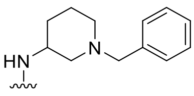 | 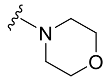 | 445 | >1000 | >2.2 | ND | 3.56 | 71.98 |

| | | | | | | | | |
|---|---|---|---|---|---|---|---|---|
| **S15** | 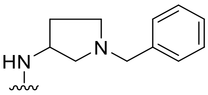 | 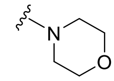 | 152.4 | >1000 | >6.6 | 15.3 | 3.17 | 71.98 |
| **S16** | 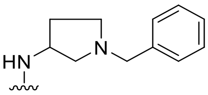 | 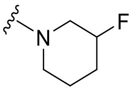 | 110.6 | >1000 | >9 | 6.5 | 4.27 | 62.75 |
| **S17** | 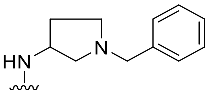 | 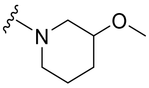 | 111.0 | >1000 | >9 | 10.8 | 3.95 | 71.98 |
| **S18** | 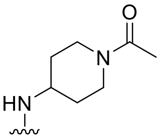 | 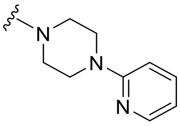 | 335.0 | >1000 | >3 | ND | 2.79 | 95.95 |
| **S19** | 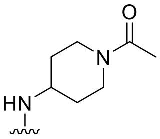 | 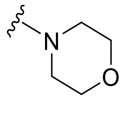 | >2000 | >1000 | ND | ND | 1.91 | 89.05 |
| **S20** | 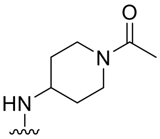 | 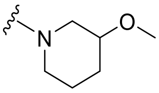 | >2000 | >1000 | ND | ND | 2.68 | 89.05 |
| **S21** | 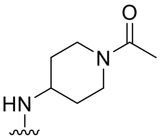 | 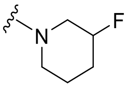 | >2000 | >1000 | ND | ND | 3.01 | 79.82 |
| **S22** | 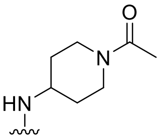 | 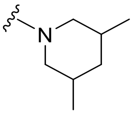 | 107.4 | >1000 | >9.3 | 17.8 | 3.55 | 79.82 |
| **S23** | 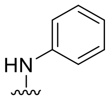 | 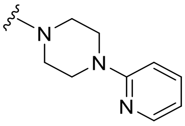 | 194.5 | >1000 | >5.1 | 28.3 | 4.11 | 75.64 |
| **S24** | 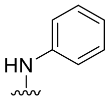 | 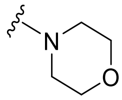 | 922.6 | NT | ND | ND | 3.23 | 68.74 |
| **S25** | 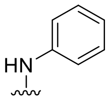 | 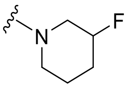 | 272.3 | >1000 | >3.7 | ND | 4.33 | 59.51 |
| **S26** | 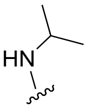 | 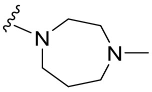 | 322.5 | >1000[b]/ >10000[c] | >3.1/ >31 | ND | 2.61 | 62.75 |
| **S27** | 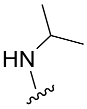 | 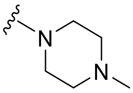 | >300 | NT | ND | ND | 2.22 | 62.75 |
| **S28** | 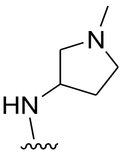 | 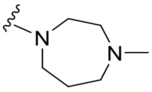 | >300 | 910[b]/ 1900[c] | ND | ND | 1.90 | 65.99 |
| **S29** | 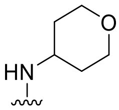 | 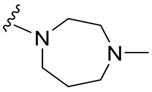 | >300 | >1000[b]/ >10000[c] | ND | ND | 2.38 | 71.98 |

[a] Parasite-killing activity reported earlier[1]
[b] $IC_{50}$ reported using enzyme-coupled SAH detection (ECSD) assay[2, 3]
[c] $IC_{50}$ reported in using chemiluminescence-based oxygen tunnelling (CLOT) assay[2, 3]
NT = not tested; ND = not determined

**Table ST2**

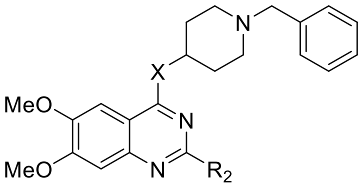


| ID | X | R2 | Pf3D7 $IC_{50}$ (nM)[a] | G9a $IC_{50}$ (nM) | G9a/Pf3D7 | HepG2 $IC_{50}$ (μM) | clogP | PSA |
|---|---|---|---|---|---|---|---|---|
| **S30** | N-Me | 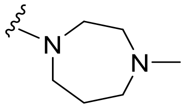 | 158.7 | >10000 | >63 | 6.0 | 3.89 | 57.2 |
| **S31** | N-Me | 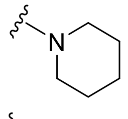 | 495.1 | >10000 | >20.2 | 8.1 | 4.74 | 53.96 |
| **S32** | N-Me | 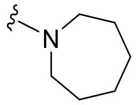 | 399.5 | >50000 | >125.2 | 5.4 | 5.13 | 53.96 |
| **S33** | N-Me | 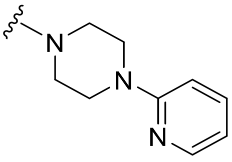 | 472.3 | >50000 | >105.9 | 9.6 | 4.47 | 70.09 |
| **S34** | O | 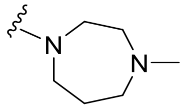 | 1464 | >50000 | >34.1 | 7.9 | 3.83 | 63.19 |
| **S35** | O | 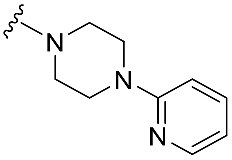 | 2061 | >50000 | >24.3 | 15.7 | 4.41 | 76.08 |
| **S36** | S | 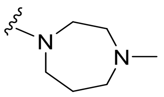 | 1541 | >50000 | >32.4 | 10.2 | 4.55 | 53.96 |
| **S37** | S | 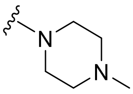 | NT | >50000 | ND | ND | 4.16 | 53.96 |
| **S38** | S | 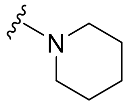 | NT | >50000 | ND | ND | 5.39 | 50.72 |

[a] Parasite-killing activity reported earlier[1]
NT = not tested; ND = not determined

## Table ST3

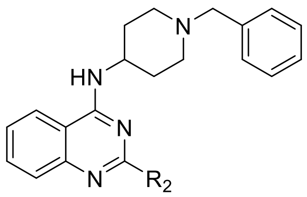


| ID | $R_2$ | Pf3D7 $IC_{50}$ (nM)[a] | G9a $IC_{50}$ (nM) | G9a/Pf3D7 | HepG2 $IC_{50}$ (μM) | clogP | TPSA |
|---|---|---|---|---|---|---|---|
| **S39** | 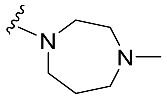 | 100.7 | ~10000 | ~99.3 | 7000 | 3.85 | 47.53 |
| **S40** | 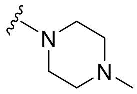 | NT | >10000 | ND | ND | 3.46 | 47.53 |
| **S41** | 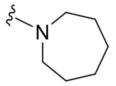 | NT | >50000 | ND | ND | 5.09 | 44.29 |
| **S42** | 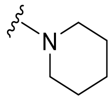 | 384.6 | >50000 | >130 | 4000 | 4.70 | 44.29 |
| **S43** | 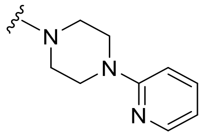 | 353.6 | >50000 | >141.4 | 5000 | 4.43 | 60.42 |

[a] Parasite-killing activity reported earlier[1]
NT = not tested; ND = not determined

# Table ST4

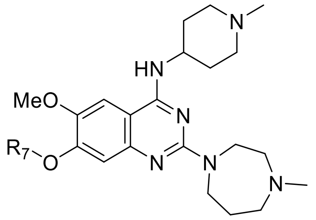


| ID | R7 | Pf3D7 IC50 (nM) [a] | G9a IC50 (nM) | HepG2 IC50 (μM) | clogP | TPSA |
|---|---|---|---|---|---|---|
| **S44** | -OH | >2000 | ~10000 | ND | 1.99 | 76.99 |
| **S45** | 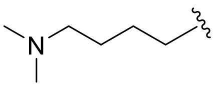 | >2000 | 140[b]/110[c] | ND | 3.01 | 69.23 |
| **S46** | 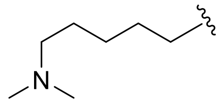 | >2000 | 95[b]/49[c] | ND | 3.40 | 69.23 |
| **S47** | 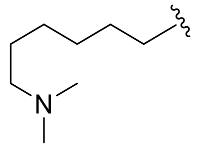 | >2000 | 1500[b]/3200[c] | ND | 3.79 | 69.23 |
| **S48** | 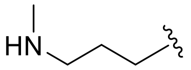 | >2000 | 120[b]/45[c] | ND | 2.28 | 78.02 |
| **S49** | 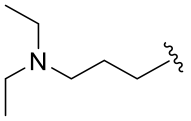 | >2000 | 52[b]/65[c] | ND | 3.40 | 69.23 |
| **S50** | 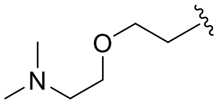 | >2000 | 9[b]/6[c] | ND | 2.24 | 78.46 |
| **S51** | 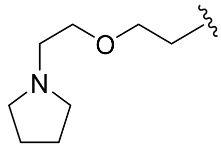 | >2000 | 57[b]/110[c] | ND | 2.78 | 78.46 |
| **S52** | 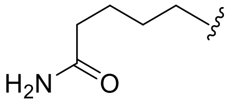 | >2000 | >1000[b]/ >10000[c] | ND | 2.32 | 109.1 |

[a] Parasite-killing activity reported earlier[1]
[b] $IC_{50}$ reported using enzyme-coupled SAH detection (ECSD) assay[2, 3]
[c] $IC_{50}$ reported using chemiluminescence-based oxygen tunnelling (CLOT) assay[2, 3]
ND = not determined

## Table ST5

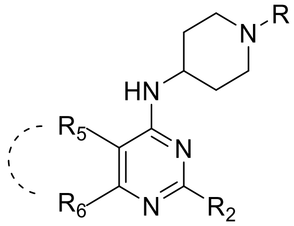


| ID | R | $R_5/R_6$ | $R_2$ | Pf3D7 $IC_{50}$ (nM) | G9a $IC_{50}$ (nM) | G9a/Pf3D7 | HepG2 $IC_{50}$ (nM) | clogP | PSA |
|---|---|---|---|---|---|---|---|---|---|
| **S53** | -Bn | 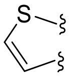 | 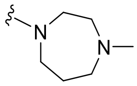 | 562 | ~50000[a] | ~89 | ND | 3.91 | 47.53 |
| **S54** | -Bn | 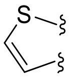 | 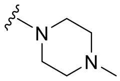 | 1255 | >50000[a] | >39.8 | 8200 | 3.52 | 47.53 |
| **S55** | -Bn | 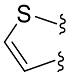 | 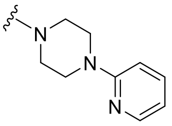 | >2000 | >50000[a] | ND | ND | 4.49 | 60.42 |
| **S56** | -Bn | 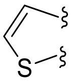 | 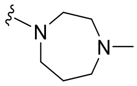 | 1291 | >50000[a] | >38.7 | ND | 3.91 | 47.53 |
| **S57** | -Bn | 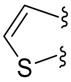 | 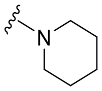 | >2000 | >50000[a] | ND | ND | 4.76 | 44.29 |
| **S58** | -Bn | 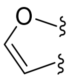 | 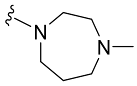 | >2000 | >50000[a] | ND | ND | 3.44 | 60.67 |
| **S59** | -Bn | 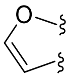 | 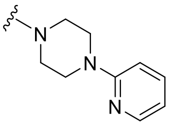 | >2000 | >50000[a] | ND | ND | 4.02 | 73.56 |
| **S60** | -Bn | 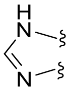 | 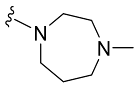 | 1013 | >50000[a] | >49.4 | ND | 2.57 | 76.21 |
| **S61** | -Bn | 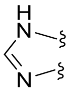 | 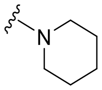 | >2000 | >50000[a] | ND | ND | 3.42 | 72.97 |
| **S62** | -Bn | 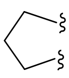 | 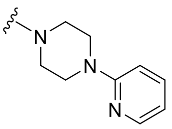 | 1490 | >50000 | >33.6 | ND | 3.76 | 60.42 |
| **S63** | -Bn | 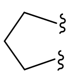 | 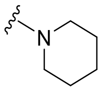 | 1322 | >50000 | >37.8 | ND | 4.03 | 44.29 |
| **S64** | -Me | 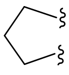 | 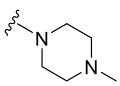 | >2000 | >50000 | ND | ND | 1.22 | 47.53 |

[a] G9a data reported earlier[4]
ND = not determined

## **Synthesis of Diaminoquinazolines:** Representative examples from Table 1 and 2

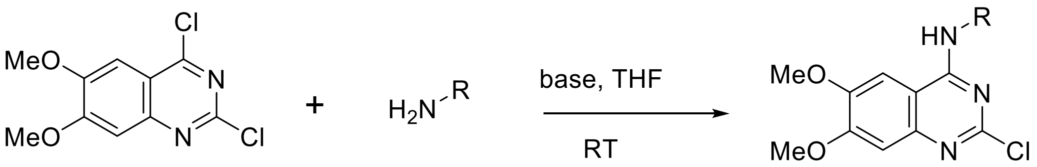


6,7-dimethoxy-2,4-dichloroquinazoline

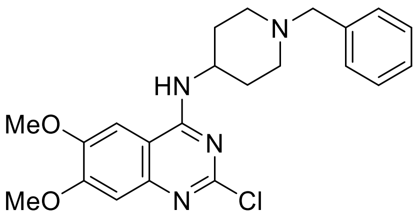

**DQ1**

A solution of 1-benzylpiperidin-4-amine (0.810 g, 4 mmol) 2,4-dichloro-6,7-dimethoxyquinazoline (1.04 g, 4 mmol), and triethylamine (1.39 mL, 10 mmol) in dry THF (15 mL) was allowed to stir at room temperature for 20 h. The reaction mixture was concentrated to obtain a dark residue that was purified by silica gel column chromatography (DCM : MeOH (7 N $NH_3$); 100 : 0 → 97 : 3). The title product was obtained as off-white solid (1.31 g, 79 %). $^1$H NMR (400 MHz, $CDCl_3$) δ 7.30-7.23 (m, 5H), 7.09 (s, 1H), 6.83 (s, 1H), 5.57 (d, $J$ = 7.8 Hz, 1H), 4.31-4.23 (m, 1H), 3.94 (s, 3H), 3.93 (s, 3H), 3.53 (s, 2H), 2.90-2.87 (m, 2H), 2.24-2.10 (m, 4H), 1.66-1.57 (m, 2H); HRMS (+ESI) m/z calcd for $C_{22}H_{26}N_4O_2Cl$ 413.1744, found, 413.1754.

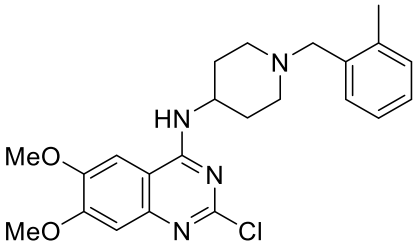

**DQ2**

**N-alkylation:** To a solution of 4-Piperidinecarboxamide (0.640 g, 5 mmol) in ethanol (12 mL), potassium carbonate (1.38 g, 10 mmol) and 2-methyl benzylbromide (0.669 mL, 5 mmol) was added and the reaction mixture was refluxed for 3 h. After cooling to room temperature, the reaction mixture was filtered and ethanol was evaporated. The residue was dissolved in DCM, washed with water and dried over anhydrous magnesium sulphate. The organic layer was removed *in vacuo* to obtain pure 1-(2-methylbenzyl)piperidine-4-carboxamide as white solid (0.898 g, 77 %). $^1$H NMR (400 MHz, $CDCl_3$) δ 7.25-7.11 (m, 4H), 5.65 (s, 1H, br), 5.51 (s, 1H, br), 3.43 (s, 2H), 2.96-2.91 (m, 2H), 2.37 (s, 3H), 2.18-2.12 (m, 1H), 2.04-1.98 (m, 2H), 1.86-1.82 (m, 2H), 1.77-1.67 (m, 2H); HRMS (+ESI) m/z calcd for $C_{14}H_{21}N_2O$ 233.1654, found, 233.1669.

**Hofmann rearrangement:** To a solution of 1-(2-methylbenzyl)piperidine-4-carboxamide (see above, 0.863 g, 3.72 mmol) in acetonitrile (11 mL) and water (7 mL), phenylbis(trifluoroacetato)iodine (PIFA) (1.919 g, 4.464 mmol) was added and the reaction mixture was heated at 65 °C for 18 h. The reaction mixture was concentrated to half volume followed by addition of water and pH adjusted to 1 using 1 M HCl. The aqueous layer was then extracted with ether (25 mL x 2) and the organic layer was discarded. The aqueous layer was then basified using 3 M NaOH to pH 11 and extracted using DCM (15 mL x 3). The organic extracts were combined, dried over anhydrous magnesium sulphate and

removed *in vacuo* to obtain 1-(2-methylbenzyl)piperidin-4-amine as brown oil (0.605 g, 80 %) that was used as such in the next step. $^1$H NMR (400 MHz, $CDCl_3$) δ 7.25-7.24 (m, 1H), 7.15-7.13 (m, 3H), 3.42 (s, 2H), 2.83-2.79 (m, 2H), 2.70-2.63 (m, 1H), 2.35 (s, 3H), 2.06-2.02 (m, 2H), 1.78-1.75 (m, 2H), 1.40-1.31 (m, 4H); HRMS (+ESI) m/z calcd for $C_{13}H_{21}N_2$ 205.1705, found, 205.1692.

**DQ2** was synthesized from 1-(2-methylbenzyl)piperidin-4-amine (0.588 g, 2.88 mmol), 2,4-Dichloro-6,7-dimethoxyquinazoline (0.596 g, 2.30 mmol) and triethylamine (0.641 mL, 4.61 mmol), following procedure similar to the synthesis of **DQ1**. After silica gel chromatography **DQ2** was obtained as an off-white solid (0.957 g, 78 %). $^1$H NMR (400 MHz, $CDCl_3$) δ 7.28-7.26 (m, 1H), 7.17-7.13 (m, 4H), 6.77 (s, 1H), 5.32 (d, *J* = 7.8 Hz, 1H), 4.34-4.25 (m, 1H), 3.99 (s, 3H), 3.97 (s, 3H), 3.49 (s, 2H), 2.90-2.87 (m, 2H), 2.38 (s, 3H), 2.28-2.22 (m, 2H), 2.13-2.10 (m, 2H), 1.67-1.54 (m, 2H); HRMS (+ESI) m/z calcd for $C_{23}H_{28}N_4O_2Cl$ 427.1901, found, 427.1916.

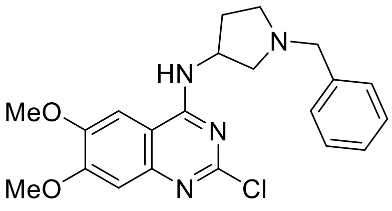


**DQ3**

**DQ3** was synthesized from 1-benzylpyrrolidin-3-amine (0.340 mL, 2 mmol) and 2,4-Dichloro-6,7-dimethoxyquinazoline (0.518 g, 2 mmol) following the procedure similar to the synthesis of **DQ1**. The reaction mixture was stirred for 24 h. The title compound was obtained as a white solid (0.756 g, 95 %). $^1$H NMR (400 MHz, $CDCl_3$) δ 7.35-7.28 (m, 5H), 7.11 (s, 1H), 6.84 (s, 1H,), 6.03 (s, 1H, br), 4.95-4.89 (m, 1H), 3.99 (s, 3H), 3.95 (s, 3H), 3.66 (s, 2H), 3.02 (td, *J* = 8.8, 3.2 Hz, 1H), 2.87-2.81 (m, 1H), 2.66 (dd, *J* = 10.1, 6.3 Hz, 1H), 2.51-2.43 (m, 1H), 2.32 (q, *J* = 8.6 Hz, 1H), 1.80 (dtd, *J* = 12.1, 8.1, 3.7 Hz, 1H); HRMS (+ESI) m/z calcd for $C_{22}H_{26}N_4O_2Cl$ 413.1744, found, 413.1738.

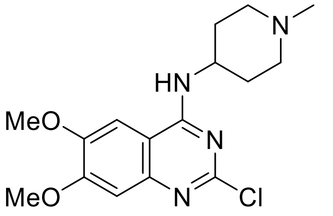


**DQ4**

**DQ4** was synthesized from 1-methylpiperidin-4-amine (0.624 mL, 5 mmol) and 2,4-Dichloro-6,7-dimethoxyquinazoline (1.295 g, 5 mmol) following a procedure similar to the synthesis of **DQ1**. The title compound was obtained as white solid (1.29 g, 77 %). $^1$H NMR (400 MHz, $CDCl_3$) 7.18 (s, 1H), 7.05 (s, 1H), 6.32 (s, 1H, br), 4.31-4.23 (m, 1H), 3.93 (s, 3H), 3.90 (s, 3H), 2.90-2.87 (m, 2H), 2.35-2.25 (m, 5H), 2.14-2.11 (m, 2H), 1.88 (tt, *J* = 12.0, 6.1 Hz, 2H); HRMS (+ESI) m/z calcd for $C_{16}H_{22}N_4O_2Cl$ 337.1431, found, 337.1445.

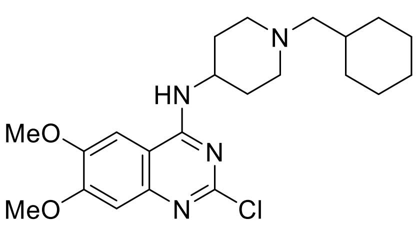


**DQ5**

**Reductive amination:** A solution of 4-(N-Boc-amino)piperidine (0.901 g, 4.5 mmol), and cyclohexanecarboxaldehyde (0.36 mL, 3 mmol) was allowed to stir in 1,2-dichloroethane for 3 h after which sodium triacetoxyborohydride (0.890 g, 4.2 mmol) was added in two portions at half-hour intervals, and the solution was left to stir for 18 h. The reaction mixture was quenched using saturated sodium bicarbonate solution and extracted with ethyl acetate. The combined organic layers were dried over magnesium sulfate, concentrated *in vacuo* and purified by column chromatography (DCM

: MeOH (7 N $NH_3$); 95 : 5) to obtain *tert*-butyl (1-(cyclohexylmethyl)piperidin-4-yl)carbamate as a white solid (0.856 g, 96 %). $^1$H NMR (400 MHz, Chloroform-d) δ 4.43 (s, 1H), 3.47 (s, 1H), 2.78 (d, *J* = 11.6 Hz, 2H), 2.10 (d, *J* = 7.1 Hz, 2H), 2.06-1.96 (m, 2H), 1.95-1.86 (m, 2H), 1.82-1.57 (m, 5H), 1.47 (s, 9H), 1.45-1.11 (m, 6H), 1.00-0.78 (m, 2H); HRMS (+ESI) m/z calcd for $C_{17}H_{33}N_2O$ 297.2542, found 297.2542.

**Boc deprotection:** *tert*-butyl (1-(cyclohexylmethyl)piperidin-4-yl)carbamate was treated with TFA (50 % in DCM) for 4 h, neutralized with sodium hydroxide (2 M) and extracted with DCM. The organic layers were combined, dried over magnesium sulphate, concentrated *in vacuo* to obtain 1-(cyclohexylmethyl)piperidin-4-amine as brown oil (0.513 g, 97 %) that was used as such in next step. $^1$H NMR (400 MHz, Chloroform-d) δ: 2.91-2.78 (m, 2H), 2.67 (tt, *J* = 10.4, 4.2 Hz, 1H), 2.14 (d, *J* = 7.0 Hz, 2H), 2.05-1.92 (m, 2H), 1.87-1.63 (m, 8H), 1.56-1.34 (m, 3H), 1.32-1.11 (m, 2H), 0.96-0.79 (m, 2H); HRMS (+ESI) m/z calcd for $C_{12}H_{25}N_2$ 197.2018 found 197.2022.

**DQ5 synthesis:** 1-(cyclohexylmethyl)piperidin-4-amine (0.420 g, 2.14 mmol) was treated with 2,4-dichloro-6,7-dimethoxyquinazoline (0.554 g, 2.14 mmol) and DIEA (1.15 mL, 6.6 mmol) following procedure similar to the preparation of **DQ1**. The crude product was purified by flash column (DCM : MeOH (7 N $NH_3$); 100 : 0 → 98 : 2) to yield **DQ5** as a pale yellow solid (0.512 g, 41 %). $^1$H NMR (400 MHz, Chloroform-d) δ 7.17 (s, 1H), 6.77 (s, 1H), 5.29 (d, J = 7.6 Hz, 1H), 4.32 (s, 1H), 4.04 (s, 3H), 4.01 (s, 3H), 2.98-2.86 (m, 2H), 2.29-2.12 (m, 6H), 1.89-1.63 (m, 7H), 1.36-1.16 (m, 4H), 1.01-0.84 (m, 2H); $^{13}$C NMR (101 MHz, $CDCl_3$) δ 159.06, 156.37, 154.94, 149.01, 148.08, 109.98, 107.40, 99.46, 65.57, 56.38, 56.28, 52.88, 48.26, 35.32, 32.13, 31.96, 26.76, 26.15; HRMS (+ESI) m/z calcd for $C_{22}H_{32}N_4O_2Cl$ 419.2214, found 419.2214.

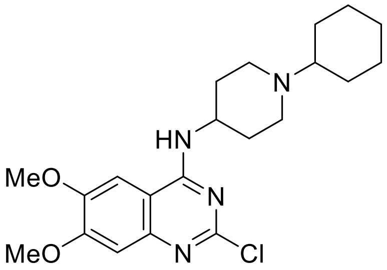


**DQ6**

**DQ6** was synthesized from 1-cyclohexylpiperidin-4-amine (0.225 g, 1.24 mmol) and 2,4-dichloro-6,7-dimethoxyquinazoline (0.321 g, 1.24 mmol) following procedure similar to the preparation of **DQ5**. The crude product was purified by flash column chromatography (DCM : MeOH (7 N $NH_3$); 100 : 0 → 95 : 5) to yield **DQ6** as a pale yellow solid (0.237 g, 47 %). $^1$H NMR (400 MHz, Chloroform-d) δ 7.16 (s, 1H), 6.80 (s, 1H), 5.36(s, 1H), 4.34 (s, 1H), 4.04 (s, 3H), 4.00 (s, 3H), 3.06 (s, 2H), 2.59 (s, 2H), 2.22 (d, J = 15.2 Hz, 1H), 2.00-1.94 (m, 2H), 1.90-1.82 (m, 6H), 1.29 (d, J = 12.2 Hz, 6H); $^{13}$C NMR (101 MHz, $CDCl_3$) δ 159.09, 156.15, 154.96, 149.10, 148.08, 107.32, 106.71, 99.73, 64.19, 56.48, 56.27, 48.16, 48.02, 32.02, 28.55, 26.14, 25.90; HRMS (+ESI) m/z calcd for $C_{21}H_{30}N_4O_2Cl$ 405.2057, found, 405.2054.

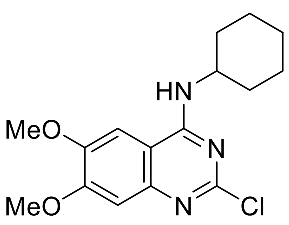


**DQ7**

**DQ7** was synthesized from cyclohexylamine (0.46 mL, 4 mmol) and 2,4-Dichloro-6,7-dimethoxyquinazoline (1.04 g, 4 mmol) following a procedure similar to the preparation of **DQ1**. The final product was obtained as pale a yellow syrup (0.887 g, 69 %). $^1$H NMR (400 MHz, $CDCl_3$) δ 7.08 (s, 1H), 6.87 (s, 1H), 5.56 (s, 1H, br), 4.28-4.21 (m, 1H), 3.95 (s,

3H), 3.93 (s, 3H), 3.53 (s, 2H), 2.14-2.10 (m, 2H), 1.79-1.66 (m, 3H), 1.31-1.16 (m, 3H); HRMS (+ESI) m/z calcd for $C_{16}H_{21}N_3O_2Cl$ 322.1322, found, 322.1316.

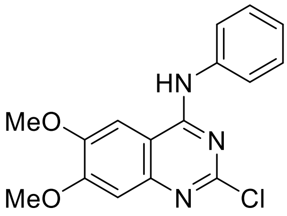

**DQ8**

**DQ7** was synthesized from aniline (0.456 mL, 5 mmol) and 2,4-Dichloro-6,7-dimethoxyquinazoline (1.295 g, 5 mmol) following a procedure similar to the synthesis of **DQ1**. The reaction mixture was stirred for 2.5 days and purified by silica gel chromatography (EtOAc : Pet. ether; 1 : 2). The title compound was obtained as a white solid (0.217 g, 14 %). $^1$H NMR (400 MHz, $CDCl_3$) 7.69 (d, 2H, $J$ = 8.8 Hz, 1H), 7.41 (t, 2H, $J$ = 8.4 Hz), 7.32 (s, 1H, br), 7.20-7.16 (m, 2H), 6.97 (s, 1H), 3.98 (s, 6H); HRMS (+ESI) m/z calcd for $C_{16}H_{15}N_3O_2Cl$ 316.0853, found, 316.0863.

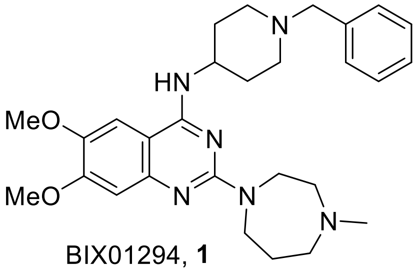

BIX01294, **1**

A mixture of **DQ1** (0.413 g, 1 mmol) and 1-methyl-1,4-diazepane (0.6 mL, 5 mmol) in 2 mL toluene was heated at 130 $^o$C for 50 min under microwave conditions. The reaction mixture was concentrated and purified by silica gel column chromatography (DCM : MeOH (7 N $NH_3$); 98 : 2 → 95 : 5) to yield **1** as light yellow solid (0.489 g, 79 %). $^1$H NMR (400 MHz, $CDCl_3$) δ 7.34-7.23 (m, 5H), 6.89 (s, 1H), 6.69 (s, 1H), 4.96 (d, $J$ = 7.8 Hz, 1H), 4.15-4.06 (m, 1H), 3.99-3.96 (m, 2H), 3.95 (s, 3H), 3.92 (s, 3H), 3.88 (t, $J$ = 6.4 Hz, 2H), 3.55 (s, 2H), 2.92-2.89 (m, 2H), 2.71-2.69 (m, 2H), 2.58-2.56 (m, 2H), 2.37 (s, 3H), 2.22-2.13 (m, 4H), 2.04-1.98 (m, 2H), 1.62 (qd, $J$ = 11.6, 11.0, 4.0 Hz, 2H); $^{13}$C NMR (101 MHz, $CDCl_3$) δ 158.04, 154.40, 145.17, 138.44, 129.11, 128.25, 127.07, 109.99, 105.81, 102.75, 100.82, 63.13, 58.84, 57.26, 56.40, 56.04, 52.57, 48.49, 46.60, 45.88, 45.66, 32.24, 27.59. HRMS (+ESI) m/z calcd for $C_{28}H_{39}N_6O_2$ 491.3134, found, 491.3124.

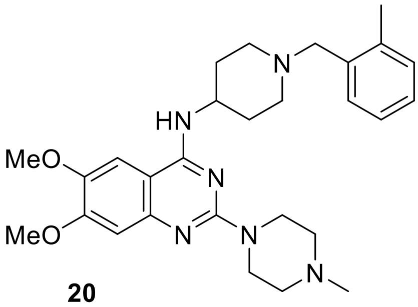

**20**

**20** was synthesized from **DQ2** (0.168g, 0.393 mmol) and N-methyl piperazine (0.435 mL, 3.93 mmol) following a procedure similar to the synthesis of BIX01294 (**1**). After silica gel chromatography it was obtained as a brown solid (0.141 g, 72 %). $^1$H NMR (400 MHz, $CDCl_3$) δ 7.29-7.27 (m, 1H), 7.18-7.14 (m, 3H), 6.90 (s, 1H), 6.68 (s, 1H), 4.97 (d, $J$ = 7.0 Hz, 1H), 4.19-4.12 (m, 1H), 3.95 (s, 3H), 3.94 (s, 3H), 3.88-3.85 (m, 4H), 3.50 (s, 2H), 2.91-2.88 (m, 2H), 2.50 (t, $J$ = 5.0 Hz, 4H), 2.39 (s, 3H), 2.35 (s, 3H), 2.25-2.20 (m, 2H), 2.15-2.12 (m, 2H), 1.63-1.55 (m, 2H); HRMS (+ESI) m/z calcd for $C_{28}H_{39}N_6O_2$ 491.3134, found, 491.3134.

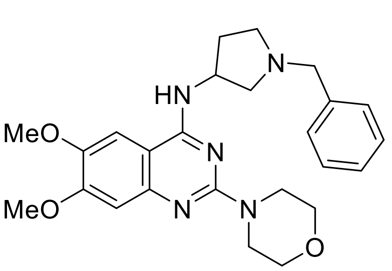

**S15** (Table ST1)

**S15** was synthesized from morpholine (0.215 mL, 2.51 mmol) and **DQ3** (0.20 g, 0.501 mmol) following a procedure similar to the synthesis of **1**. After silica gel chromatography **S15** was obtained as a yellow solid (0.220 g, 98 %). $^{1}H$ NMR (400 MHz, $CDCl_3$) δ 7.35-7.26 (m, 5H), 6.90 (s, 1H), 6.75 (s, 1H,), 5.52 (s, 1H, br), 4.84-4.77 (m, 1H), 3.95 (s, 3H), 3.94 (s, 3H), 3.82-3.76 (m, 8H), 3.67 (s, 2H), 2.95-2.91 (m, 1H), 2.82-2.73 (m, 2H), 2.47-2.41 (m, 2H), 1.80-1.77 (m, 1H); HRMS (+ESI) m/z calcd for $C_{25}H_{32}N_5O_3$ 450.2505, found, 450.2515.

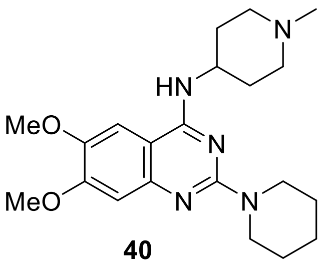

**40**

**40** was synthesized from piperidine (0.66 mL, 6.74 mmol) and **DQ4** (0.227 g, 0.674 mmol) following a procedure similar to the synthesis of **1**. After silica gel chromatography **40** was obtained as a green solid (84 mg, 32 %). $^{1}H$ NMR (400 MHz, $CDCl_3$) 6.90 (s, 1H), 6.70 (s, 1H), 4.94 (d, $J$ = 6.7 Hz, 1H, br), 4.18-4.08 (m, 1H), 3.95 (s, 3H), 3.94 (s, 3H), 3.81-3.77 (m, 4H), 2.89-2.86 (m, 2H), 2.33 (s, 3H), 2.22-2.16 (m, 4H), 1.67-1.58 (m, 8H); HRMS (+ESI) m/z calcd for $C_{21}H_{32}N_5O_2$ 386.2556, found, 386.2534.

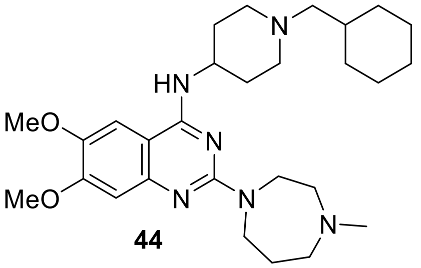

**44**

**44** was synthesized from **DQ5** (30 mg, 0.072 mmol) and 1-methylhomopiperazine (0.03 mL, 0.24 mmol) following a procedure similar to the synthesis of **1**. After silica gel chromatography the product was obtained as a yellow solid (23 mg, 64 %). $^{1}H$ NMR (400 MHz, Chloroform-d) δ 6.91 (s, 1H), 6.71 (s, 1H), 4.96 (d, J = 7.2 Hz, 1H), 4.18-4.03 (m, 1H), 4.02-3.99 (m, 2H), 3.98 (s, 3H), 3.96 (s, 3H), 3.90 (t, J = 6.4 Hz, 2H), 2.90 (d, J = 11.7 Hz, 2H), 2.79-2.70 (m, 2H), 2.64-2.56 (m, 2H), 2.40 (s, 3H), 2.22-1.96 (m, 8H), 1.86-1.57 (m, 6H), 1.58-1.45 (m Hz, 1H), 1.34-1.15 (m, 4H), 0.99-0.83 (m, 2H); $^{13}C$ NMR (101 MHz, $CDCl_3$) δ 158.52, 158.03, 154.32, 149.37, 145.04, 105.99, 102.77, 100.69, 65.78, 58.94, 57.34, 56.36, 56.02, 53.19, 48.53, 46.70, 45.88, 45.73, 35.41, 32.28, 32.00, 27.78, 26.82, 26.20; HRMS (+ESI) m/z calcd for $C_{28}H_{45}N_6O_3$ 497.3604, found, 497.3593.

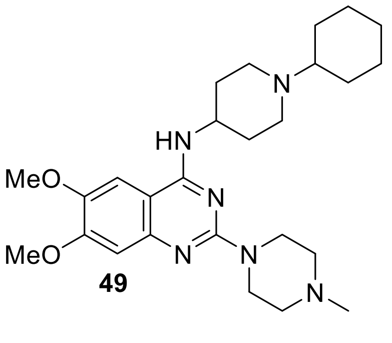

**49**

**49** was synthesized from **DQ6** (30 mg, 0.074 mmol) and 1-methylpiperazine (0.025 mL, 0.23 mmol) following a procedure similar to the synthesis of **1**. The crude product was purified by flash column chromatography to yield the product as a pale yellow solid (17 mg, 49 %). $^{1}$H NMR (400 MHz, Chloroform-d) δ 6.93 (s, 1H), 6.71 (s, 1H), 5.00 (d, J = 7.3 Hz, 1H), 4.19-4.06 (m, 1H), 3.98 (s, 3H), 3.97 (s, 3H), 3.89 (t, J = 5.1 Hz, 4H), 3.07-2.92 (m, 2H,), 2.52 (t, J = 5.1 Hz, 4H), 2.49-2.41 (m, 1H), 2.38 (s, 3H), 2.27-2.15 (m, 2H), 1.99-1.88 (m, 2H), 1.88-1.77 (m, 4H), 1.74-1.54 (m, 2H), 1.37-1.22 (m, 6H); $^{13}$C NMR (101 MHz, CDCl3) δ 158.92, 158.20, 154.35, 149.20, 145.46, 106.13, 103.16, 100.57, 63.86, 56.33, 56.00, 55.27, 48.56, 48.20, 46.35, 44.02, 32.67, 28.89, 26.36, 26.06; HRMS (+ESI) m/z calcd for $C_{26}H_{41}N_6O_2$ 469.3291, found, 469.3293.

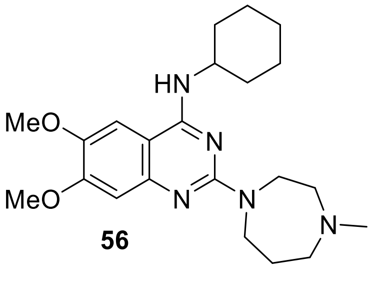


**56**

**56** was synthesizxed from **DQ7** (48 mg, 0.149 mmol) and 1-methyl-1,4-diazepane (0.18 mL, 1.49 mmol) following a procedure similar to the synthesis of **1**. After purification the product was obtained as a yellow solid (54 mg, 91 %). $^{1}$H NMR (400 MHz, $CDCl_3$) 6.88 (s, 1H), 6.69 (s, 1H), 4.93 (d, 1H, br), 4.11-4.05 (m, 1H), 4.00-3.97 (m, 2H ), 3.96 (s, 3H), 3.93 (s, 3H), 3.88 (t, *J* = 6.4 Hz, 2H), 2.72-2.70 (m, 2H), 2.59-2.56 (m, 2H), 2.38 (s, 3H), 2.18-2.14 (m, 2H), 2.02 (dt, *J* = 11.2, 6.2 Hz, 2H), 1.81 (dd, *J* = 11.4, 5.2 Hz, 2H), 1.71-1.67 (m, 1H), 1.49-1.39 (m, 2H), 1.34-1.24 (m, 3H); HRMS (+ESI) m/z calcd for $C_{22}H_{34}N_5O_2$ 400.2713, found, 400.2724.

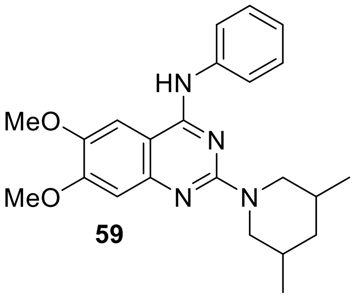


**59**

**59** was synthesized from 3,5-dimethylpiperidine (0.441 mL, 3.32 mmol) and **DQ8** (0.210 g, 0.665 mmol) following a procedure similar to the synthesis of **1**. After silica gel chromatography **59** was obtained as a white solid (81 mg, 31 %). $^{1}$H NMR (400 MHz, $CDCl_3$) δ 7.69 (d, *J* = 7.9 Hz, 2H), 7.38 (t, *J* = 7.9 Hz, 2H), 7.11 (t, *J* = 7.9 Hz, 1H), 6.99 (s, 1H, br), 6.95 (s, 1H), 6.88 (s, 1H), 4.82-4.79 (m, 2H), 3.97 (s, 3H), 3.95 (s, 3H), 2.32 (t, *J* = 12.1 Hz, 2H), 1.82 (d, *J* = 12.7 Hz, 1H), 1.71-1.60 (m, 2H), 0.95 (s, 3H), 0.94 (s, 3H), 0.77 (q, *J* = 12.0 Hz, 1H); HRMS (+ESI) m/z calcd for $C_{23}H_{29}N_4O_2$ 393.2279, found, 393.2291.

**Synthesis of Diaminoquinazolines:** Representative examples from Table 3

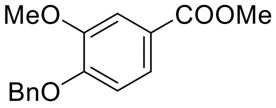

**93**

To an ice-cold mixture of 4-hydroxy-3-methoxybenzoic acid methyl ester **92** (10.09 g, 55.51 mmol) and potassium carbonate (19.07 g, 138 mmol) in DMF (50 mL), benzyl bromide (7.5 mL, 63.1 mmol) was slowly added. The reaction mixture was allowed to stir overnight at room temperature and then poured into an ice-cold saturated aqueous NaCl solution. The precipitate was filtered, washed with distilled water and dried *in vacuo* to afford the title compound **93** as a white solid (15.0 g, 99 %). $^1$H NMR (400 MHz, $CDCl_3$) δ 7.64-7.54 (m, 2H), 7.46-7.28 (m, 5H), 6.89 (d, J = 8.4 Hz, 1H), 5.22 (s, 2H), 3.94 (s, 3H), 3.88 (s, 3H).

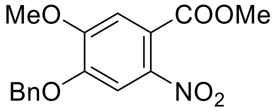

**94**

To a solution of **93** (14.32 g, 52.6 mmol) in acetic anhydride (140 mL), nitric acid (conc. 67 %, 12.6 mL) was slowly added and the reaction mixture was allowed to stir for 18 h at room temperature. The reaction mixture was poured into ice water and the precipitate was filtered, washed with distilled water and dried *in vacuo* to afford **94** as a yellow solid (13.7 g, 89 %). $^1$H NMR (400 MHz, $CDCl_3$) δ 7.52 (s, 1H), 7.46-7.31 (m, 5H), 7.08 (s, 1H), 5.21 (s, 2H), 3.98 (s, 3H), 3.91 (s, 3H).

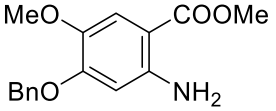

**95**

To the mixture of **94** (11.00 g, 34.7 mmol) and Fe dust (7.25 g, 130 mmol) in 160 mL water and *i*PrOH (5 : 3), ammonium chloride (10.51 g, 196 mmol) was added and the reaction mixture was refluxed for 18 h. The resulting precipitate was filtered and washed with 250 mL DCM (with 10 % MeOH). The filtrate was dried over $Na_2SO_4$ and volatiles were removed *in vacuo* to afford **95** as a white solid (8.38 g, 84 %). $^1$H NMR (400 MHz, $CDCl_3$) δ 7.45-7.30 (m, 6H), 6.26 (s, 1H), 5.15 (s, 2H), 3.85 (s, 3H), 3.84 (s, 3H); HRMS (+ESI) m/z calcd for $C_{16}H_{17}NO_4$ 288.1236, found, 288.1233.

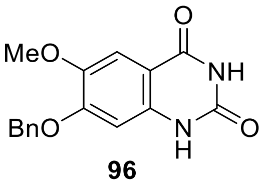

**96**

To the solution of **95** (8.35 g, 29.1 mmol) in 60 mL AcOH and $H_2O$ (2 : 1), 4.76 g of NaOCN was added and the reaction mixture was allowed to stir for 18 h at room temperature. Afterwards, 60 mL of MeOH was added, the reaction mixture was basified with 8N NaOH solution to pH 13, and refluxed for another 6 h. The reaction mixture was allowed to cool to room temperature and neutralised with concentrated HCl to obtain precipitate that was filtered, washed with water and dried *in vacuo* to afford **96** as a brown solid (6.35 g, 73 %). $^1$H NMR (400 MHz, DMSO-d6) δ 7.49-7.34 (m, 5H), 7.27 (s, 1H), 6.78 (s, 1H), 5.14 (s, 1H), 3.79 (s, 3H); HRMS (+ESI) m/z calcd for $C_{16}H_{15}N_2O_4$ 299.1032, found, 299.1029.

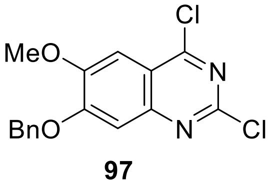

**97**

A mixture of **96** (6.35 g, 21.3 mmol), DIEA (3.6 mL, 39.1 mmol) and phosphorus oxychloride (20.3 mL, 79.5 mmol) in acetonitrile (115 mL) was refluxed for 6 h. The reaction mixture was concentrated *in vacuo* to about 15 mL and

poured in an ice-cold saturated $NaHCO_3$ solution to obtain precipitate that was filtered, washed with water and dried *in vacuo* to afford **97** as a brownish powder (6.92 g, 97 %). $^1$H NMR (400 MHz, DMSO-d6) δ 7.56 (s, 1H), 7.53-7.35 (m, 5H), 5.37 (s, 2H), 4.00 (s, 3H); $^{13}$C NMR (101 MHz, DMSO-d6) δ 159.75, 157.20, 152.21, 152.18, 150.44, 135.99, 129.08, 128.87, 128.73, 117.86, 107.87, 103.15, 71.36, 56,85; HRMS (+ESI) m/z calcd for $C_{16}H_{13}N_2O_2Cl_2$ 335.0354, found, 335.0358 .

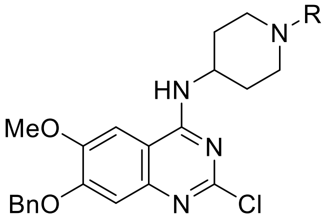


**98**
R = Bn

A mixture of **97** (1.96 g, 5.85 mmol), 1-benzylpiperidin-4-amine (1.79 mL, 8.78 mmol) and DIEA (2.03 mL, 11.7 mmol) was allowed to stir in THF for 20 h. The solvent was removed and the residue was partitioned in water and DCM. The organic extraacts were combined, dried over magnesium sulphate and volatiles removed *in vacuo* to obtain solid residue that was purified by silica gel chromatography (DCM : MeOH (7 N $NH_3$); 99 : 1 → 97 : 3) to yield **98** (R = Bn) as a pale white solid (1.20 g, 42 %). $^1$H NMR (400 MHz, Chloroform-d) δ 7.51-7.24 (m, 10H), 7.14 (s, 1H), 6.81 (s, 1H), 5.42 (d, *J* = 7.8 Hz, 1H), 5.23 (s, 2H), 4.32-4.23 (m, 1H), 3.97 (s, 3H), 3.55 (s, 2H), 2.90 (dt, *J* = 12.4, 3.7 Hz, 2H), 2.26-2.20 (m, 2H), 2.11 (dd, *J* = 11.8, 3.9 Hz, 2H), 1.67-1.57 (m, 2H); HRMS (+ESI) m/z calcd for $C_{28}H_{30}N_4O_2Cl$ 489.2075, found, 489.2044.

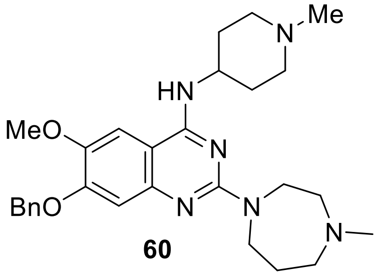


**60**

**60** was synthesized from **98** (R = Me) (0.369 g, 0.871 mmol and 1-methyl-1,4-diazepane (0.540 mL, 4.355 mmol) following a procedure similar to the synthesis of **1**. After silica gel chromatography **60** was obtained as a white solid (0.239 g, 66 %). $^1$H NMR (400 MHz, $CDCl_3$) δ 7.48 (d, *J* = 7.1 Hz, 2H), 7.39 (t, *J* = 7.8 Hz, 2H), 7.34-7.30 (m, 1H), 6.97 (s, 1H), 6.78 (s, 1H), 5.23 (s, 2H), 5.01 (s, 1H, br), 4.14-4.05 (m, 1H), 4.00-3.98 (m, 2H) 3.95 (s, 3H), 3.89 (t, *J* = 6.4 Hz, 2H) 2.91-2.88 (m, 2H), 2.73-2.70 (m, 2H), 2.60-2.57 (m, 2H), 2.39 (s, 3H), 2.35 (s, 3H), 2.21-2.16 (m, 4H), 2.06-2.00 (m, 2H), 1.69-1.60 (m, 2H); HRMS (+ESI) m/z calcd for $C_{28}H_{39}N_6O_2$ 491.3134, found, 491.3115.

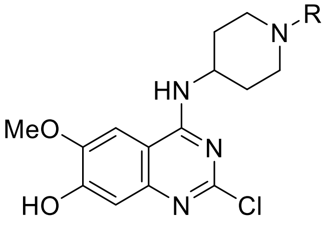


**99**
R = Bn

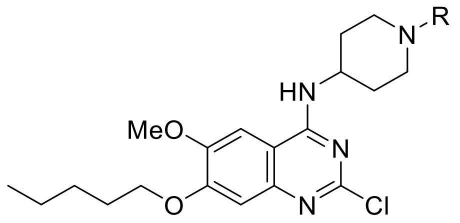


**100**
R = Bn

A solution of **98** (R = Bn) (1.16 g, 2.37 mmol) in TFA was refluxed for 3 h after which the volatiles were removed *in vacuo*. The dark residue was taken in saturated sodium bicarbonate solution and sonicated for a few minutes. The resulting solid was filtered, washed with water and ether and dried *in vacuo* to yield **99** (R = Bn) as a pale white solid which was used as such in next step. A mixture of **99** (0.485 g, 1.216 mmol), iodopentane (0.17 mL, 1.337 mmol) and potassium carbonate (0.84 g, 6.08 mmol) in anhydrous DMF (3 mL) was heated at 80 $^o$C for 6 h (reaction not complete). The reaction mixture was cooled to room temperature, diluted with ethyl acetate and filtered to remove solids. The

filtrate was washed with brine, dried over magnesium sulphate and concentrated *in vacuo* to obtain brown residue that was purified by silica gel column chromatography (DCM : MeOH (7 N $NH_3$); 100 : 0 → 95 : 5) to obtain **100** (R = Bn) yellow solid (79 mg, 13 %). $^1$H NMR (400 MHz, $CDCl_3$) δ 7.35-7.29 (m, 5H), 7.11 (s, 1H), 6.86 (s, 1H), 5.54 (d, *J* = 7.8 Hz, 1H), 4.35-4.26 (m, 1H), 4.11 (t, *J* = 6.8 Hz, 2H), 3.97 (s, 3H), 3.57 (s, 2H), 2.94-2.90 (m, 2H), 2.25 (t, *J* = 11.6 Hz, 2H), 2.15-2.13 (m, 2H), 1.94-1.87 (m, 2H), 1.70-1.60 (m, 2H), 1.50-1.37 (m, 4H), 0.95 (t, *J* = 7.1 Hz, 3H); HRMS (+ESI) m/z calcd for $C_{26}H_{34}N_4O_2Cl$ 469.2370, found, 469.2366.

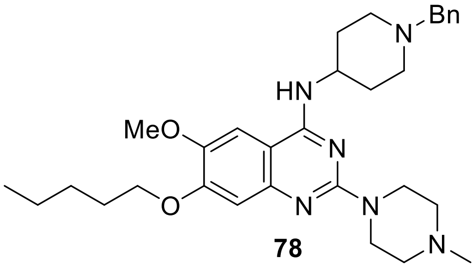


**78**

A mixture of **100** (R = Bn) (79 mg, 0.168 mmol), N-methylpiperazine (34 mg, 0.337 mmol) and HCl (0.08 mL, 4 M in dioxane) in isopropanol (3 mL) was heated in microwave at 160 °C for 15 min. The reaction mixture was allowed to cool at room temperature and volatiles were removed *in vacuo* to obtain dark residue that was dissolved in DCM. The organic layer was washed with saturated $NaHCO_3$ solution, dried over magnesium sulphate and evaporated *in vacuo* to obtain crude product that was purified over neutral alumina (DCM : MeOH (7 N $NH_3$); 100 : 0 → 98 : 2) to obtain **78** as a yellow solid (51 mg, 57 %). $^1$H NMR (400 MHz, $CDCl_3$) δ 7.37-7.29 (m, 5H), 6.91 (s, 1H), 6.72(s, 1H), 5.01 (d, *J* = 7.0 Hz, 1H), 4.20-4.09 (m, 3H), 3.94 (s, 3H), 3.90-3.88 (m, 4H), 3.58 (s, 2H), 2.95-2.92 (m, 2H), 2.53-2.50 (m, 4H), 2.37 (s, 3H), 2.26-2.16 (m, 4H), 1.95-1.88 (m, 2H), 1.69-1.60 (m, 2H), 1.48-1.38 (m, 4H), 0.95 (t, *J* = 7.1 Hz, 3H); HRMS (+ESI) m/z calcd for $C_{31}H_{45}N_6O_2$ 533.3604, found, 533.3616.

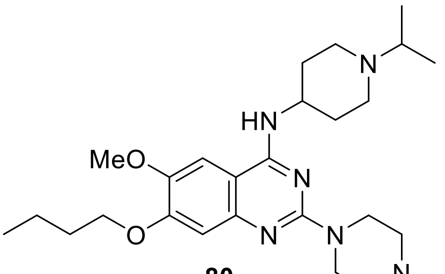


**80**

**80** was synthesized from **100** (R = *i*Pr) (0.229 g, 0.564 mmol) and N-methylpiperazine (0.125 mL, 1.128 mmol) and HCl (0.28 mL, 4 M in dioxane) following a procedure similar to the synthesis of **78**. It was obtained as a yellow solid (0.180 g, 68 %). $^1$H NMR (400 MHz, Methylene Chloride-d2) δ 6.83 (s, 1H), 6.78 (s, 1H), 5.09 (d, *J* = 7.3 Hz, 1H), 4.19-4.03 (m, 3H), 3.92 (s, 3H), 3.82 (dd, *J* = 6.4, 3.9 Hz, 4H), 2.91 (d, *J* = 11.8 Hz, 2H), 2.80 (p, *J* = 6.6 Hz, 1H), 2.45 (t, *J* = 5.1 Hz, 4H), 2.38 (td, *J* = 11.5, 2.5 Hz, 2H), 2.32 (s, 3H), 2.18 (d, *J* = 12.0 Hz, 2H), 1.93-1.79 (m, 2H), 1.62-1.47 (m, 4H), 1.08 (s, 3H), 1.07 (s, 3H), 1.03 (t, *J* = 7.4 Hz, 3H); HRMS (+ESI) m/z calcd for $C_{26}H_{43}N_6O_2$ 471.3448, found, 471.3451.

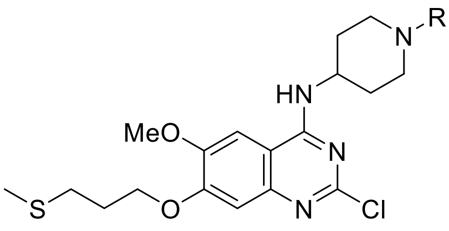


**100**
R = Bn

A mixture of **99** (40 mg, 0.1 mmol), 3-(Methylthio)-1-propanol (0.041 mL, 0.4 mmol) and triphenylphosphine (0.131 g, 0.5 mmol) in anhydrous THF (2 mL) was cooled to 0 °C followed by the addition of diisopropyl azodicarboxylate (DIAD) (0.098 mL, 0.5 mmol). The reaction mixture was allowed to stir at room temperature for 20 h. Afterwards, THF was removed, residue dissolved in DCM and organic layer was washed successively with water and brine. The organic layer was then dried over magnesium sulphate and concentrated *in vacuo* to obtain brown residue that was purified by

silica gel column chromatography (DCM : MeOH (7 N $NH_3$); 100 : 0 → 95 : 5) to yield **100** (R = Bn) as a brown solid (20 mg, 41 %). $^1$H NMR (400 MHz, $CDCl_3$) δ 7.37-7.32 (m, 5H), 7.15 (s, 1H), 6.79 (s, 1H), 5.35 (d, *J* = 7.7 Hz, 1H), 4.36-4.29 (m, 1H), 4.24 (t, *J* = 6.3 Hz, 2H), 3.99 (s, 3H), 3.59 (s, 2H), 2.95-2.92 (m, 2H), 2.73 (t, *J* = 7.1 Hz, 2H), 2.31-2.15 (m, 8H), 1.71-1.61 (m, 2H).

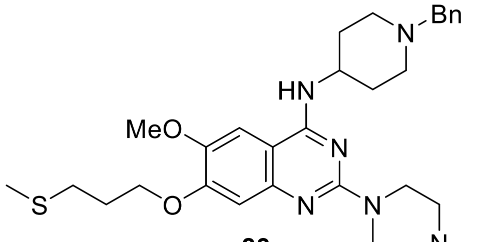


**83**

A mixture **100** (R = Bn) (20 mg, 0.041 mmol) and N-methyl piperazine (0.045 mL, 0.41 mmol) in toluene (0.5 mL) was heated at 130 °C for 50 min under microwave irradiation. The reaction mixture was concentrated and purified by silica gel column chromatography (DCM : MeOH (7 N $NH_3$); 100 : 0 → 95 : 5) to obtain the final product **83** as a yellow solid (20 mg, 88 %). $^1$H NMR (400 MHz, $CDCl_3$) δ 7.37-7.30 (m, 5H), 6.92 (s, 1H), 6.71 (s, 1H), 4.98 (d, *J* = 7.1 Hz, 1H), 4.22 (t, *J* = 6.4 Hz, 2H), 4.18-4.11 (m, 1H), 3.93 (s, 3H), 3.88-3.83 (m, 4H), 3.58 (s, 2H), 2.95-2.92 (m, 2H), 2.72 (t, *J* = 7.1 Hz, 2H), 2.52-2.50 (m, 4H), 2.37 (s, 3H), 2.26-2.14 (m, 8H), 1.69-1.59 (m, 2H); HRMS (+ESI) m/z calcd for $C_{30}H_{43}N_6O_2S$ 551.3168, found, 551.3171.

# NOE analysis of diaminoisoquinolines

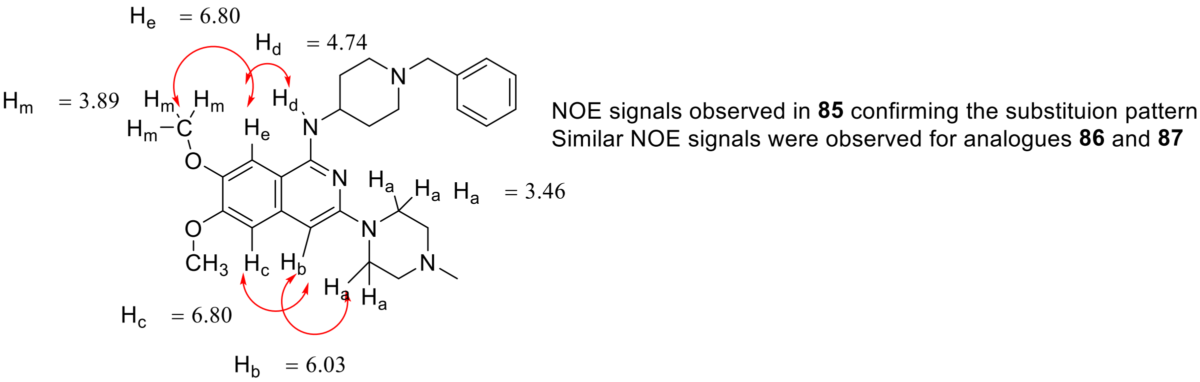


NOE signals observed in **85** confirming the substituion pattern
Similar NOE signals were observed for analogues **86** and **87**

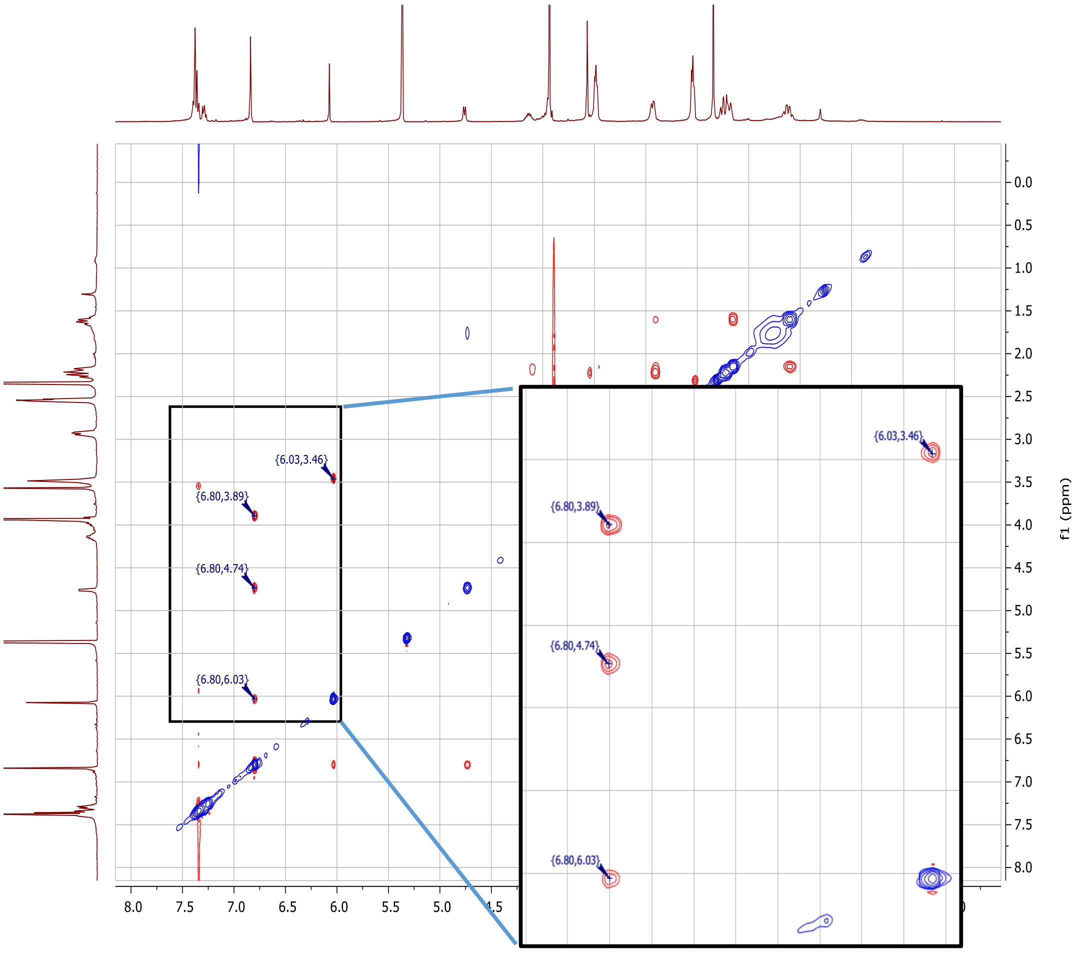

# NOE analysis/spectral data for diaminoquinolines intermediates

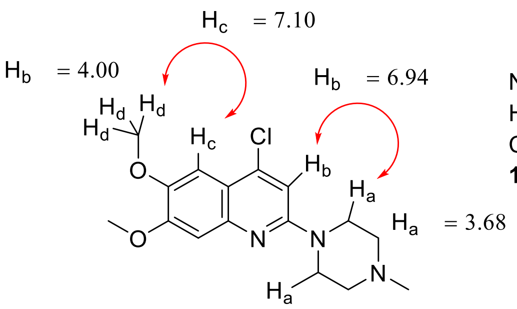


NOE signals observed in **111a** confirming the substitution pattern
$H_a$ shows cross-peak only with $H_b$ and not $H_c$
Crystal structure of **111a** confirms this structure
**111b** and **111c** show similar cross-peaks

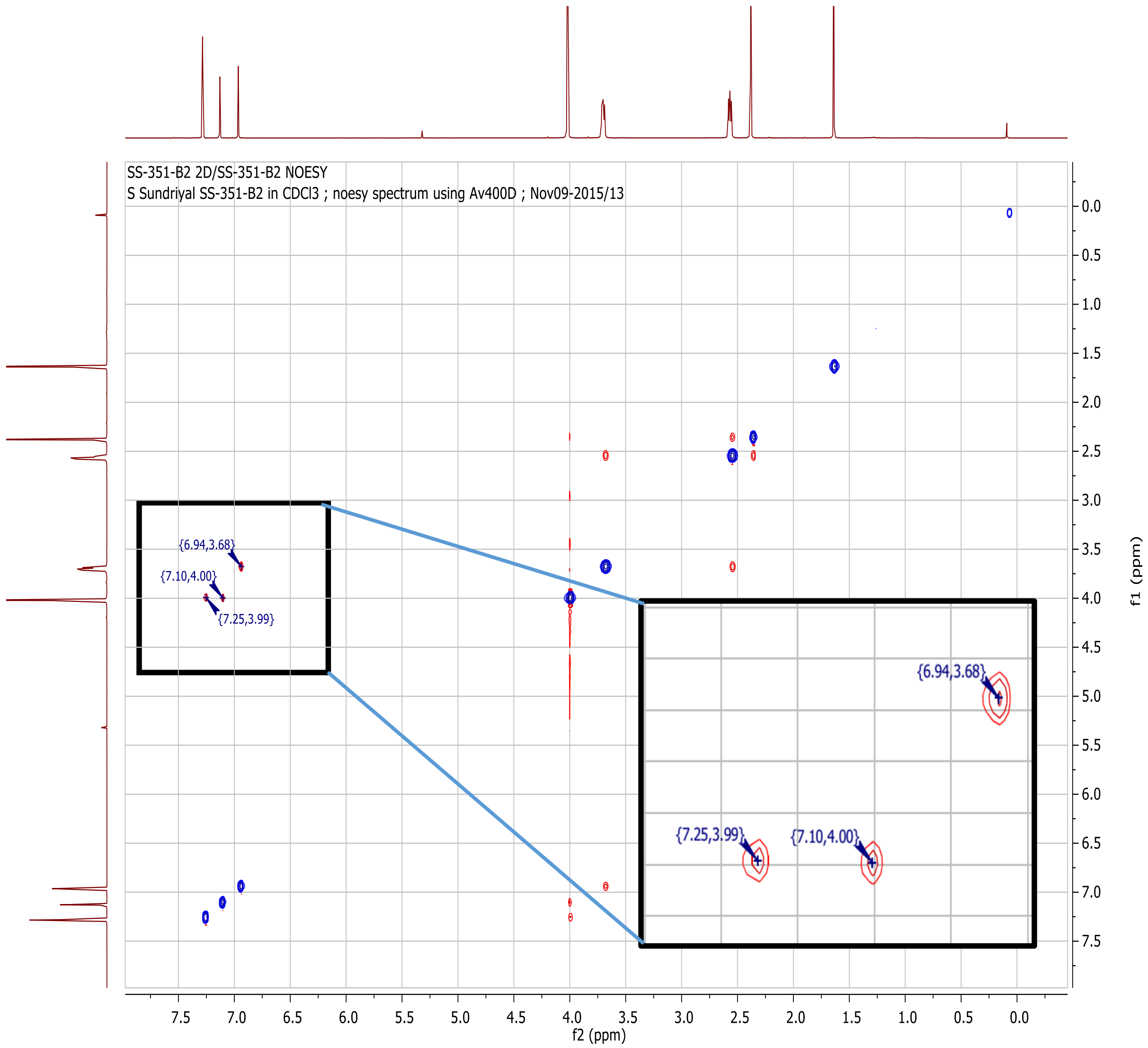

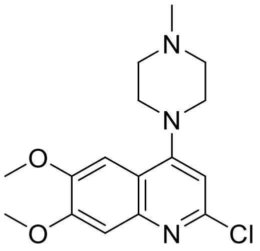

**Regioisomer of 111a**

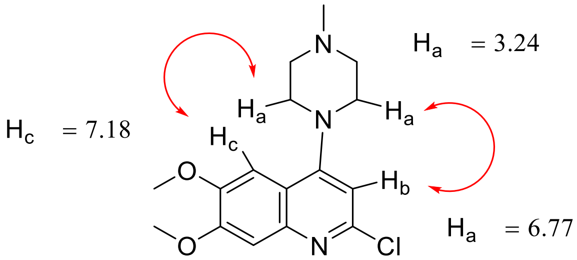


$^{1}$H NMR (400 MHz, Chloroform-d) δ 7.31 (s, 1H), 7.18 (s, 1H), 6.77 (s, 1H), 3.99 (s, 6H), 3.24 (t, *J* = 5.0 Hz, 4H), 2.70 (t, *J* = 4.8 Hz, 4H), 2.42 (s, 3H); $^{13}$C NMR (101 MHz, $CDCl_3$) δ 157.78, 152.55, 149.24, 148.85, 145.72, 117.01, 108.57, 108.23, 101.90, 56.11, 55.89, 54.95, 51.73, 46.05; HRMS (+ESI) m/z calcd for $C_{16}H_{21}ClN_2O_3$, 322.1322, found, 322.1326.

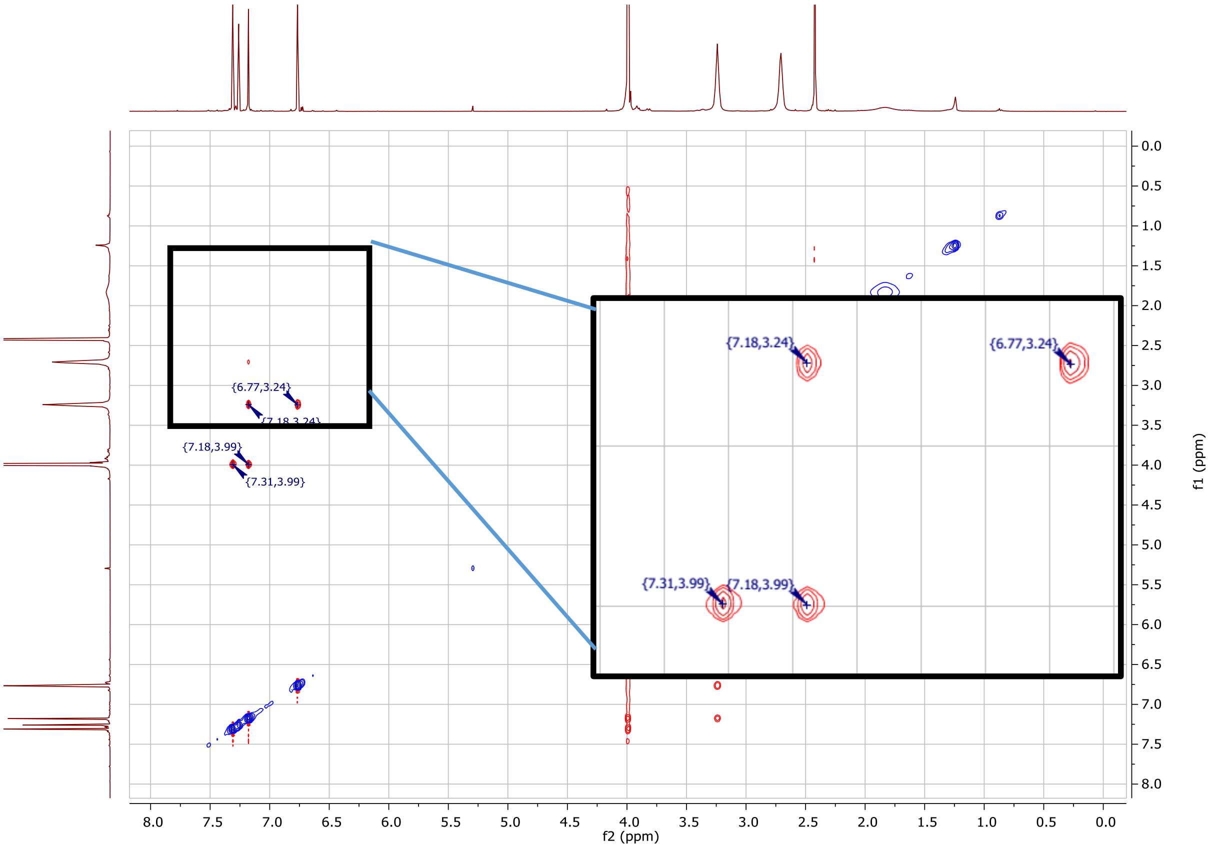

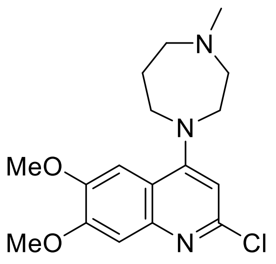

**Regioisomer of 111b**

$^{1}$H NMR (500 MHz, Chloroform-d) δ 7.27 (s, 1H), 7.18 (s, 1H), 6.67 (s, 1H), 3.97 (s, 6H), 3.64-3.62 (m, 2H), 3.57-3.54 (m, 2H), 2.82-2.80 (m, 2H), 2.77-2.75 (m, 2H), 2.44 (s, 3H), 2.09-2.05 (m, 2H); $^{13}$C NMR (126 MHz, $CDCl_3$) δ 158.27, 152.10, 149.17, 147.86, 146.03, 116.25, 108.20, 106.84, 103.32, 58.27, 57.50, 56.02, 55.94, 54.16, 52.95, 46.96, 28.00; HRMS (+ESI) m/z calcd for $C_{17}H_{23}ClN_2O_3$, 336.1479, found, 336.1479.

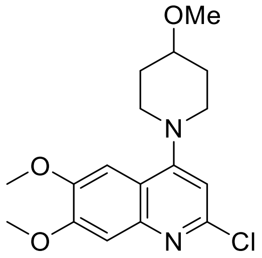

**Regioisomer of 111c**

This regioisomer of **111c** could not be isolated in high purity by column chromatography and only a small amount was isolated for characterization purpose using preparative TLC (EtOAc : Pet. ether; 1 : 2). $^{1}$H NMR (400 MHz, Methylene Chloride-d2) δ 7.24 (s, 1H), 7.17 (s, 1H), 6.74 (s, 1H), 3.95 (s, 6H), 3.47-3.40 (m, 2H), 3.38 (m, 3H), 2.96 (ddd, *J* = 12.3, 9.2, 3.0 Hz, 2H), 2.12 (ddd, *J* = 12.8, 6.5, 3.4 Hz, 2H), 1.88-1.79 (m, 2H); HRMS (+ESI) m/z calcd for $C_{17}H_{22}ClN_2O_3$, 337.1319, found, 337.1324.

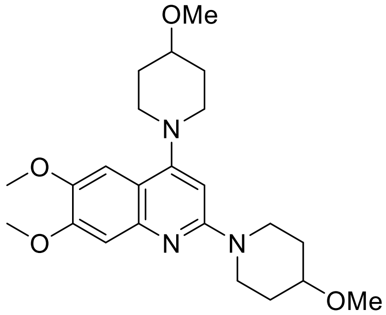

**Disubstituted analouge of 111c**

Isolated from the synthesis of **111c** as a yellow solid (85 mg). $^{1}$H NMR (400 MHz, Chloroform-d) δ 7.10 (s, 2H), 6.37 (s, 1H), 4.10 (dt, *J* = 13.1, 4.7 Hz, 2H), 3.98 (s, 3H), 3.94 (s, 3H), 3.45-3.37 (m, 10H), 3.27-3.19 (m, 2H), 2.92-2.88 (m, 2H), 2.13 (ddt, *J* = 13.1, 6.3, 3.4 Hz, 2H), 2.07-1.99 (m, 2H), 1.86 (dtd, *J* = 12.5, 8.6, 3.5 Hz, 2H), 1.69-1.62 (m, 2H); $^{13}$C NMR (126 MHz, $CDCl_3$) δ 158.02, 157.65, 151.78, 145.95, 145.54, 112.96, 107.11, 102.40, 96.00, 76.64, 76.05, 55.92, 55.73, 55.59, 49.76, 43.47, 31.22, 30.67; HRMS (+ESI) m/z calcd for $C_{23}H_{34}N_3O_4$, 416.2549, found, 416.2545.

# X-ray crystal structure of 111a

*Crystal data for* **111a**: $C_{16}H_{20}ClN_3O_2$, $M$ = 321.80, monoclinic, $P2_1/c$ (no. 14), $a$ = 9.9885(4), $b$ = 8.6548(4), $c$ = 18.3874(7) Å, β = 103.256(4)°, $V$ = 1547.21(11) Å$^3$, $Z$ = 4, $D_c$ = 1.381 g cm$^{-3}$, μ(Mo-Kα) = 0.258 mm$^{-1}$, $T$ = 173 K, colourless plates, Agilent Xcalibur 3 E diffractometer; 3074 independent measured reflections ($R_{int}$ = 0.0195), $F^2$ refinement,[5, 6] $R_1$(obs) = 0.0379, $wR_2$(all) = 0.0900, 2522 independent observed absorption-corrected reflections [$|F_o| > 4\sigma(|F_o|)$, $2\theta_{full}$ = 50°], 203 parameters. CCDC 1503377.

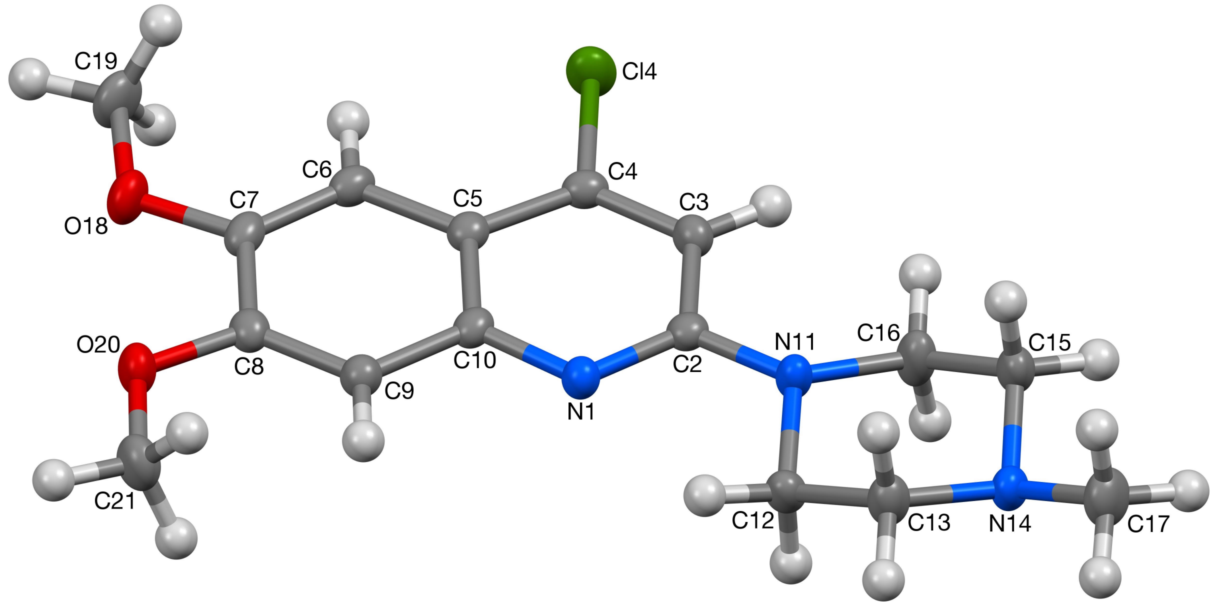


**Fig. SF1** The crystal structure of **111a** (50 % probability ellipsoids).

# References

1. S. Sundriyal, N. A. Malmquist, J. Caron, S. Blundell, F. Liu, X. Chen, N. Srimongkolpithak, J. Jin, S. A. Charman, A. Scherf and M. J. Fuchter, *ChemMedChem*, 2014, **9**, 2360-2373.
2. F. Liu, X. Chen, A. Allali-Hassani, A. M. Quinn, T. J. Wigle, G. A. Wasney, A. Dong, G. Senisterra, I. Chau, A. Siarheyeva, J. L. Norris, D. B. Kireev, A. Jadhav, J. M. Herold, W. P. Janzen, C. H. Arrowsmith, S. V. Frye, P. J. Brown, A. Simeonov, M. Vedadi and J. Jin, *J. Med. Chem.*, 2010, **53**, 5844-5857.
3. F. Liu, X. Chen, A. Allali-Hassani, A. M. Quinn, G. A. Wasney, A. Dong, D. Barsyte, I. Kozieradzki, G. Senisterra, I. Chau, A. Siarheyeva, D. B. Kireev, A. Jadhav, J. M. Herold, S. V. Frye, C. H. Arrowsmith, P. J. Brown, A. Simeonov, M. Vedadi and J. Jin, *J. Med. Chem.*, 2009, **52**, 7950-7953.
4. N. Srimongkolpithak, S. Sundriyal, F. Li, M. Vedadi and M. J. Fuchter, *MedChemComm*, 2014, **5**, 1821-1828.
5. SHELXTL, Bruker AXS, Madison, WI.
6. G. Sheldrick, *Acta Crystallographica Section C*, 2015, **71**, 3-8.
